# Supplementary material for: Scalable integrated two-dimensional Fourier-transform spectrometry
Source: Nat Commun. 2024 Jan 10;15:436. doi: 10.1038/s41467-023-44518-y (PMC10781879; doi:10.1038/s41467-023-44518-y)
Supplement: Supplementary file 1 — Supplementary information for Scalable integrated two-dimensional Fourier-transform spectrometry [file 41467_2023_44518_MOESM1_ESM.pdf]

Supplementary information for:

Scalable integrated two-dimensional Fourier-transform spectrometry

Hongnan Xu*, Yue Qin, Gaolei Hu, and Hon Ki Tsang*

Department of Electronic Engineering, The Chinese University of Hong Kong, Shatin, New Territories, Hong Kong SAR, China

*Corresponding authors, E-mail: hongnanxu@cuhk.edu.hk, hktsang@ee.cuhk.edu.hk

Contents:

Note 1. Performance comparison of integrated spectrometers

Note 2. Abbreviations and notations

Note 3. Design and characterization of the tunable delay line

Note 4. Design and characterization of the adiabatic directional coupler

Note 5. Design and characterization of the Y-branch splitter

Note 6. Design and characterization of the spectrometer

Note 7. Analysis of temperature sensitivity

Note 8. Reconstruction method

Note 9. Extended experimental data

Note 10. Discussion of scheme scalability

Note 11. Comparison of spectrometry schemes

Note 1. Performance comparison of integrated spectrometers

Table S1. Comparison of reported integrated spectrometers.

Design	Platform	Footprint [μm^2]	$\delta\lambda_{res}$ [pm] (a)	BW [nm] (b)	P_a [mW]	N_{ch} (c)
AWG[S1]	SOI	8000×8000	150	7.5	/	50
EDG[S2]	SOI	6000×9000	500	30	/	60
EDG[S3]	SOI	2×10^{12}	150	148	/	926
MRR[S4]	SOI	1×10^{6}	600	50	/	81
PhC cavity[S5]	SOI	6×111	1000	35	/	38
PhC cavity[S6]	SOI	18×250	160	16	30×3	101
WBG[S7]	SOI	3.6×10^{4}	510	102.7	873	201
AWG/MRR[S8]	SOI	3000×3000	100	25.4	30×9	255
AWG/MRR[S9]	SNOI	1150×1250	750	57.5	/	70
AWG/MRR[S10]	SOI	200×270	200	70	NM (d)	350
MRR[S11]	SOI	3.5×10^{5}	5	10	50×10	1941
MRR[S12]	SOI	20×35	80	100	45	1251
MRR[S13]	SOI	60×60	40	100	75	2501
MDR[S14]	SOI	200×200	200	20	160	101
SS[S15]	SOI	100×50	600	25	/	42
SS[S16]	SNOI	200×50	3400	40	/	13
SS[S17]	SOI	12.8×30	250	30		121
SS[S18]	SOI	35×260	450	180	/	401
SS[S19]	SOI	500×500	10	2	/	332
SS[S20]	SOI	1600×2100	16	2	/	126
SS[S21]	SOI	> 2000×2000	100	6.3	/	64
SS[S22]	SOI	> 2000×2000	20	100	64×6	5001
SS[S23]	SNOI	220×520	20	12	/	600
SS[S24]	SOI	88×300	100	120	/	160
SS[S25]	SOI	1000×1000	15	40	/	> 2500
SS[S26]	SOI	2000×7600	30	115	NM (d)	> 3800
SS[S27]	SOI	1000×1500	5	100	50×14/2 (e)	2×10^{4}
SS[S28]	SOI	310×215	200	60	33	301
SHS[S29]	Silica	2500×4300	320	5.12	/	16
SHS[S30]	Silica	NM (d)	16	0.512	/	32
SHS[S31]	SOI	NM (d)	50	≈ 0.8	/	16
SHS[S32,33]	SOI	1.2×10^{7}	40	≈ 0.64	/	16
SHS[S34]	SOI	NM (d)	48	0.78	/	16

SHS[S35]	SOI	1100×1500	6000	600	/	100
SHS[S36]	SNOI	NM [d]	≈ 49	≈ 0.39	/	8
SHS[S37]	SOI	7100×18000	40000 [e]	400 [e]		≈ 10 [f]
SHS[S38]	SNOI	650×4700	5000 [f]	400 [f]	/	> 80 [g]
SHS[S39]	SNOI	4600×5800	400	≈ 6.4	/	16
tFTS[S40]	LNOI	NM [d]	≈ 70000	450	NM [d]	> 6
tFTS[S41]	SOI	1×10^6	3000	> 50	5100	≈ 20
tFTS[S42]	SOI	NM [d]	320 [g]	180 [g]	5000	> 560 [h]
dFTS[S43]	SOI	NM [d]	400	> 20	33×6	32
dFTS[S44]	SOI	2500×3500	50	3.2	30×7	≈ 64
SWIFTS[S45]	SOI	22×512	4000	96	/	25
SWIFTS[S46]	SNOI	1×10^5	6000	> 100	/	> 16
SWIFTS[S47]	LNOI	1×10^7	≈ 5000	500	NM [d]	101
tFTS & MRR[S48]	SOI	NM [d]	470 [i]	90	> 1800	> 190
**This work**	**SOI**	**5500×6000**	**250**<br>**125** [j]	**200**	**2.4**	**801**<br>**1601** [j]

The blue shade represents all reported Fourier-transform spectrometers. The grey shade represents other types of spectrometers. The red shade represents this work.

AWG, arrayed waveguide grating.

EDG, echelle diffraction grating.

MRR, microring resonator.

MDR, microdisk resonator.

PhC, photonic crystal.

WBG, waveguide Bragg grating.

SS, speckle spectrometer.

FTS, Fourier-transform spectrometer.

SHS, spatial heterodyne spectrometer.

tFTS, tunable FTS.

dFTS, digital FTS.

SWIFTS, stationary-wave integrated FTS.

SOI, silicon on insulator.

SNOI, silicon nitride on insulator.

LNOI, lithium niobate on insulator.

$\delta\lambda_{res}$, spectral resolution.

BW, working bandwidth.

N_{ch}, channel capacity defined by $N_f = BW/\Delta\lambda_{res}$.

P_a, aggregate power consumption.

[a]$\delta\lambda_{res}$ is determined based on the Rayleigh criterion (i.e., $\delta\lambda_f$).

[b]BW is derived using $BW = N_{ch}\delta\lambda_{res}$.

[c]For the Fourier-transform spectrometers in which the number of Mach-Zehnder interferometers (MZI) is stated, N_{ch} is half of the MZI number; otherwise, N_{ch} is derived from half of the gird number in the computationally reconstructed spectrum. For other spectrometer schemes, N_{ch} is defined as the bandwidth-to-resolution ratio.

[d]Not mentioned.

[e]Since the applied heating power is a random sequence with the average of 0.5, the power consumption is halved here.

[f]Operated at mid-infrared wavelengths.

[g]Several narrow bands are bridged to form the whole BW by changing the polarization and incident angle of light.

[h]Three parallel Michelson interferometers (MI) are employed to improve $\delta\lambda_{res}$ and expand BW.

[i]Here, $\delta\lambda_{res}$ is defined as the linewidth of the MRR.

[j]The resolution and capacity can be improved to $\delta\lambda_{2f} = \delta\lambda_{f}/2$ and $N_{2f} = 2N_{f}$, respectively, using the computational method.

Note 2. Abbreviations and notations

All abbreviations and notations used in the main manuscript are defined as follows:

SOI: silicon-on-insulator.
1D/2D-FTS: one/two-dimensional Fourier-transform spectrometer.
SHS: spatial heterodyne spectrometer.
tFTS: tunable Fourier-transform spectrometer.
dFTS: digital Fourier-transform spectrometer.
SWIFTS: stationary-wave integrated Fourier-transform spectrometer.
PD: photodetector.
MI: Michelson interferometer.
MZI: Mach-Zehnder interferometer.
MRR: microring resonator.
PS: power splitter.
ADC: adiabatic coupler.
YBS: Y-branch splitter.
GC: grating coupler.
FBG: fiber Bragg grating.
AWG: arrayed waveguide grating.
IO: input and output.
IN_i: *i*-th input port of the 2D-FTS.
OUTi: *i*-th output port in the spectrometer.
TV: total variation.
OSA: optical spectrum analyzer.
TL: tunable laser.
ASE: amplified spontaneous emission.
TEC: thermo-electric cooler.

FFT(·): fast Fourier transform.
DCT(·): discrete cosine transform.
$\rho(\cdot, \cdot)$: correlation between fringes.
argmin(·): global minimum.
A: transmittance cube.
S: unknown spectrum.
O: recorded interferogram.
$\tilde{\mathbf{O}}$: interferogram with zero-frequency components removed.
$\mathbf{a}_i$: fringe at the *i*-th wavelength channel in **A**.
$\tilde{\mathbf{a}}_i$: *i*-th fringe with zero-frequency components removed.
$\tilde{\mathbf{A}}_{FFT}$: matrix formed by the column vectors of FFT($\tilde{\mathbf{a}}_i$).
Ω: regularization term.
λ: wavelength.
P: electric power applied to the heater.
P_{max}: maximum heating power required to establish decorrelation.
H_{wg}: height of the waveguide.

H_{ht}: height of the heater.
W_{wg}: width of the waveguide.
W_{ht}: width of the heater.
d_{ht}: spacing between the waveguide and the heater.
OPL: optical path length.
L_{tFTS}: arm length of the tFTS.
$\Delta L_{SHS,i}$: arm-length difference of the i-th MZI in the SHS.
$\Delta L_{SHS,max}$: maximum arm-length difference in the SHS.
L_{ADC}: coupling length of the ADC.
L_{YBS}: coupling length of the YBS.
f_{tFTS}: Fourier frequency provided by the tFTS.
f_{SHS}: Fourier frequency provided by the SHS.
$\delta\lambda_{tFTS}$: spectral resolution of the tFTS.
$\delta\lambda_{SHS}$: spectral resolution of the SHS.
$\delta\lambda_{f}$: spectral resolution defined at the Rayleigh criterion.
$\delta\lambda_{2f}$: spectral resolution supported by the computational method.
N_{tFTS}: number of power sweep steps.
N_{SHS}: number of MZIs in the SHS.
N_{f}: channel capacity defined at the Rayleigh criterion.
N_{2f}: channel capacity supported by the computational method.
FSR_{tFTS}: free spectral range of the tFTS.
FSR_{SHS}: free spectral range of the SHS.
BW: operation bandwidth.
PSNR: peak signal-to-noise ratio.
n_g: group index.
Δn_g: group-index change induced by heating.
$\partial n_g/\partial P$: first-order tuning efficiency.
$\partial^2 n_g/\partial P^2$: second-order tuning efficiency.
$|t|^2$: transmittance.
$|\kappa_{ADC}|^2$: coupling ratio of the ADC.
$|\kappa_{YBS}|^2$: coupling ratio of the YBS.
ε: relative error.
r^2: coefficient of determination.

Note 3. Design and characterization of the tunable delay line

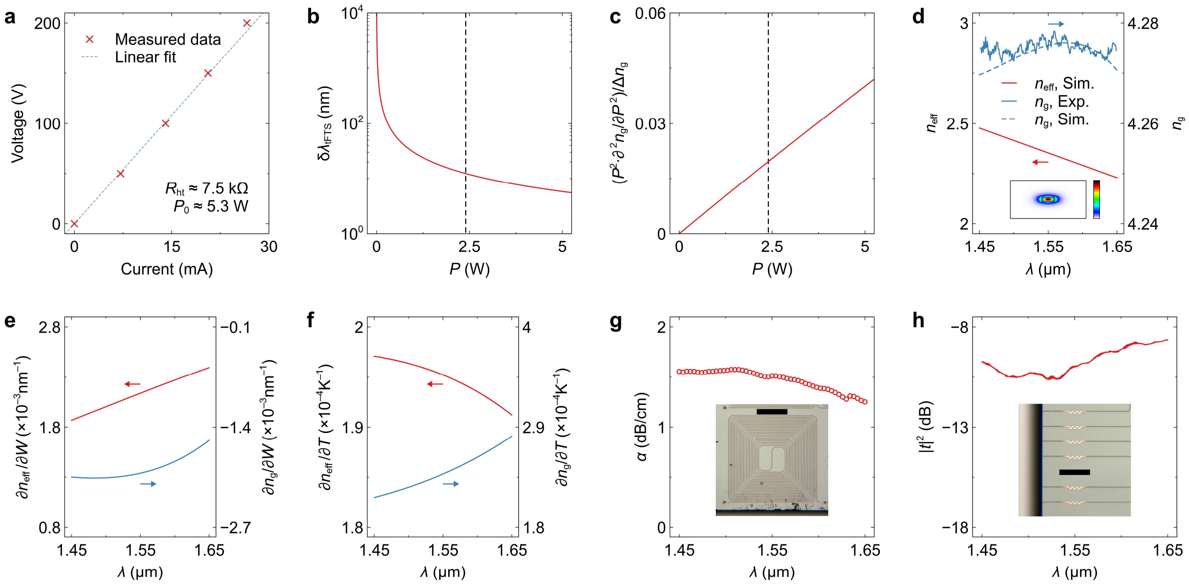

Figure S1 Tunable delay line. (**a**) Measured voltage with varying current. By applying linear fit, the heater resistance is measured to be $R_{ht} \approx 7.5$ kΩ. At the maximum voltage of 200 V, the heating power of $P_0 \approx 5.3$ W is attained, which exceeds the critical value required ($P_{max} \approx 2.4$ W). (**b**) Calculated resolution ($\delta\lambda_{tFTS}$) of the tunable Fourier-transform spectrometer (tFTS) with varying heating power (P). Here, the used tuning-efficiency data is shown in Figure 2d. (**c**) Calculated contribution of the second-order thermo-optical nonlinearity [$(P^2 \cdot \partial^2 n_g/\partial P^2)/\Delta n_g$] with varying P. Here, the dashed lines represent $P = 2.4$ W. (**d**) Calculated effective indices (n_{eff}) and group indices (n_g) at varying wavelengths (λ). The measured n_g is also plotted. The inset shows the calculated electric-field profile of the TE_0 mode. The dimension of the cross section is $W_{wg} \times H_{wg} = 450 \times 220$ nm^2. The color bar ranges from $|E|^2 = 0$ to 1. (**e**) Calculated width sensitivities ($\partial n_{eff}/\partial W$, $\partial n_g/\partial W$) at varying λ. (**f**) Calculated temperature sensitivities ($\partial n_{eff}/\partial T$, $\partial n_g/\partial T$) at varying λ. (**g**) Measured propagation loss (α) at varying λ. The inset shows the microscope image of the fabricated testing spiral waveguide. The scale bar represents 150 μm. (**h**) Measured end-to-end coupling efficiencies ($|t|^2$) at varying λ. The inset shows the microscope image of the fabricated edge couplers and polarizers. The scale bar represents 100 μm.

The tunable Fourier-transform spectrometer (tFTS) has a pair of identical spiral waveguides with heaters atop, which function as tunable delay lines, as shown in Figures 2a and 2b. By applying heating power (P) to one of the delay lines, the effective path-length difference increases, resulting in the sinusoidal response, as shown in Figure 2c. The silicon-on-insulator (SOI) waveguide has a cross-section of $W_{wg} \times H_{wg} = 450 \times 220$ nm^2 to support a single TE_0 mode. The heater is a titanium-tungsten (TiW) alloy strip with $W_{ht} \times H_{ht} = 7 \times 0.2$ μm^2. The spacing to the waveguide top is set as $d_{ht} = 1$ μm. Figure S1a shows the measured voltage-to-current curve. The measured resistance of the 1.5-cm long heater is $R_{ht} \approx 7.5$ kΩ. The large R_{ht} reduces the current and prevents it from melting down. The voltage source (Keithley 2400) used in the experiment provides a maximum voltage of 200 V, thereby supporting $P_0 \approx 5.3$ W. Both simulation and experimental results suggest that the maximum heating power required to establish decorrelation is $P_{max} < 2.4$ W (see Note 6), with the corresponding voltage of ≈ 135 V. Consequently, the entire power sweep range can be covered.

The resolution ($\delta\lambda_{tFTS}$) of the tFTS is tied to the maximum $\Delta n_g L_{tFTS}$. Here, Δn_g denotes the group-index change induced by thermo-optical (TO) tuning, and L_{tFTS} (= 1.5 cm) denotes the spiral length. We first give the derivation of $\delta\lambda_{tFTS}$. The m-th and (m + 1)-th order constructive interference can be formulated as:

$$n_{eff}(P_{max})L_{tFTS} - n_{eff}(0)L_{tFTS} = m\lambda, \tag{S1}$$

$$\left[n_{eff}(P_{max}) + \delta\lambda_{tFTS}\frac{\partial n_{eff}(P_{max})}{\partial\lambda}\right]L_{tFTS} - \left[n_{eff}(0) + \delta\lambda_{tFTS}\frac{\partial n_{eff}(0)}{\partial\lambda}\right]L_{tFTS} = m(\lambda+1), \tag{S2}$$

where $n_{eff}(P)$ denotes the effective index at the heating power of P, and λ denotes the wavelength. $\delta\lambda_{tFTS}$ can be thus derived by subtracting Equation S1 from S2:

$$\delta\lambda_{tFTS} = \frac{\lambda^2}{L_{tFTS}\left[n_{eff}(P_{max}) - n_{eff}(0) - \lambda\frac{\partial n_{eff}(P_{max})}{\partial\lambda} + \lambda\frac{\partial n_{eff}(0)}{\partial\lambda}\right] - \lambda}. \tag{S3}$$

The group index is defined as $n_g = n_{eff} - \lambda\cdot\partial n_{eff}/\partial\lambda$, which yields:

$$\delta\lambda_{tFTS} = \frac{\lambda^2}{L_{tFTS}\left[n_g(P_{max}) - n_g(0)\right] - \lambda} \approx \frac{\lambda^2}{\Delta n_g L_{tFTS}}. \tag{S4}$$

The TO nonlinearity arises at a high heating power. Hence, Δn_g contains higher-order terms[S41]:

$$\Delta n_g = \sum_i \frac{\partial^i n_g}{\partial P^i}\cdot P^i. \tag{S5}$$

In the analysis, we mainly consider the first- and second-order terms of the tuning efficiency, i.e., $\partial n_g/\partial P$ and $\partial^2 n_g/\partial P^2$. We measured $\delta\lambda_{tFTS}$ when uniformly varying the heating power from P = 2 W to 5 W. The FSRs of the sinusoidal response are coarse and non-uniform over the whole bandwidth. To address this issue, in the reconstruction, the sampled $\delta\lambda_{tFTS}$ are extrapolated into 51 points that are uniformly spaced from λ = 1.45 μm to 1.65 μm. Thus, $\partial n_g/\partial P$ and $\partial^2 n_g/\partial P^2$ can be resolved at each λ using Equations S4 and S5, as shown in Figure 2d. Figure S1b shows the calculated $\delta\lambda_{tFTS}$ with varying P and the tuning efficiency measured at λ = 1.55 μm. In Figure S1c, we calculate the contribution of the second-order TO nonlinearity. At P_{max} = 2.4 W, the fraction of the second-order term is $(P^2\cdot\partial^2 n_g/\partial P^2)/\Delta n_g \approx 2\%$.

The calculated dispersion curves of n_{eff} and n_g are shown in Figure S1d. The characterized n_g dispersions, which are derived from the interference curve shown in Figure 2g, is also plotted for comparison. The inset of Figure S1d is the calculated electric-field profile of the TE_0 mode. The simulation results are derived from a straight waveguide, provided that each SHS has the same number of bends in two arms and its dispersion is provided solely by the straight sections. In addition, for the high-contrast SOI waveguide, the variation of

dispersion is minimal at a modest bending radius of > 10 μm in the design. In Figures S1e and S1f, we show the calculated width sensitivities ($\partial n_{\mathrm{eff}}/\partial W$, $\partial n_{\mathrm{g}}/\partial W$) and temperature sensitivities ($\partial n_{\mathrm{eff}}/\partial T$, $\partial n_{\mathrm{g}}/\partial T$). Some testing spiral waveguides, with the lengths ranging from 2.5 cm to 7.5 cm, were fabricated on the same chip to measure the propagation loss (α). The loss is measured to be α < 1.5 dB/cm, as shown in Figure S1g. All the above results will be utilized in the modeling of the spectrometer (see Note 6). The edge couplers were employed to interface the lensed fibers and fabricated spectrometer. The TE-pass corner-mirror polarizers were monolithically integrated with the input edge couplers to ensure that the device works at the TE state. Our previous research has demonstrated that a high polarization extinction ratio of ≈ 40 dB can be obtained with such a structure[S49]. Figure S1h shows the measured end-to-end coupling efficiencies ($|t|^2$) at varying λ. Here, the testing structure consists of two edge couplers connected by a straight waveguide, with a polarizer inserted at the input. For clarity, the transmittance cube displayed in Figure 3e is normalized to its maximum. In the spectrum reconstruction, the excess losses caused by the power splitter, polarizer, and edge couplers must be added to the matrix, as they are spectrally non-uniform.

Note 4. Design and characterization of the adiabatic directional coupler

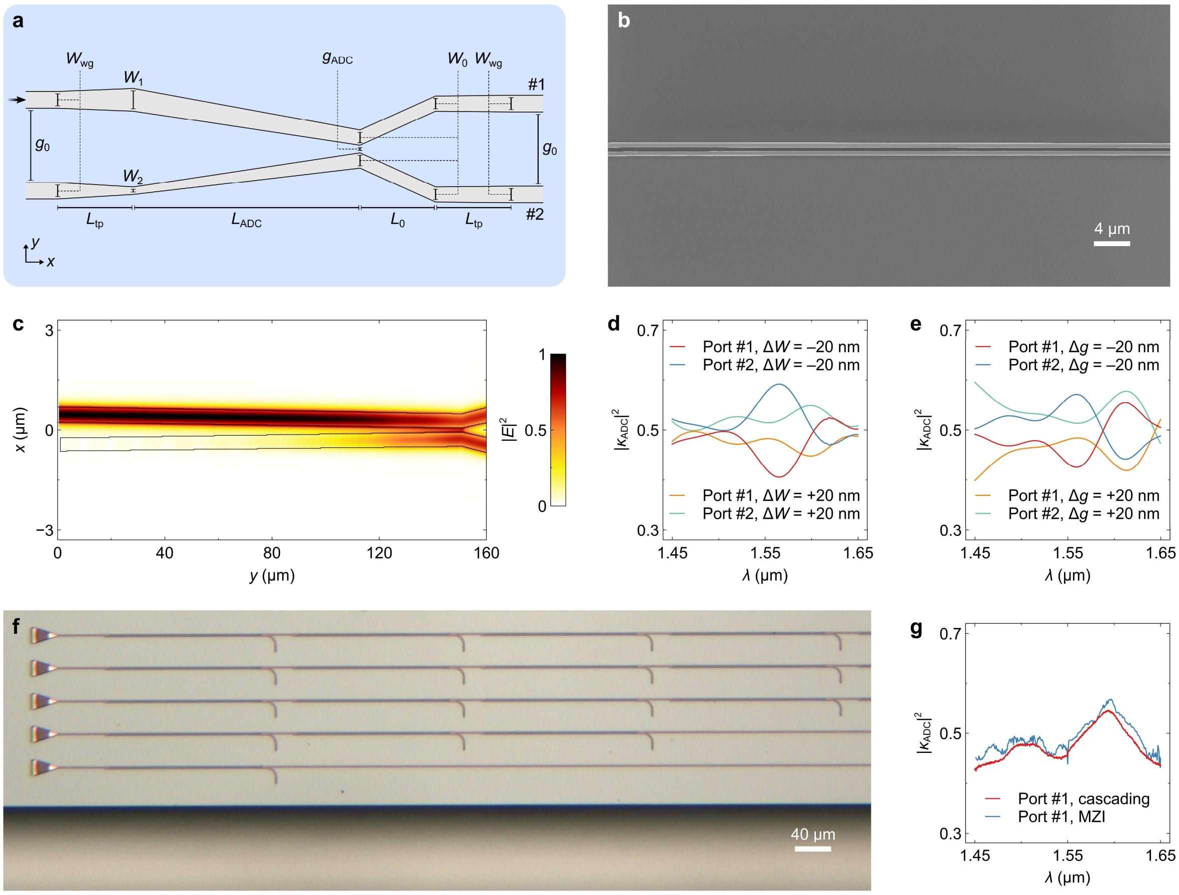

Figure S2 Adiabatic directional coupler (ADC). (**a**) Schematic illustration of the ADC with parameters labeled. (**b**) Scanning electron microscope image (SEM) of the fabricated ADC. (**c**) Calculated light propagation profile. Calculated coupling ratios ($|\kappa_{ADC}|^2$) with varying (**d**) core-width deviations (ΔW) and (**e**) gap width deviations (Δg) at varying wavelengths (λ). (**f**) Microscope image of the fabricated cascading structures. (**g**) Measured $|\kappa_{ADC}|^2$ dispersions at the port #1. The result measured from the Mach-Zehnder interferometer (MZI) is also plotted for comparison. The excess losses are deducted from the presented spectra.

The adiabatic directional coupler (ADC) is employed in the tunable Fourier-transform spectrometer (tFTS). The purpose of using a 2×2 coupler is to provide extra monitoring ports (i.e., IN_2 and OUT_0, see Figure 1a) for the tFTS. The port #2 is routed to the spatial heterodyne spectrometer (SHS). Figure S2a illustrates the schematic layout of the ADC (see also Figures 2e and S2b). The structure consists of two cores that gradually approach each other. The gap width decreases from g_0 = 500 nm to g_{ADC} = 150 nm over the coupling length of L_{ADC}. For the upper waveguide, the core width decreases from W_1 = 430 nm to W_0 = 400 nm, whereas for the lower waveguide, the core width increases from W_1 = 370 nm to W_0 = 400 nm. Thus, the input TE_0 mode will convert to the super symmetric (i.e., S) mode if L_{ADC} is long enough and the adiabatic condition is fulfilled. Two cores are then separated into ports #1 and #2 through a short Y-branch section with L_0 = 10 μm, thereby achieving 3-dB coupling. By using a short taper section with L_{tp} = 4.5 μm, the core width at each input and

output port becomes W_{wg} = 450 nm. We calculate the conversion efficiencies from the $\mathrm{TE_0}$ mode to the super symmetric and anti-symmetric (i.e., S and AS) modes, as shown in Figure 2f. The conversion efficiency of the S mode levels off to ≈ 1 at the coupling length of L_{ADC} = 150 μm, indicating that the adiabatic condition is met. Figure S2c shows the light propagation profile of the optimized ADC. A Mach-Zehnder interferometer (MZI) was fabricated to evaluate the device performance. Figure 2g shows the measured interference curve of the MZI, from which the coupling ratios ($|\kappa_{\mathrm{ADC}}|^2$) can be derived with the following formula:

$$\left|\kappa_{\mathrm{ADC}}\right|^2 = \frac{1}{2} \pm \frac{1}{2}\sqrt{\frac{1}{10^{\mathrm{ER}}}}, \tag{S6}$$

where ER denotes the extinction ratio of the coupling-ratio curve. In Figure 2h, we show the calculation and measurement results of $|\kappa_{\mathrm{ADC}}|^2$. Throughout the wavelength span from λ = 1.45 μm to 1.65 μm, the coupling ratio varies slightly around $|\kappa_{\mathrm{ADC}}|^2 \approx 0.5$, enabling the broadband operation of the tFTS. The ripples in the curve results from the noise in the measurement of weak signals. We calculate $|\kappa_{\mathrm{ADC}}|^2$ under fabrication flaws, such as core-width and gap-width deviations (denoted as ΔW and Δg), as shown in Figures S2d and S2e. It can be found that, even with ΔW = ±20 nm and Δg = ±20 nm, the calculated $|\kappa_{\mathrm{ADC}}|^2$ is still quite uniform. As a cross reference, some end-to-end cascading structures were also fabricated on the same chip (see Figure S2f). Here, we mainly consider the transmission at the port #1, while the port #2 was connected to a tapered waveguide to prevent reflection. The coupling ratio at the port #1 can be derived by averaging the difference in the transmittances of 1 and 9 cascading ADCs, as shown in Figure S2g. The measured $|\kappa_{\mathrm{ADC}}|^2$ agrees well with the result obtained from the MZI and has fewer disturbances, demonstrating that the ripples in Figure 2h result mainly from measurement inaccuracy.

Note 5. Design and characterization of the Y-branch splitter

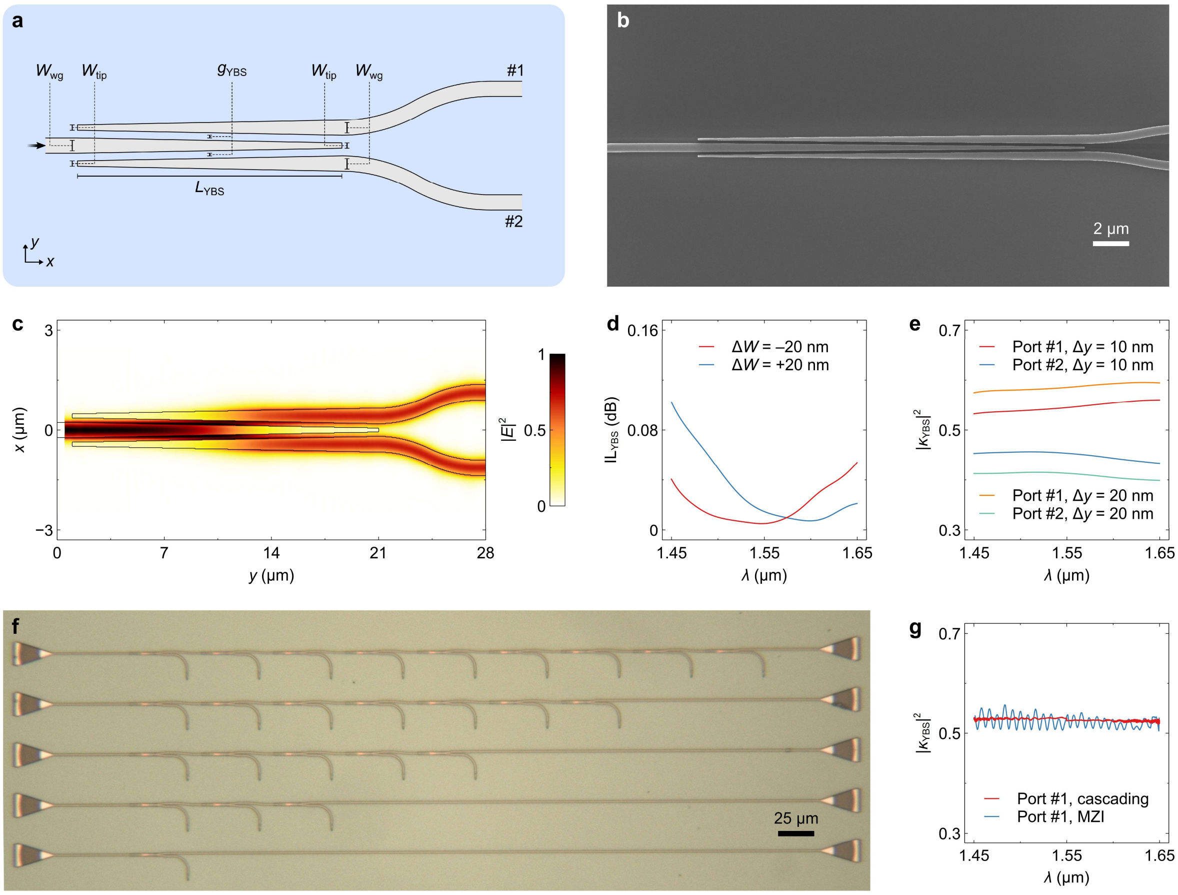

Figure S3 Y-branch splitter (YBS). (**a**) Schematic illustration of the YBS with parameters labeled. (**b**) Scanning electron microscope image (SEM) of the fabricated YBS. (**c**) Calculated light propagation profile. (**d**) Calculated insertion losses (IL_{YBS}) with varying core-width deviations (ΔW) at varying wavelengths (λ). (**e**) Calculated coupling ratios ($|\kappa_{YBS}|^2$) with varying central-core dislocations (Δy) at varying wavelengths (λ). (**f**) Microscope image of the fabricated cascading structures. (**g**) Measured $|\kappa_{YBS}|^2$ dispersions at the port #1. The result measured from the Mach-Zehnder interferometer (MZI) is also plotted for comparison. The excess losses are deducted from the presented spectra.

The Mach-Zehnder interferometer (MZI) in the spatial heterodyne spectrometer (SHS) is formed by linking two Y-branch splitters (YBS) with folded arms. The YBS consists of three cores that are closely spaced. Figure S3a illustrates the device (see also Figures 2i and S3b). The width of the central core decreases from W_{wg} = 450 nm to W_{tip} = 100 nm, whereas for two lateral cores, their widths increase from W_{tip} = 100 nm nm to W_{wg} = 450 nm. Thus, light launched from the central core will evenly couple to two lateral cores. Such a scheme outperforms other types of 1×2 splitters, e.g., the multimode interference coupler (MMI), in terms of lower losses and negligible reflection. In Figure 2j, we show the calculated conversion efficiencies as a function of the coupling length (L_{YBS}). The curve becomes flat when L_{YBS} > 20 μm, indicating sufficient adiabaticity. The calculated light propagation profile is shown in Figure S3c. A testing MZI was employed to characterize the

coupling ratios ($|\kappa_{YBS}|^2$) of the YBS (see the upper panel of Figure 2k). Form the $|\kappa_{YBS}|^2$ curve shown in Figure 2l, the fabricated YBS has a virtually balanced output and minimal losses. The tolerance analysis is given in Figures S3d and S3e. In Figure S3d, we show the calculated insertion losses (IL_{YBS}) under width deviations (ΔW) on three cores. It is possible to achieve low losses of $IL_{YBS} < 0.1$ dB, even with $\Delta W = \pm 20$ nm. The lateral dislocation (Δy) of the central core is an additional source of fabrication imperfection. Under the dislocation of $\Delta y = 20$ nm, the YBS transmission will deviate from ≈ 50:50 to ≈ 60:40. Nevertheless, since the three cores are densely packed, it is viable to obtain a high fabrication precision ($\Delta y < 5$ nm) to ensure a 3-dB coupling. The $|\kappa_{YBS}|^2$ curve measured in Figure 2l contains ripples with a uniform period of ≈ 9 nm, which could be the consequence of fabrication defects in one arm of the MZI (for explanations, see Figure S4). To validate the uniformity of $|\kappa_{YBS}|^2$, some testing structures with 1 ~ 9 cascading YBSs were fabricated, as shown in Figure S3f. The input port of each YBS was connected to the port #1 of the preceding YBS, while the port #2 was left unused and connected to a short taper. Thus, the transmission at the port #1 can be derived by averaging the transmittance difference between 1 and 9 YBSs. Figure S3g shows a comparison between the measured $|\kappa_{YBS}|^2$ curve and MZI result. The result measured with the cascading structures shows negligible variations, demonstrating that the ripples in Figure 2l are not caused by the YBS.

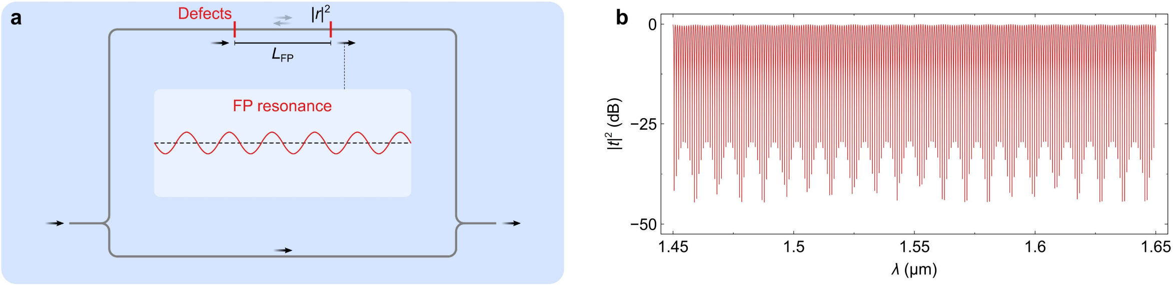

Figure S4 Explanation on ripples. (**a**) Illustration of a Mach-Zehnder interferometer (MZI) with a defect in one interference arm. (**b**) Calculated transmission ($|t|^2$) at the output. Some ripples can be found in the measured coupling ratios ($|\kappa_{YBS}|^2$) of the Y-branch splitter (YBS) shown in Figure 2l. The $|\kappa_{YBS}|^2$ curve is derived from the interference of a Mach-Zehnder interferometer. From the lower panel of Figure 2k, the extinction ratios (ER) of the MZI vary with a period of ≈ 9 nm. According to the tolerance analysis shown in Figures S3d and S3e, the deviations in YBS parameters cannot induce such a periodicity. In addition, the periodic variation in ERs is not due to the reflection at grating couplers, as the period would be much smaller if that were the case. We assume that the ripples may result from the fabrication defects in the interference arm. For instance, if there are two defects (e.g., sidewall discontinuity or air void) in one arm, the slight reflection (denoted as $|r|^2$) will induce a weak FP resonance that sinusoidally changes the interference contrast and ERs, as illustrated in Figure S4a. To verify this viewpoint, we give the calculated dispersion curves of the MZI under defects, as shown in Figure S4b. Here, the spacing between defects is set as $L_{FP} = 30$ μm and their reflectivity is set as $|r|^2 \approx 0.03$. Similar periodicity (≈ 9 nm) can be found from the curve. The occurrence of defects is fully stochastic and the measured transmittances of most MZIs in the SHS do not have noticeable ripples in their ERs (see Figures 3e and 4a). These defects may result from the stitching errors between writing fields during electron-beam lithography.

Note 6. Design and characterization of the spectrometer

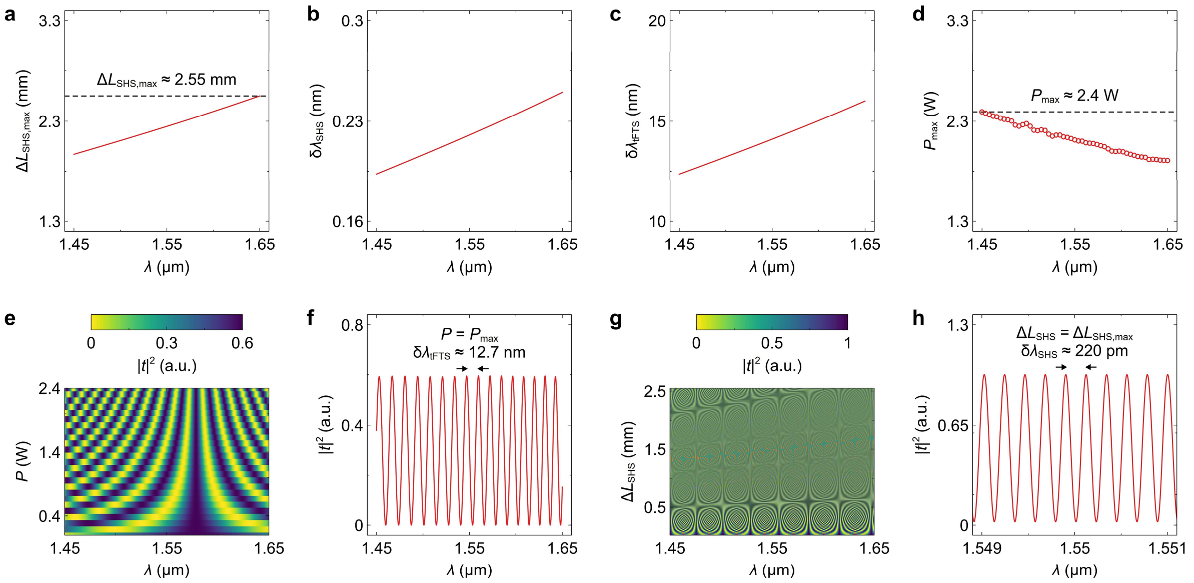

Figure S5 Design process. (**a**) Calculated maximum arm-length difference ($\Delta L_{\mathrm{SHS,max}}$) of the spatial heterodyne spectrometer (SHS) at varying wavelengths (λ). The dashed line represents $\Delta L_{\mathrm{SHS,max}} \approx 2.55$ mm. (**b**) Calculated resolution ($\delta\lambda_{\mathrm{SHS}}$) of the SHS at varying λ. (**c**) Calculated resolution ($\delta\lambda_{\mathrm{tFTS}}$) of the tunable Fourier-transform spectrometer (tFTS). (**d**) Calculated maximum heating power (P_{max}) at varying λ. The dashed line represents $P_{\mathrm{max}} \approx 2.4$ W. (**e**) Calculated transmittance ($|t|^2$) matrix of the tFTS. (**f**) Calculated $|t|^2$ of the tFTS at $P = P_{\mathrm{max}}$ and varying λ. Around $\lambda = 1.55$ μm, the tFTS resolution is calculated to be $\delta\lambda_{\mathrm{tFTS}} \approx 12.7$ nm. (**g**) Calculated $|t|^2$ matrix of the SHS. (**h**) Calculated $|t|^2$ of the SHS at $\Delta L_{\mathrm{SHS}} = \Delta L_{\mathrm{SHS,max}}$ and varying λ. Around $\lambda = 1.55$ μm, the SHS resolution is calculated to be $\delta\lambda_{\mathrm{SHS}} \approx$ 220 pm.

In this section, we first discuss the design flow of the two-dimensional Fourier-transform spectrometer (2D-FTS). The proposed 2D-FTS is realized by combining a tunable Fourier-transform spectrometer (tFTS) and a spatial heterodyne spectrometer (SHS), as shown in Figures 1a and 1c. The resolution ($\delta\lambda_{\mathrm{f}}$) of the 2D-FTS is determined by the resolution ($\delta\lambda_{\mathrm{SHS}}$) of the SHS at the Rayleigh criterion[S50]:

$$\delta\lambda_{\mathrm{f}} = \delta\lambda_{\mathrm{SHS}} = \frac{\lambda^2}{n_{\mathrm{g}}\Delta L_{\mathrm{SHS,max}}}, \tag{S7}$$

where λ denotes the wavelength, n_{g} denotes the group index, and $\Delta L_{\mathrm{SHS,max}}$ denotes the maximum arm-length difference of the SHS. Here, $\delta\lambda_{\mathrm{f}}$ only depicts the resolution capability defined by the fundamental frequency of samplings, as will be discussed later. A uniform resolution grid is adopted in the spectrum reconstruction. From Equation S7, however, $\delta\lambda_{\mathrm{f}}$ will vary with λ, given a fixed $\Delta L_{\mathrm{SHS,max}}$. We calculate the required $\Delta L_{\mathrm{SHS,max}}$ to attain $\delta\lambda_{\mathrm{SHS}} = 250$ pm at varying λ, as shown in Figure S5a. The maximum arm-length difference is set as the maximum value (i.e., $\Delta L_{\mathrm{SHS,max}} = 2.55$ mm), in order to ensure that the target resolution ($\delta\lambda_{\mathrm{SHS}} < 250$ pm) can be attained at every single wavelength grid, as shown in Figure S5b. For the 2D-FTS, it is essential to ensure

that the resolution ($\delta\lambda_{tFTS}$) of the tFTS is finer than the free spectral range (FSR_{SHS}) of the SHS, which yields:

$$\delta\lambda_{tFTS} < FSR_{SHS} = \frac{N_{SHS}\delta\lambda_{SHS}}{2}, \tag{S8}$$

where N_{SHS} (=128) denotes the number of the Mach-Zehnder interferometers (MZI) in the SHS. Owing to the dispersion of $\delta\lambda_{SHS}$, the critical $\delta\lambda_{tFTS}$ required to meet Equation S8 is also dispersive, as shown in Figure S5c. We calculate the maximum heating power (P_{max}) required at varying λ using Equations S4, S8, and the tuning efficiency shown in Figure 2d. The calculation result can be found in Figure S5d. The heating power is thus determined as P_{max} = 2.4 W, so that Equation S8 can be fulfilled over the whole wavelength span. The number of power sweep steps can be determined using the formula below:

$$N_{tFTS} = \frac{2 \cdot BW}{\delta\lambda_{tFTS}}, \tag{S9}$$

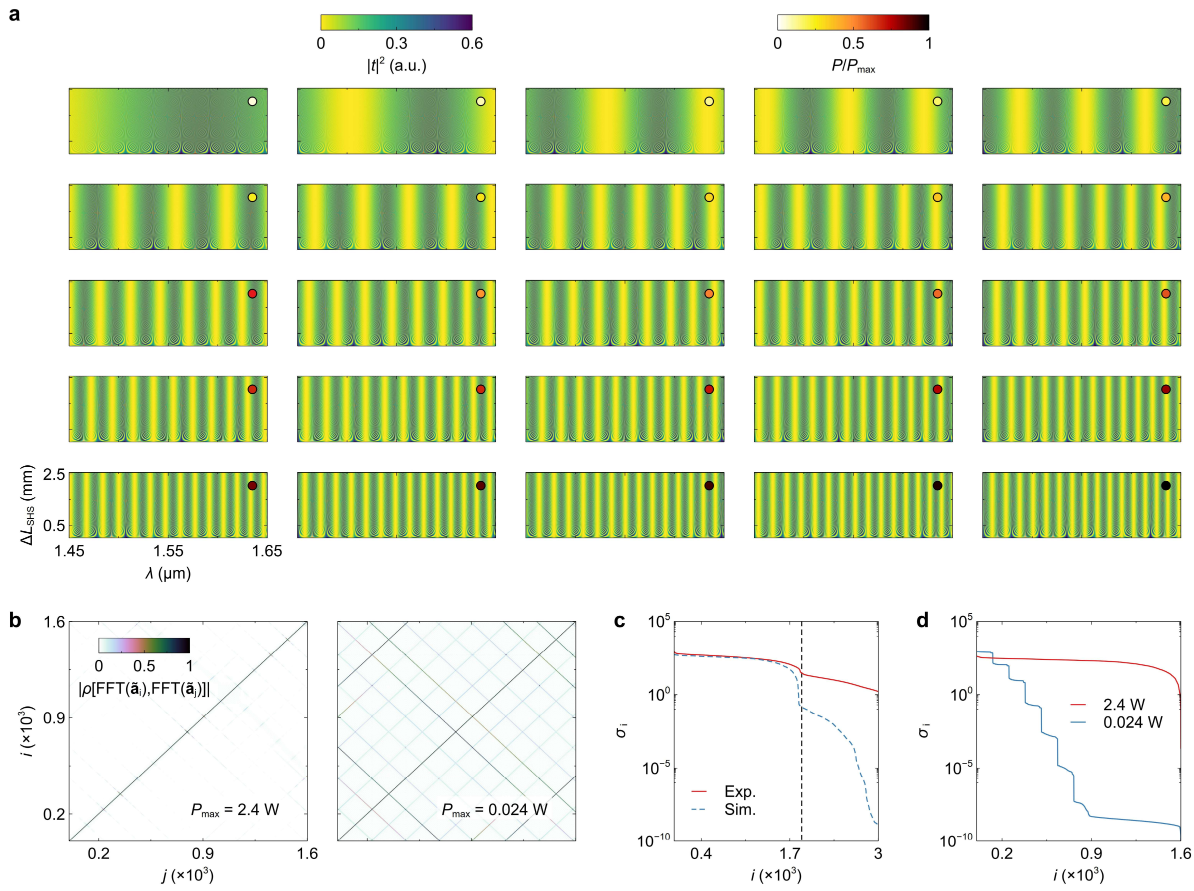

Figure S6 Two-dimensional Fourier-transform spectrometer (2D-FTS). (**a**) Calculated transmittance ($|t|^2$) cube. Here, the cube is sliced into a series of matrices with varying heating power (P), represented by the colors of dots in the upper right corner. (**b**) Calculated correlation matrices with the maximum heating power of P_{max} = 2.4 W and 0.024 W. Here, the correlation operation [$\rho(\cdot,\cdot)$] is performed on the fast Fourier transform (FFT) of the fringe vectors ($\tilde{\mathbf{a}}_i$) with zero-frequency components removed. (**c**) Singular values (σ_i) derived from the over-sampled $|t|^2$ cube. The measurement result is also plotted for comparison. The dashed line represents the location of the kink. (**d**) Calculated σ_i with P_{max} = 2.4 W and 0.024 W.

where BW (=200 nm) denotes the operation bandwidth of the 2D-FTS. From Figure S5c, $\delta\lambda_{tFTS}$ increases at a longer wavelength. From the minimum resolution at λ = 1.45 μm, we derive the critical number of sweep steps as N_{tFTS} = 32. Notably, due to the unique folding property of 2D Fourier map, it is feasible to reduce the sweep steps to < 2·BW/$\delta\lambda_{tFTS}$ while maintaining sufficient decorrelation and a high reconstruction accuracy, as will be discussed later. In this work, the number of power sweep steps is optimized to be N_{tFTS} = 25. The calculated transmittance ($|t|^2$) matrices of the tFTS and SHS are shown in Figures S5e and S5g, respectively. Figures S5f and S5h respectively show the calculated $|t|^2$ of the tFTS and SHS at $P = P_{max}$ and $\Delta L_{SHS} = \Delta L_{SHS,max}$. For clarity, the excess loss of the 1×128 power splitter (PS) has been deducted from the result.

We derive the transmittance ($|t|^2$) cube of the 2D-FTS using the aforementioned results (see Figure S6a). Here, the cube is presented as the matrices at N_{tFTS} (= 25) power sweep steps. For clarity, the excess loss of the PS has been deducted. All matrices are channelized into N_{2f} = 1601 columns. In Equation S7, we give the definition of $\delta\lambda_f$ based on the fundamental frequency of samplings. The corresponding channel capacity can be derived as $N_f = BW/\delta\lambda_f + 1$ = 801, which is half of N_{2f}. The definitions of $\delta\lambda_f$ and N_f reflect the fact that the discrimination of two discrete spectral lines necessitates an extra wavelength point between them. However, these additional points also carry information, especially when computational methods are used, as will be demonstrated by matrix analysis. Furthermore, according to the Nyquist's theorem[S51], a sinusoidal response must be sampled at more than twice the highest frequency; otherwise, the sampling at $\Delta L_{SHS} = \Delta L_{SHS,max}$ will be ineffective (see Figure S7a). Hence, we propose to use the modified definitions of resolution and capacity: $\delta\lambda_{2f} = \delta\lambda_f/2$ = 125 pm, $N_{2f} = 2N_f$ = 1601, which are more consistent with the matrix structure. These two sets of definitions are not contradictory, but rather depict the capability of spectrum reconstruction in different manners.

Before further analysis, the 3D cube is reorganized into a 2D matrix (denoted as $\mathbf{A}$) with N_{2f} (= 1601) columns and $N_{tFTS}\times N_{SHS}$ (= 3200) rows. Each column is a vector (denoted as $\mathbf{a}_i$) that is flattened from the 2D fringe with N_{tFTS} (=25) columns and N_{SHS} (= 128) rows. To be specific, the element of $\mathbf{A}$ at the i-th column and $(j-1)\times N_{tFTS} + k$-th row represents the transmittance at the i-th wavelength channel, j-th power sweep step, and k-th output channel. Figure S6b shows the correlation matrix $[\rho(\cdot,\cdot)]$ derived from $\tilde{\mathbf{A}}_{FFT}$. Here, each column of $\tilde{\mathbf{A}}_{FFT}$ is the Fast Fourier transform (FFT) of $\mathbf{a}_i$ with zero-frequency components removed (denoted as $\tilde{\mathbf{a}}_i$). The removal operation will be explained later. In the correlation matrix, the element at the i-th column and j-th row is the Pearson correlation of FFT($\tilde{\mathbf{a}}_i$) and FFT($\tilde{\mathbf{a}}_j$). The correlation matrix is quasi-diagonal when the heating power reaches the critical value of P_{max} = 2.4 W, demonstrating that all the wavelength channels

are highly decorrelated. In contrast, with a lower heating power of P_{max} = 0.024 W, more near-unity elements appear in the correlation matrix, indicating that, at the same output port but distinct sweep steps, the fringe patterns are highly similar, which restricts the bandwidth within a single FSR_{SHS}. The correlation matrix only depicts the linear independence between each pair of column vectors, and it is necessary to use the singular-value decomposition (SVD) to verify the orthogonality of the full space. We calculate the singular values (σ_i) of $\tilde{\mathbf{A}}_{FFT}$. Here, $\tilde{\mathbf{A}}_{FFT}$ is oversampled into > 3000 wavelength channels. A kink can be found at $i \approx 1900$, which exceeds N_{2f} (=1601). This confirms that, in the computational reconstruction, the Rayleigh criterion can be overcome, and the channel capacity can be doubled. The kink location is slightly beyond the prediction since $\delta\lambda_{SHS}$ is finer at shorter wavelengths. The measurement result is also plotted in Figure S6c, which is in good accord with simulations. In Figure S6d, we show the calculated σ_i curve with P_{max} = 2.4 W and 0.024 W. The σ_i curve is flat and smooth with P_{max} = 2.4 W, demonstrating that all N_{2f} (=1601) channels are effective. With a weak heating (P_{max} = 0.024 W), the σ_i curve is cut off by a series of kinks and drops rapidly to near zero (< 10^{-10}), indicating that the decorrelation is insufficient, and a large portion of spectral information is entirely lost. The corresponding measurement results can be found in Figure 3e, 4b, and 4c.

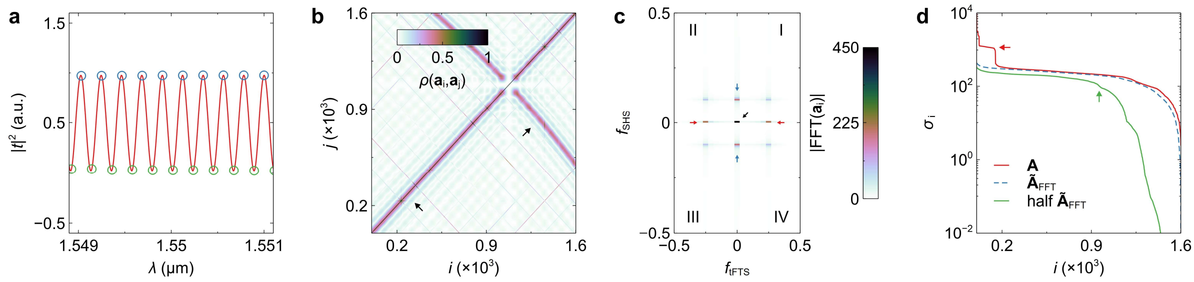

Figure S7 Further matrix analysis. (**a**) Calculated transmittance ($|t|^2$) of the spatial heterodyne spectrometer (SHS) at $\Delta L_{SHS} = \Delta L_{SHS,max}$ and varying wavelengths (λ). The circles represent the sampling points at twice the fundamental frequency. (**b**) Calculated correlation matrix [$\rho(\cdot, \cdot)$] derived from the original calibration matrix (**A**). The arrows highlight the highly correlated non-diagonal elements. (**c**) Calculated two-dimensional Fast Fourier transform (FFT) of the fringe at λ = 1.51 μm. The red arrows highlight the projections at f_{SHS} = 0, whereas the blue arrows highlight the projections at f_{tFTS} = 0. The black arrow shows the DC component at $f_{SHS} = f_{tFTS}$ = 0. (**d**) Singular values (σ_i) derived from **A**, FFT result ($\tilde{\mathbf{A}}_{FFT}$) with zero-frequency components removed, and half $\tilde{\mathbf{A}}_{FFT}$. The red arrow highlights the kink induced by the projection effect. The green arrow highlights the location where the decay rate rapidly increases for the half $\tilde{\mathbf{A}}_{FFT}$.

Next, we will address some issues that have not been thoroughly discussed. First, we provide additional discussions on the channelization of **A**. We calculate the response of the SHS at $\Delta L_{SHS} = \Delta L_{SHS,max}$, as shown in Figure S7a. The blue circles represent the sampling points arranged with the resolution grid of $\delta\lambda_f$. In this instance, the SHS response is sampled only once within each period since $\delta\lambda_f$ is defined as the peak-to-peak spacing of a sinusoidal curve. As a result, the recorded signal (**O**) at such a sampling will not contain effective

information. According to the Nyquist's theorem, it is essential to double the frequency ($\delta\lambda_{2f} = \delta\lambda_f/2$) to have at least two sampling points within each period (see the blue and green circles in Figure S7a).

Second, we explain the projection effect in the 2D-FTS. In Figure S7b, we show the correlation matrix derived from the original **A**. Apart from the self-correlated diagonal elements, the correlation matrix exhibits additional high-correlation "shades". The origin of this phenomenon can be revealed in the Fourier domain. Figure S7c shows the FFT result of the fringe at λ = 1.51 μm, as an example. The FFT map is divided into four quadrants, each of which contains a single spot that indicates the intensity information at this wavelength. When the Fourier map is unfolded along f_{tFTS} (see Figure S8), the locations of spots will be distinct at varying λ, as discussed in Figure 1d. In spite of this, each spot has four additional projections on the two axes (f_{SHS} = 0 and f_{tFTS} = 0, see the arrows). For instance, the spot projections of two fringes with distinct f_{tFTS} but identical f_{SHS} will overlap at f_{tFTS} = 0, which degrades orthogonality. This problem can be resolved by omitting the zero-frequency components of $\mathbf{a}_i$ and **O** and using numerical decomposition to reconstruct a spectrum (see Note 8 for more details), provided that FFT is a linear transform. The origin of correlations is more complex when N_{tFTS} is reduced to < 2·BW/$\delta\lambda_{tFTS}$, as will be discussed later. To verify the effectiveness of this operation, we calculate the σ_i curves of **A** and $\tilde{\mathbf{A}}_{FFT}$ (see Figure S7d). The kink in the σ_i curve is eliminated after the removal of zero-frequency components (see the red arrow). $\tilde{\mathbf{A}}_{FFT}$ is a complex matrix with N_{2f} (= 1601) columns and (N_{tFTS} – 1)×(N_{SHS} – 1) (= 3048) rows, corresponding to an over-determined inverse problem. In Figure S7d, we also calculate the σ_i curve with $\tilde{\mathbf{A}}_{FFT}$ truncated into a 1601×1601 matrix. The decay of σ_i rapidly increases at $i \approx 900$ (see the green arrow), indicating information losses. Thus, it is essential to use the full $\tilde{\mathbf{A}}_{FFT}$ without truncation.

Third, we discuss the feasibility of reducing N_{tFTS} and its impact on correlations. The critical number of power sweep steps is N_{tFTS} = 2·BW/$\delta\lambda_{tFTS}$. When N_{tFTS} exceeds this critical value, the Fourier map will be fully unfolded, as shown in Figure S8b. As the wavelength increases, the "vertical" location of a spot in the Fourier map oscillates between f_{SHS} = 0 and 1/2. In the meantime, its "lateral" location shifts from f_{tFTS} = 1/2 to 0. Here, the direction of spot shift is determined by the initial phase of the 2D-FTS at the first sampling. When fewer sweep steps (i.e., N_{tFTS} < 2·BW/$\delta\lambda_{tFTS}$) are utilized, the Fourier map will become folded, and a spot will undergo an additional backtracking. To be specific, the spot shifts from f_{tFTS} = 1/2 to 0 when the wavelength ranges from minimum (λ_{min}) to medium (λ_{mid}), and then rebounds from f_{tFTS} = 0 for the remaining bandwidth (i.e., from λ_{mid} to λ_{max}). Consequently, the Fourier map contains two zig-zag trajectories, as shown in Figure S8c. Due to the "mirror flip" at f_{tFTS} = 0, the forward and backward trajectories are inverted and dislocated,

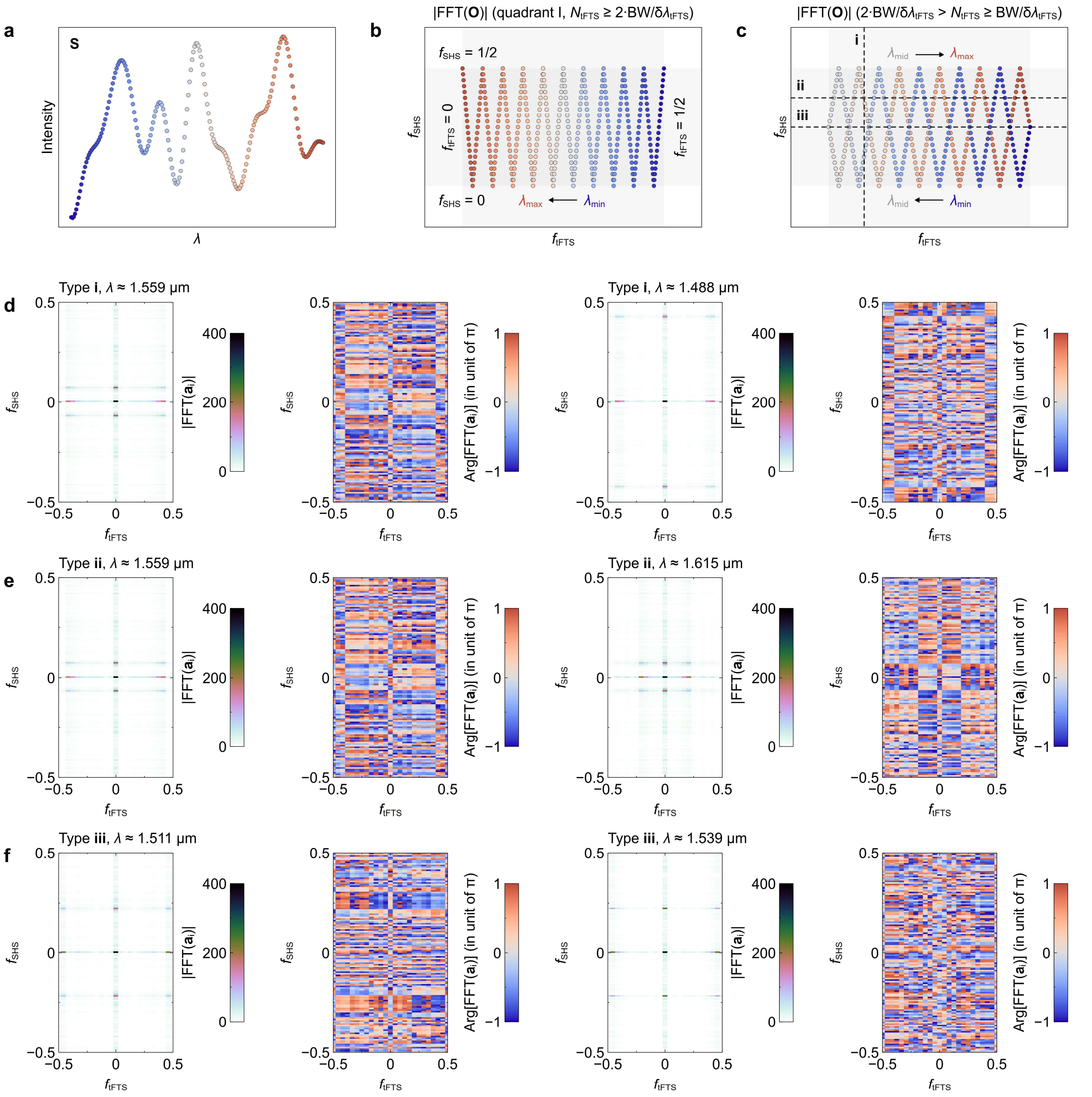

Figure S8 Folding of the Fourier map. Illustrations of an (**a**) arbitrary input spectrum and the (**b**-**c**) fast Fourier transform (FFT) of output interferograms with different power sweep steps (N_{tFTS}). When the number of sweep steps is chosen as $N_{tFTS} = 2 \cdot BW/\delta\lambda_{tFTS}$, the locations of spots are fully unfolded along f_{tFTS}, allowing for an ideal point-to-point mapping. The reduction of sweep steps will lead to two dislocated trajectories in a folded Fourier map. The similarity between fringes can be categorized into three cases (i.e., **i**-**iii**). (**d**-**f**) Intensity and phase maps of FFT results at different wavelengths (λ) derived from the measured transmittance cube.

making it possible to obtain decorrelation between the folded spots with similar f_{tFTS}. Such a phenomenon is unique to 2D-FTSs. For 1D-FTSs, any folding of a 1D Fourier map will cause unsolvable obscurity between channels. Next, we will demonstrate the feasibility of reconstructing spectra from a folded 2D Fourier map with a reduced N_{tFTS}. From Figure S8c, the origin of inter-channel correlations is categorized into three cases:

(**i**) Two spots have similar f_{tFTS} but different f_{SHS};

(**ii**) Two spots have similar f_{SHS} but different f_{tFTS};

(**iii**) Two spots locate closely at the crossover of two trajectories.

Some examples are provided in Figures S8d-S8f. For instance, at the wavelengths of $\lambda \approx 1.559$ µm and 1.488 µm, the residual correlations mainly result from their similar projections on the f_{tFTS} axis. In the second case,

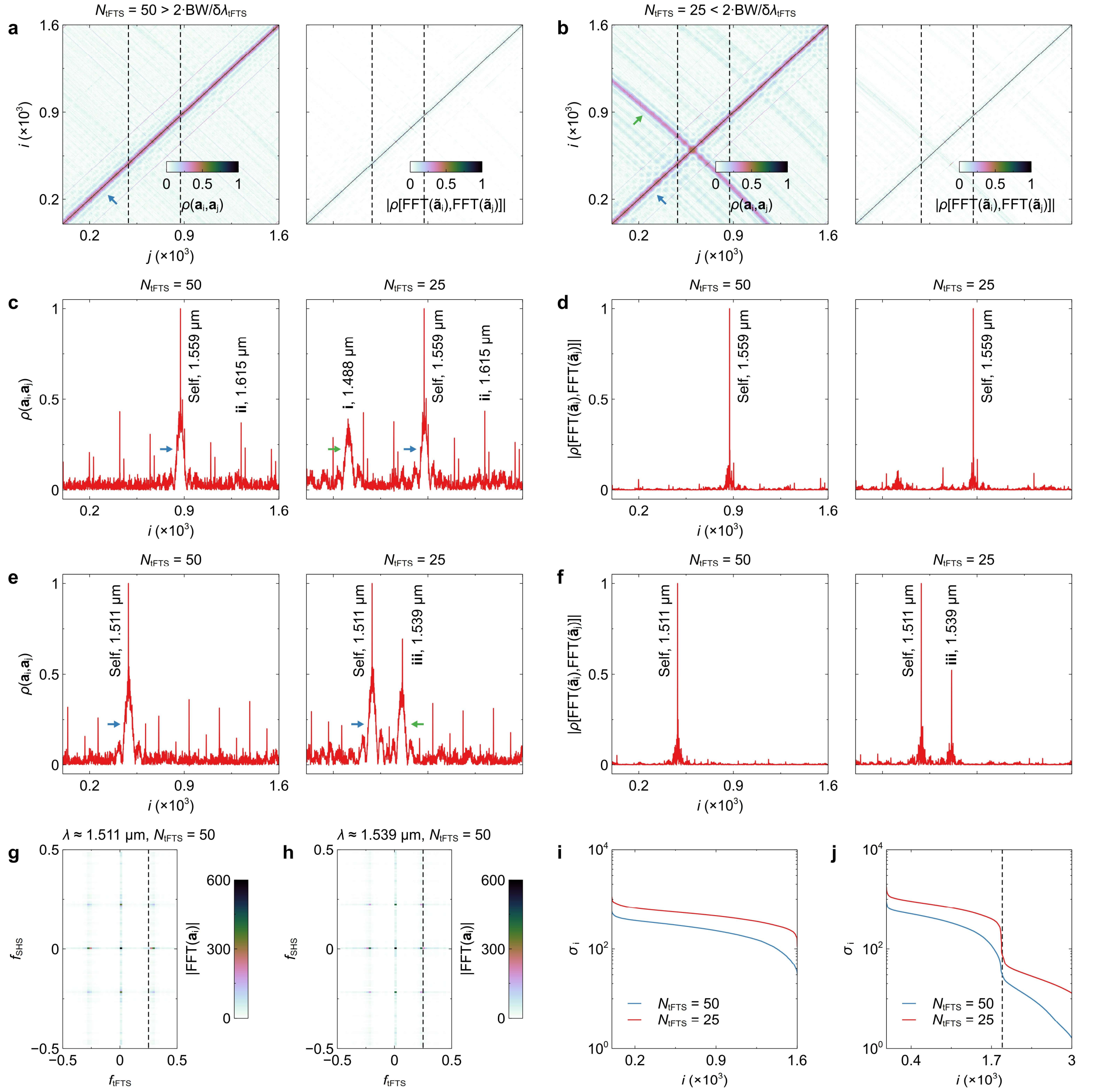

Figure S9 Correlation analysis. Correlation matrices derived from the measurement results with power sweep steps of (**a**) $N_{\mathrm{tTFS}} = 25$ and (**b**) $N_{\mathrm{tTFS}} = 50$. The left panels show the correlations [$\rho(\cdot,\cdot)$] between the fringes ($\mathbf{a}_i$) at different wavelengths. The right panels show the correlations of the fast Fourier transform (FFT) of fringes ($\tilde{\mathbf{a}}_i$) with zero-frequency components omitted. The green and blue arrows indicate the high-correlation elements with $i \approx j$ and $i + j \approx Const$, respectively Here, i and j denote channel indices, and *Const* is a constant related to the channel index at $f_{\mathrm{tFTS}} = 0$. The dashed lines represent two slices of the matrix at $\lambda \approx 1.559$ µm and 1.511 µm. Correlations between the fringes at $\lambda \approx 1.559$ µm and other wavelengths (**c**) before and (**d**) after the component removal. Correlations between the fringes at $\lambda \approx 1.511$µm and other wavelengths (**e**) before and (**f**) after the component removal. The left and right panels show the results with $N_{\mathrm{tTFS}} = 25$ and 50, respectively. Intensity maps of FFT results at (**g**) $\lambda \approx 1.511$ µm and (**h**) $\lambda \approx 1.539$ µm with $N_{\mathrm{tTFS}} = 50$. The dashed line represents $f_{\mathrm{tFTS}} = 0.25$. (**i**) Singular values (σ_i) derived from the measured transmittance cubes with $N_{\mathrm{tTFS}} = 25$ and 50. (**j**) σ_i curves derived from the oversampled cubes. The dashed line represents the location of the kink.

the channels at $\lambda \approx 1.559$ μm and 1.615 μm have similar projections on the f_{SHS} axis. Figure S8f shows two spots at the crossover point with both similar f_{tFTS} and f_{SHS} (i.e., type **iii**). Hence, it is challenging to directly read out a spectrum from the DCT map with $N_{tFTS} < 2 \cdot BW/\delta\lambda_{tFTS}$, as some spots are closely located. Next, we will give a rigorous analysis on the correlation in a folded 2D Fourier map and show the feasibility of solving this issue with the proposed numerical method.

Figures S9a and S9b show the correlation matrices derived from the measurement results with N_{tTFS} = 25 (< $2 \cdot BW/\delta\lambda_{tFTS}$) and 50 (> $2 \cdot BW/\delta\lambda_{tFTS}$), respectively. In each plot, we compare the correlation properties before and after the removal of zero-frequency components. With $N_{tFTS} = 50$, the original correlation matrix contains a shade in the vicinity of diagonal elements ($i \approx j$, see the blue arrow), which is caused by the overlap of projections of adjacent spots on the f_{tFTS} axis in the Fourier map. In addition, a "noisy" background can be found over the non-diagonal region, which results from the similar projections of different spots on the f_{SHS} axis in the Fourier map (type **ii** in Figure S8c). Since both cases result from the projection effect, correlations can be inhibited by omitting the zero-frequency components. After the component removal, the correlation matrix becomes diagonal, as shown in the right panel of Figure S9a. If the sweep steps are decreased to N_{tFTS} = 25, an additional shade will appear in the correlation matrix (see the green arrow in Figure S9b). Such a shade is caused by the similar projections of folded trajectories on the f_{tFTS} axis in the Fourier map (type **i** in Figure S8c). This is evidenced by the fact that each element in this shade has a virtually constant sum of row and column indices, i.e., $i + j = Const$, corresponding to the folding point at a medium wavelength. These high-correlation elements can also be eliminated via component removal, as shown in the right panel of Figure S9b. However, some correlations still remain in the cube after the removal. For clarity, the correlation matrix is sliced at two typical wavelengths. The right panel of Figure S9c shows the correlations between $\lambda \approx 1.559$ μm and other wavelengths with $N_{tFTS} = 25$. The near-unity peak is associated with the self-correlation. The bump around the self-correlated peak (see the blue arrow) relates to the shade with $i \approx j$. The bump at $\lambda \approx$ 1.488 μm (see the green arrow) is induced by the type-**i** correlation. Other peaks (e.g., $\lambda \approx 1.615$ μm) are tied to the type-**ii** correlation. The linewidths of bumps are wider than peaks due to the "slower" shift along f_{tFTS} in the Fourier map. For most wavelengths, all the superfluous bumps and peaks can be eradicated after the component removal, as shown in the right panel of Figure S9d. However, the removal operation is applicable solely in cases **i** and **ii**. In the right panel of Figure S9e, we show the correlation function sliced at $\lambda \approx 1.511$ μm. The peak resides on a bump at $\lambda \approx 1.539$ μm (see the green arrow), suggesting that this wavelength is mapped to a crossover in the Fourier domain (type **iii** in Figure S8c). Such a high correlation cannot be fully

depressed by extracting the zero-frequency components (see the right panel of Figure S9f). As discussed in Figure S8b, the type-**iii** correlation issue can be addressed by increasing the number of sweep steps. The left panel of Figure S9f shows the correlation function sliced at $\lambda \approx 1.511$ µm with $N_{\mathrm{tFTS}} = 50$. By comparison, the peak at $\lambda \approx 1.539$ µm diminishes, leaving only the self-correlated peak at $\lambda \approx 1.511$ µm. In Figures S9g and S9h, we show the calculated Fourier maps at $\lambda \approx 1.511$ µm and 1.539 µm. Compared to the results shown in Figure S8f, two spots are laterally shifted to $f_{\mathrm{tFTS}} < 0.25$ and > 0.25. It should be noted that, with $N_{\mathrm{tFTS}} = 25$, the majority of correlations are sufficiently inhibited and there are merely ≈ 5 ($< N_{\mathrm{tFTS}}/2$) pairs of channels suffering from the crossover problem. Moreover, even at a crossover, the residual correlation is still limited to $\rho(\tilde{\mathbf{a}}_i, \tilde{\mathbf{a}}_j) \approx 0.5$ after component removal, as shown in the right panel of Figure S9f. This is the result of two factors: first, due to spectral leakage (see Note 8), each channel occupies multiple rather than a single pixel in the Fourier map, and two spots do not precisely coincide at a crossover point; and second, the difference in phase distributions of FFT results also contributes to the improvement of decorrelations (see the 2nd and 4th columns of Figure S8f), as both intensity and phase information are useful in numerical reconstruction. To assess the solvability, we calculate the singular values (σ_i) of the measured cubes with $N_{\mathrm{tFTS}} = 25$ and 50, as shown in Figure S9i. The decay rates of σ_i curves are nearly identical, proving that reducing N_{tFTS} will not cause notable degradation in decorrelation. We also calculate the σ_i curves when the cubes are oversampled into > 3000 channels. The locations of kinks do not change, which is a direct proof that the channel capacity is not affected. From above analysis, it is conclusively demonstrated that sufficient decorrelation has been established at $N_{\mathrm{tFTS}} = 25$ and that all fringes can be numerically identified. It is viable to further reduce N_{tFTS} to support an even shorter sampling period, as long as the Fourier map is folded only once (see Figure S8c) and the following condition is met:

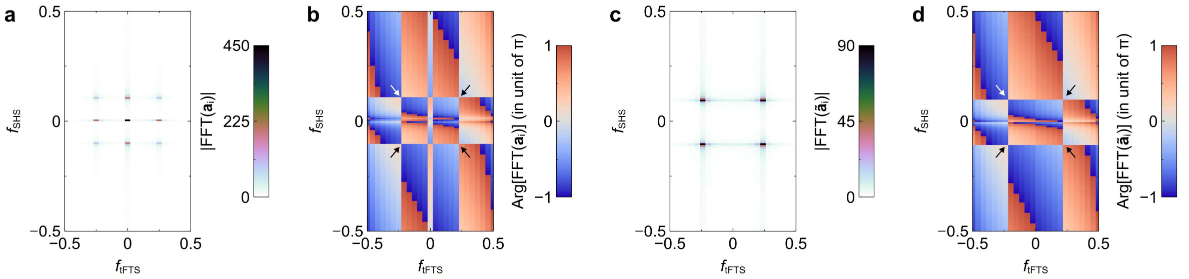

Figure S10 Component removal. (**a**) Intensity and (**b**) phase distributions of the fast Fourier transform (FFT) result before removing zero-frequency components. (**c**) Intensity and (**d**) phase distributions of the FFT result after the component removal. The arrows show phase hopping in the FFT maps.

$$\frac{2\cdot\mathrm{BW}}{\delta\lambda_{\mathrm{tFTS}}} > N_{\mathrm{tFTS}} \geq \frac{\mathrm{BW}}{\delta\lambda_{\mathrm{tFTS}}}. \tag{S10}$$

In this work, we choose to use N_{tFTS} = 25 to leave a margin and improve robustness, as the selected value is already quite small. When scaling to a higher dimension, the type-**iii** correlation can also be fully eliminated since a high-dimensional space provides a higher degree of freedom in arranging wavelength channels and circumventing crossover between spots. This issue will be discussed in Note 10.

Fourth, we give an example of component removal. The original intensity and phase distributions of the FFT results are shown in Figures S10a and S10b. Figures S10c and S10d show the FFT maps after omitting the zero-frequency components. The elements at f_{SHS} = 0 and f_{tFTS} = 0 are deleted, leaving only information within each quadrant. Some fringes may have their main Fourier components locating at f_{SHS} = 0 or f_{tFTS} = 0. In this case, a portion of effective information will be removed together with spot projections. Nevertheless, it is still feasible to recover these channels since a spot typically occupies multiple pixels in the Fourier map (see Figure S10c) and the majority of spectral information is still preserved. Remarkably, for a single-peak input, the phase distribution of the FFT map exhibits a hopping in each quadrant (see the arrows in Figures S10b and S10d), and the hopping location is precisely the spot location in the intensity map. Such a phase hopping is less visible in the experimental results (see Figure S4g for instance) due to the presence of noises.

Note 7. Analysis of temperature sensitivity

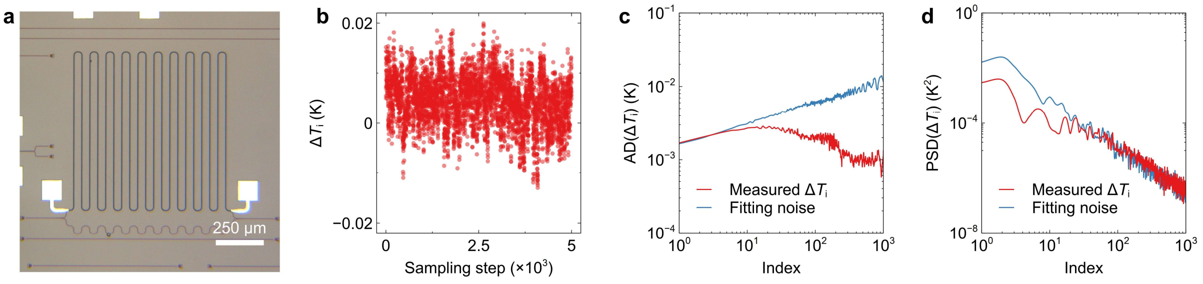

Figure S11 Temperature fluctuations. (**a**) Microscope image of the fabricated temperature sensor. (**b**) Measured time sequence of temperature fluctuations (ΔT_i). (**c**) Allan deviations (AD) of the measured ΔT_i and a generated fitting noise. (**d**) Welch power spectral densities (PSD) of the measured ΔT_i and a generated fitting noise.

The reconstruction accuracy is closely associated with the presence of noises in the measurement system. For the proposed device, the dominant source of noise is the temperature fluctuation, which is attributed to the high thermo-optical coefficient of silicon. An integrated temperature sensor was fabricated on the same die to capture the variation of the ambient temperature, as shown in Figure S11a. The structure is based on a Mach-Zehnder interferometer (MZI) with a large arm-length difference of 1.5 cm. We measured the output optical power at a fixed wavelength to extract the temperature drifts (ΔT_i), as shown in Figure S11b. The ΔT_i sequence has two critical attributes: the standard deviation (SD) and a frequency-dependent parameter (β). The definition of β can be found in Ref. S52. Figures S11c and S11d show the Allan deviation (AD) and Welch power spectral density (PSD) derived from the recorded ΔT_i. The definitions of the AD and PSD can be found in Refs. S53,S54. By fitting with a generated noise, the parameters are derived as SD(ΔT_i) ≈ 0.015 K and $\beta \approx -3.65$. The noise properties depend on the feedback response of the thermo-electric cooler (TEC) utilized in the experiment. The results shown in Figure S11c and S11d apply only for the noise in interferograms since β is related to the sampling frequency. For matrix calibration, the parameter is estimated to be $\beta \approx -2.5$ using the same method. Here, we assume that all interferograms are consequentially captured.

The estimated ΔT_i will be used in the numerical test to emulate the real-world environment (see Note 8, Figure S15). The measured interferogram (**O**) and matrix (**A**) can be expressed as:

$$\mathbf{O} = \left(\mathbf{A}_{\text{ext}} + \Delta\mathbf{A}\right)\mathbf{S}, \tag{S11}$$

$$\mathbf{A} = \mathbf{A}_{\text{ext}} + \Delta\mathbf{A}', \tag{S12}$$

where $\mathbf{A}_{\text{ext}}$ denotes the noiseless matrix, $\Delta\mathbf{A}$ denotes the deviation caused by ΔT_i, and **S** denotes the unknown spectrum. $\Delta\mathbf{A}$ can be derived from ΔT_i using the calculated temperature sensitivity (see Note 3), as has been

detailed in our previous studies[S12,S13]. From the numerical results shown in Figure S15, it is evident that the proposed spectrometer is robust against temperature fluctuations, and that it is possible to accomplish high accuracy under the current experimental conditions. In the experiment, the influence of ΔT_i is more complex than in the modeling. When a high heating power is applied to the tunable Fourier-transform spectrometer, for instance, the heat transfer will impose temperature drifts on the spatial heterodyne spectrometer. Also, due to the slower heat dissipation, the response time of the TEC will become longer at a high heating power. Nevertheless, all these effects can be depicted by a calibrated transmittance cube and are solvable based on the computational method (see Note 8).

Note 8. Reconstruction method

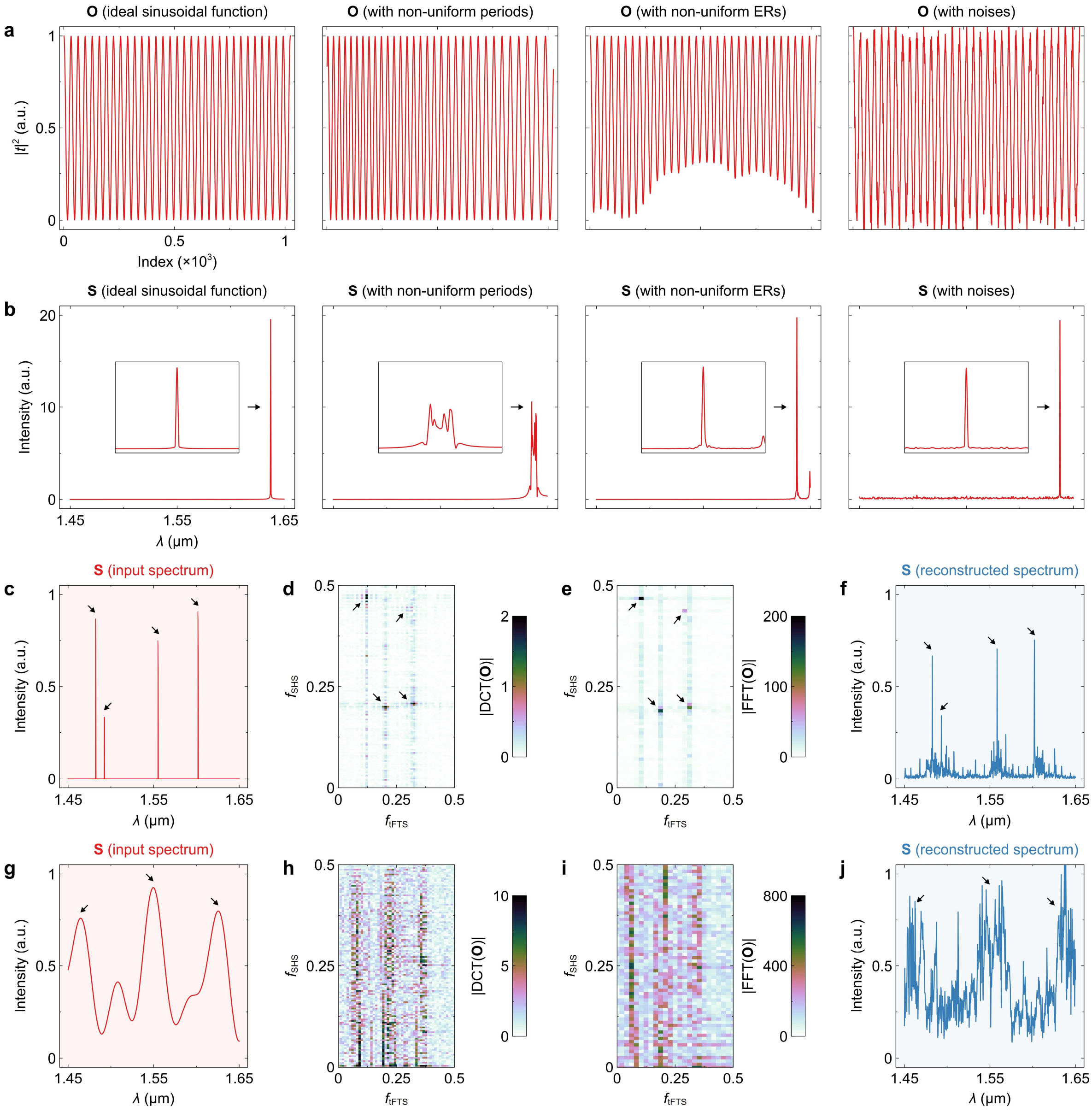

Figure S12 Reconstruction with discrete cosine transform (DCT). (**a**) 1D interferograms (denoted as **O**) with an ideal sinusoidal response, non-uniform periods, non-uniform extinction ratios (ER), and background noises. (**b**) Spectra (denoted as **S**) reconstructed by 1D-DCT. The insets show the enlarged views around the spike. (**c**) Testing spectrum with discrete features. (**d**) 2D-DCT and (**e**) fast Fourier transform (FFT) of the interferogram. (**f**) Spectra reconstructed by 2D-DCT. The arrows highlight the correspondence of spikes in the input/reconstructed spectra and the spots in the DCT/FFT map. (**g**) Testing spectrum with continuous features. (**h**) 2D-DCT and (**i**) FFT of the interferogram. (**j**) Spectra reconstructed by 2D-DCT. The arrows highlight the correspondence of bumps in the input and reconstructed spectra.

There are three prevalent approaches to spectrum reconstruction in Fourier-transform spectrometry (FTS): discrete cosine transform (DCT), pseudo inverse, and regularized iterative optimization. For FTSs with ideal sinusoidal responses, any arbitrary spectrum can be recovered from the DCT of the recorded interferogram.

However, a real-world interferogram typically has deviations from its ideal form, especially for integrated FTSs with strong dispersion and imperfect light extinction. For clarity, we first consider a simplified 1D-FTS model. Given an input spectrum (denoted as **S**) with a single spike, the output interferogram (denoted as **O**) is sinusoidal-like, as shown in the 1st column of Figure S12a. Here, the number of sampling steps is set as 2^{10} = 1024, while the period of **O** is set as 2^6 = 64, as an example. In the ideal case, the spectrum can be accurately recovered, as shown in the 1st column of Figure S12b. According to Equation S7, however, the free spectral range (FSR) of an MZI is wavelength-dependent, thereby resulting in non-uniform periods in interferograms, as shown in the 2nd column of Figure S12a. The chirp in **O** will broaden and split the retrieved spike, due to spectral leakage[S55] (see the 2nd column of Figure S12b). To be specific, DCT is the discrete Fourier transform (DFT) of the continuation of a finite sequence; hence, a fractional period will inevitably cause discontinuity and create new frequency components. Non-uniform extinction ratios (ERs) also result in the splitting at a lower frequency, as shown in the 3rd columns in Figures S12a and S12b. In addition, the measurement noise in **O** will raise the noise floor in the rebuilt spectrum (see the 4th columns in Figures S12a and S12b). Next, we provide some numerical examples of reconstruction via 2D-DCT. Here, the reconstruction is performed based on the measured transmittance cube and computer generated spectra. The power sweep steps are set as N_{tFTS} = 50, in order to fully unfold the DCT map and avoid channel obscurity (see Note 6 for explanations). A white Gaussian noise of ±1 % is imposed to the interferograms to emulate the errors in the measurement. Figure S12c shows a discrete input spectrum with four spikes. The DCT result is shown in Figure S12d. Four distinctive spots, corresponding to four spikes in the spectrum, can be observed from the DCT map. For cross reference, we also show the fast Fourier transform (FFT) of the interferogram (see Figure S12e). According to the trajectories shown in Figure S8b, the 2D-DCT map is transformed into a 1D vector. Since N_{tFTS} exceeds the critical value, the raw vector encompasses redundant elements. The effective spectral information can be obtained by truncating the vector at two ends of the bandwidth, as shown in Figure S12f. The displayed spectrum is normalized using Parseval's theorem[S55]. Four spikes are discernible in the result. A spot in a 2D-DCT map has leakage in two dimensions (see Figure S12d). The broadening and splitting along f_{tFTS} will lead to the false spikes that are distant from the original one, as the map is flattened in columns (see Figure S8b). Therefore, the distribution of noise-like errors is not limited to the vicinity of spikes but is spread across the entire bandwidth. Also, due to the leakage of integral power, the intensities at spike locations are inaccurate. Since errors accumulate over all wavelengths, the reconstruction accuracy of a continuous spectrum is even worse (see Figures S12g-S12j). From above analysis, it is challenging to implement reconstruction with DCT

due to the large errors. In prior studies, the DCT method is applicable typically to the integrated FTSs using low-dispersion large-mode-area platforms, such as silica[S29] and laser-written[S56] waveguides, or some simple schemes[S41] that are easy to calibrate.

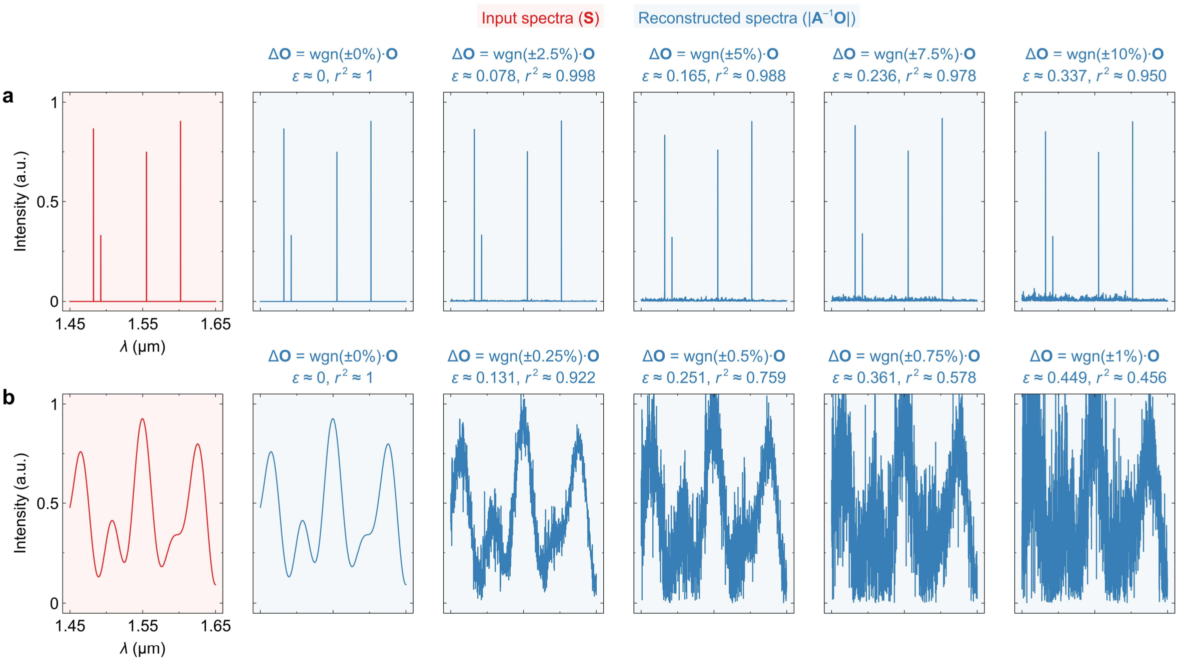

Figure S13 Reconstruction with pseudo inverse. Reconstruction of (**a**) a discrete spectrum and (**b**) a continuous spectrum utilizing pseudo inverse. White Gaussian noises [denoted as wgn(·)] of different levels are imposed to the interferograms (denoted as **O**). The input and reconstructed spectra (denoted as **S**) are displayed in red and blue, respectively. The relative errors (ε) and coefficients of determination (r^2) are also labeled.

As demonstrated in the early research on silicon spatial heterodyne spectrometers (SHS[S31]), it is also possible to reconstruct a spectrum from an interferogram using pseudo inverse. This method requires the calibration of the transmittance matrix (in this work, a cube) that depicts the transmission of an FTS at all wavelengths. In the matrix (or cube, denoted as **A**), each column is a distinctive fringe pattern (denoted as $\mathbf{a}_i$) at a specific wavelength. The output interferogram is thus a linear combination of fringes:

$$\mathbf{O} = \sum_{i=1}^{N_{2f}} s_i \mathbf{a}_i , \tag{S13}$$

where s_i denotes the intensity at the i-th wavelength channel, and N_{2f} denotes the channel capacity. This is equivalent to a matrix multiplication:

$$\mathbf{O} = \mathbf{AS}. \tag{S14}$$

In essence, the reconstruction of a spectrum is to determine the weight (i.e., s_i) of each fringe (i.e., $\mathbf{a}_i$), which can be realized by the inverse operation of Equation S14:

$$\mathbf{S} = \mathbf{A}^{-1}\mathbf{O}, \tag{S15}$$

where $\mathbf{A}^{-1}$ denotes the Moore-Penrose inverse of **A**. Compared to DCT, the pseudo-inverse method does not require ideal sinusoidal responses or any phase compensation since the dispersions of FSRs and ERs have been incorporated into the calibrated matrix/cube. If the recorded interferogram is completely accurate and noiseless, then any arbitrary spectrum can be precisely reverted with Equation S15. Due to the presence of temperature fluctuation (see Note 7) and fiber jittering, however, the interferogram usually contains errors:

$$\mathbf{O} = \mathbf{O}_{\text{ext}} + \Delta\mathbf{O}, \tag{S16}$$

where $\mathbf{O}_{\text{ext}}$ denotes the error-free interferogram, and $\Delta\mathbf{O}$ denotes the measurement error. As a consequence, the reconstruction results also involve errors ($\Delta\mathbf{S}$):

$$\Delta\mathbf{S} = \mathbf{A}^{-1}\Delta\mathbf{O}. \tag{S17}$$

Figure S13 shows the numerical examples of reconstruction with pseudo inverse. The white gaussian noises [denoted as wgn(·)] with varying strength are applied to the interferogram. Here, the transmittance cube is based on the measurement with $N_{\text{tFTS}} = 25$. The reconstruction accuracy is evaluated with relative error (ε) and coefficient of determination (r^2). All spectra are displayed with their absolute values. The errors become increasingly severe at a higher noise level. The reconstruction of a continuous spectrum is more susceptible to noises since an FTS collects information of all channels at each sampling and $\Delta\mathbf{O}$ will therefore influence the entire spectrum. Overall, the main drawback of the pseudo-inverse method is its sensitivity to noises.

It is possible to solve the inverse problem of Equation S14 via iterative optimization instead of pseudo inverse:

$$\mathbf{S} = \underset{\mathbf{S}}{\operatorname{argmin}}\left(\left\|\mathbf{AS}-\mathbf{O}\right\|_2^2\right), \tag{S18}$$

where argmin(·) denotes global minimum, and $||\cdot||_2$ denotes ℓ_2-norm. Nevertheless, the noises in **O** will still affect the convergence in the search for the optimal **S**. A generic solution is to add a regularization term (Ω) to Equation S18[S57]:

$$\mathbf{S} = \underset{\mathbf{S}}{\operatorname{argmin}}\left(\left\|\mathbf{AS}-\mathbf{O}\right\|_2^2 + \Omega\right). \tag{S19}$$

Ω is a penalty that imposes cost to the optimization function (i.e., $||\mathbf{AS} - \mathbf{O}||_2^2$), in order to bias the solution towards preconditioned features. With the use of Ω, the reconstruction error (i.e., $\Delta\mathbf{S}$) becomes[S58]:

$$\Delta\mathbf{S} = \left(\mathbf{I} - \mathbf{V\Psi V}^{\text{T}}\right)\mathbf{S} - \mathbf{V\Psi\Sigma}^{-1}\mathbf{U}^{\text{T}}\Delta\mathbf{O}, \tag{S20}$$

where **I** denotes the identity matrix. **U**, **Σ**, and **V** are the matrices produced via singular value decomposition

(SVD, $\mathbf{A} = \mathbf{U\Psi V}^T$), and $\mathbf{\Psi}$ denotes the filtering matrix determined by Ω[S58]. In Equation S20, the first [i.e., $(\mathbf{I} - \mathbf{V\Psi V}^T)\mathbf{S}$] and second (i.e., $\mathbf{V\Psi\Sigma}^{-1}\mathbf{U}^T\Delta\mathbf{O}$) parts represent the regularization penalty and noise perturbation, respectively. The proper selection of Ω will therefore balance the penalty and perturbation and mitigate the impact of measurement noises. This novel method has been applied to SHSs[S33] and digital FTSs (dFTS[S43,S44]) with various forms of Ω (e.g., compressed sensing and elastic networks). As a well-established approach, it has also been applied to computed tomography (CT[S59]) and radar detection[S60]. In this work, Ω is formulated as:

$$\Omega = \Omega_1 + \Omega_2 = \zeta_1^2 \left\| \mathbf{D}_2 \mathbf{S}_1 \right\|_2^2 + \zeta_2 \left\| \mathbf{D}_1 \mathbf{S}_2 \right\|_1, \tag{S21}$$

where ζ_i denotes the regularization parameter, $\mathbf{D}_i$ denotes the i-th order derivative operator, $\mathbf{S}_1$ and $\mathbf{S}_2$ denote the continuous and discrete components in the spectrum. The complete reconstruction formula can be thus expressed as:

$$\mathbf{S} = \underset{\mathbf{S}}{\operatorname{argmin}} \left(\left\| \tilde{\mathbf{A}}_{\mathbf{FFT}} \mathbf{S} - \mathrm{FFT}\left(\tilde{\mathbf{O}}\right) \right\|_2^2 + \zeta_1^2 \left\| \mathbf{D}_2 \mathbf{S}_1 \right\|_2^2 + \zeta_2 \left\| \mathbf{D}_1 \mathbf{S}_2 \right\|_1 \right), \tag{S22}$$

where $\tilde{\mathbf{A}}_{\mathrm{FFT}}$ denotes the matrix formed by the column vectors of FFT($\tilde{\mathbf{a}}_i$), $\tilde{\mathbf{a}}_i$ denotes the flattened fringe with zero-frequency components removed, and $\tilde{\mathbf{O}}$ denotes the interferogram after the component removal. More explanations of component removal and Fourier-domain operations can be found in Note 6. In essence, Ω is a precondition (or a priori) for the reconstruction result. Remarkably, Ω only sets a general range of possible characteristics that may occur in a spectrum. It does not require specific knowledge of spectral details before measurement. From Equation S21, the regularization term encompasses two parts, i.e., Ω_1 with ℓ_2-norm and Ω_2 with ℓ_1-norm. Ω_1 is commonly known as Tikhonov regularization[S61] that provides smoothening to spectra. To be specific, during iterations, Ω_1 will decrease when the derivative of the updated **S** has a smaller mean square; as a consequence, with a larger ζ_1, the spectrum will become more continuous. On the other hand, Ω_2 provides total-variation (TV[S59]) regularization that imposes more discrete features to the outcome of the optimization function. Generally, a spectrum is either continuous (or derivable) or discrete (or underivable); therefore, the proposed Ω can cover most naturally occurring spectral features. A hybrid spectrum can also be rebuilt using this method with non-zero values for both ζ_1 and ζ_2. The reconstruction of a spectrum with chaotic features will be discussed later. The weights of Ω_1 and Ω_2 are associated with ζ_1 and ζ_2, respectively. By using cross validation (CV), these hyperparameters can be automatically optimized without any manual selection. For K-fold CV, the basic concept is to divide the interferogram into K sets, one of which is utilized to produce a "reduced" solution to predict the elements in other sets. The prediction error reaches minimum

with the optimal ζ_1 and ζ_2[S62]:

$$\left(\zeta_1,\zeta_2\right)=\underset{\zeta_1,\zeta_2}{\operatorname{argmin}}\left[\sum\left|\tilde{\mathbf{a}}_i\mathbf{S}_i-\tilde{o}_i\right|^2\right], \tag{S23}$$

where $\mathbf{S}_i$ denotes the solution with $\tilde{\mathbf{a}}_i$ left out in the cube, and $\tilde{o}_i$ denotes the *i*-th element in the interferogram. For a smooth spectrum, ζ_2 will descend to zero, and only Ω_1 will function in the regularization term. Similarly, when the input spectrum is spike- or step-like, the penalty will be dominated by Ω_2. If multiple features are hybridized in a spectrum, then both ζ_1 and ζ_2 will be non-zero, with their optimal values selected by CV. In this work, the number of subsets is chosen as K = 5. The search for global optimum is enabled by a standard least-squares solver[S63]. The CV procedure is embedded within the iterative optimization. The search for the optimal ζ_1 and ζ_2 is performed over an 8×8 space arranged in the log scale. From Equation S22, the spectrum is decomposed into two components in accordance with their spectral features, i.e., $\mathbf{S} = \mathbf{S}_1 + \mathbf{S}_2$. To ensure a single-vector input, the formula is modified as:

$$\mathbf{S}=\underset{s}{\operatorname{argmin}}\left(\left\|\begin{bmatrix}\tilde{\mathbf{A}}_{\mathrm{FFT}}\\ \tilde{\mathbf{A}}_{\mathrm{FFT}}\end{bmatrix}\begin{bmatrix}\mathbf{S}_1 & \mathbf{S}_2\end{bmatrix}-\tilde{\mathbf{O}}\right\|_2^2+\zeta_1^2\left\|\begin{bmatrix}\mathbf{D}_2\\ 0\end{bmatrix}\begin{bmatrix}\mathbf{S}_1 & \mathbf{S}_2\end{bmatrix}\right\|_2^2+\zeta_2\left\|\begin{bmatrix}0\\ \mathbf{D}_1\end{bmatrix}\begin{bmatrix}\mathbf{S}_1 & \mathbf{S}_2\end{bmatrix}\right\|_1\right), \tag{S24}$$

Thus, the searching vector becomes $[\mathbf{S}_1, \mathbf{S}_2]$, and the transmittance cube becomes $[\tilde{\mathbf{A}}_{\mathrm{FFT}}, \tilde{\mathbf{A}}_{\mathrm{FFT}}]^{\mathrm{T}}$. In this work,

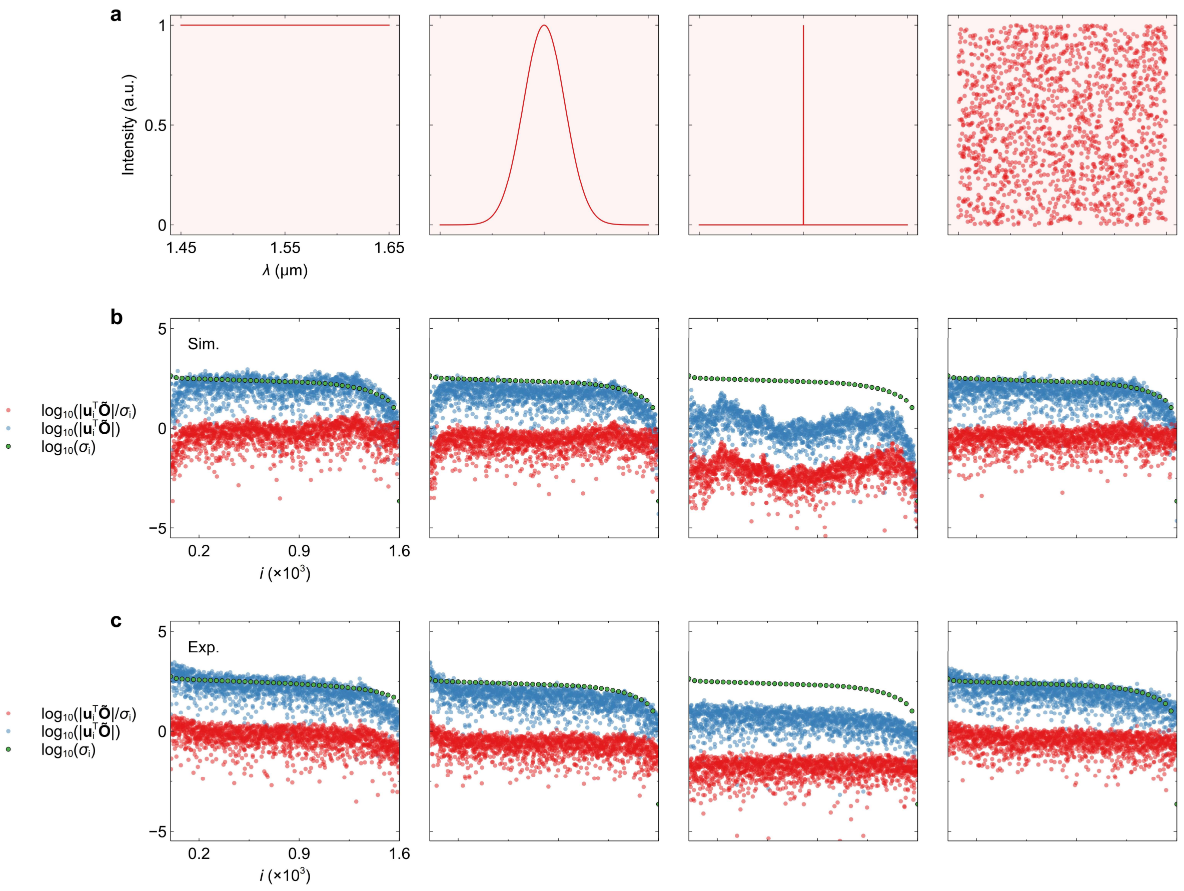

Figure S14 Picard plots. (**a**) Generated testing spectra. Picard plots for the (**b**) simulation and (**c**) experimental results.

spectrum reconstruction is realized using the least-squares QR-decomposition (LSQR) module in IRtools[S63]. It is also easy to reproduce our results with other open-source packages (e.g., Pylops[S64]) since Equation S24 is in the standard form of a regularization problem.

To further verify the effectiveness of the proposed method, the solvability of Equation S19 is assessed with the Picard plot[S65]. Based on SVD, the "naïve" solution to the inverse problem can be written as:

$$\mathbf{S} = \sum_{i=1}^{N_{2f}} \frac{\mathbf{u}_\mathrm{i}^\mathrm{T}\mathbf{O}}{\sigma_\mathrm{i}} \mathbf{v}_\mathrm{i}, \tag{S25}$$

where $\mathbf{u}_\mathrm{i}$ denotes the i-th left singular vector, $\mathbf{v}_\mathrm{i}$ denotes the i-th right singular vector, σ_i denotes the singular value, and N_{2f} denotes the capacity. From Equation S25, it is revealed that an ideal reconstruction result is formed on the basis of right singular vectors (i.e., $\mathbf{v}_\mathrm{i}$) that are weighted by SVD coefficients (i.e., $\mathbf{u}_\mathrm{i}^\mathrm{T}\mathbf{O}/\sigma_\mathrm{i}$). It is

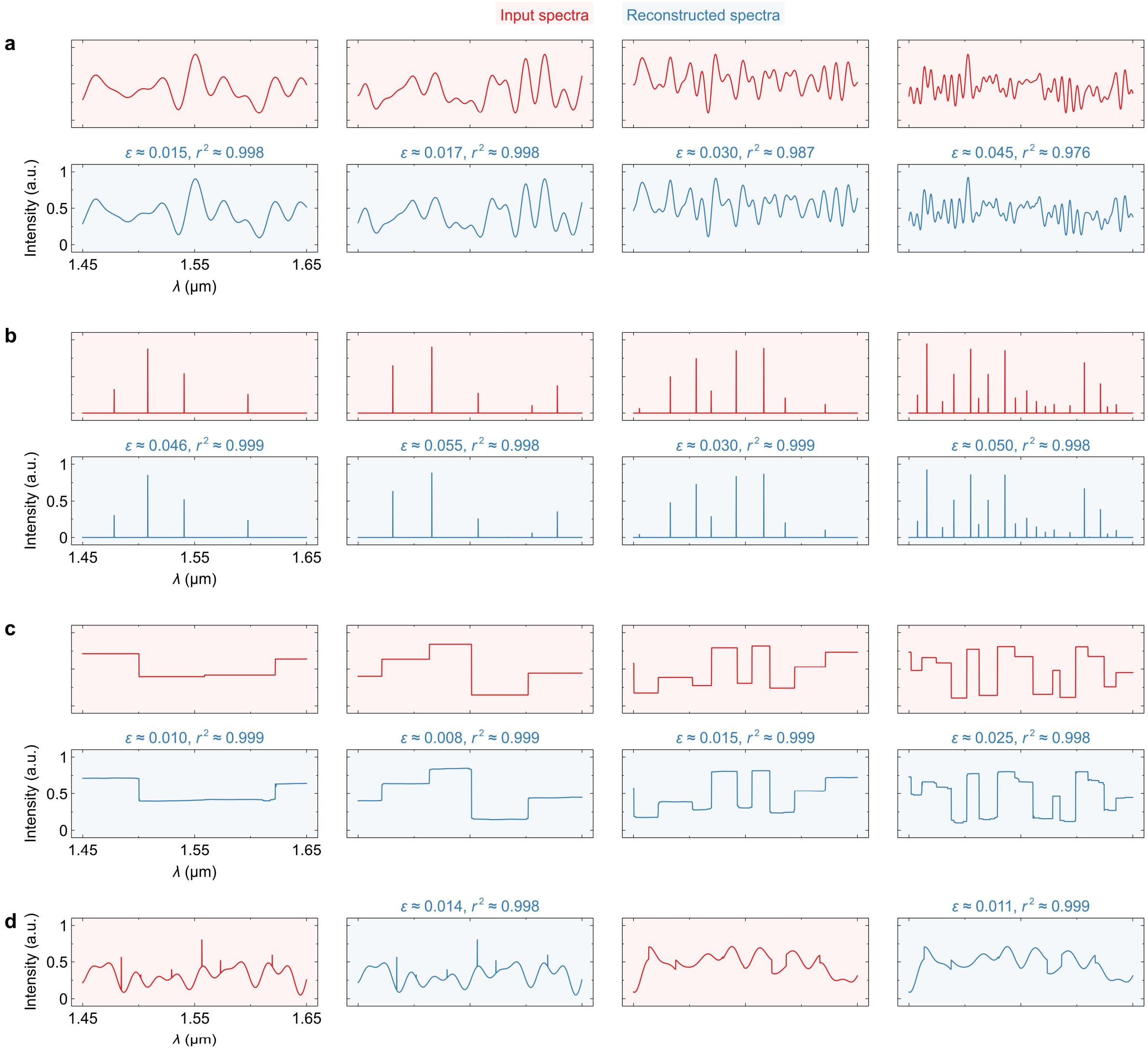

Figure S15 Numerical test. Numerical spectrum reconstruction of (**a**) smooth spectra, (**b**) sparse spectra, (**c**) step spectra, and (**d**) hybrid spectra. The relative errors (ε) and coefficients of determination (r^2) are labeled.

thus essential to ensure that the SVD coefficient levels off to a finite value (commonly known as the Picard condition[S65]); otherwise, the integral of N_{2f} channels will be infinite and the iterative process will suffer from a poor convergence. Four different types of spectra (i.e., plateau, Gaussian, spike, and random functions) are used for testing, as shown in Figure S14a. In Figures S14b and S14c, we calculate the absolute values of SVD coefficients, sampling weights ($|\mathbf{u}_i^T\tilde{\mathbf{O}}|$), and singular values in a semi-log plot. The simulated and measured SVD coefficients do not overall increase even at a high index, demonstrating that the Picard condition is met, and that a convergent solution can always be obtained in Equation S19.

Figure S15 shows some numerical reconstruction examples. Here, we mainly consider the spectra with smooth, sparse, and step features, as shown in Figures S15a-S15c. The hybrid spectra with multiple features are also discussed, as shown in Figure S15d. To emulate environmental perturbations, we use the recorded temperature fluctuations (see Note 7) and calculated temperature sensitivities (see Figure S1f) to generate noises in the interferogram. A high reconstruction accuracy is attained for all different types of spectra. The reconstruction was implemented with MATLAB on a 24-core 3-GHz Intel Xeon Gold CPU. If all the optimal hyperparameters (i.e., ζ_i) are known, then the fixed-parameter reconstruction time (FPRT) will be as short as < 1 s. When ζ_1 and ζ_2 are free to optimize, the time cost will increase due to the CV procedure. In the worst case, when the full 8×8 searching space must be traversed, the total reconstruction time is < 60 s. There are several strategies to expedite the reconstruction. First, since the iterative least-squares solver relies heavily on matrix multiplication, the reconstruction can be drastically accelerated by employing a GPU. Second, it is possible to train a deep-learning network to identify spectral features directly from the interferogram and determine ζ_i without CV[S25], which may reduce the reconstruction period to a single FPRT.

From above analysis, we have numerically and experimentally demonstrated the feasibility in precisely retrieving spectra using the regularized iterative method. The applications in spectroscopy, communications, and imaging typically require recovering a spectrum that is either smooth (e.g., spontaneous emission and near-infrared spectroscopy), sparse (e.g., WDM signals and atomic spectroscopy), or step-like (e.g., rejection band of an optical filter). All these scenarios can be covered by the proposed 2D-FTS scheme. Nevertheless, it is possible that a spectrum may have a great amount of randomly distributed high-frequency components and a seemingly chaotic response. Here, we will explore the capability in retrieving a spectrum with a high degree of randomness. It is difficult to experimentally produce random testing spectra. As a proof of concept, we use the measured transmittance cube and generated random functions to perform a numerical test. The Fourier-Wiener series are leveraged to create sequences with controllable randomness[S66]:

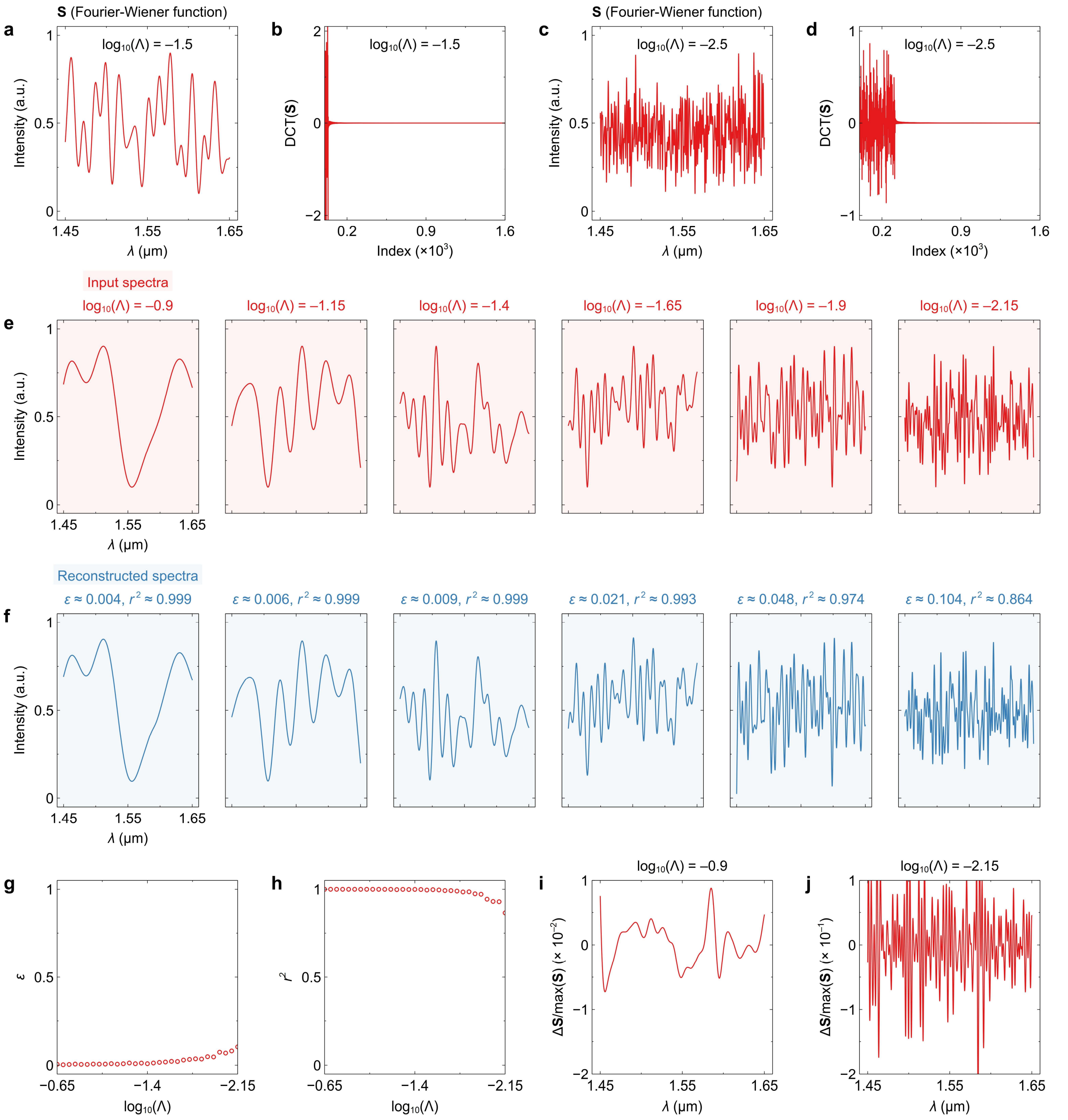

Figure S16 Reconstruction of random spectra. (**a-d**) Testing functions and their discrete Fourier transform (DCT) with different Λ. Here, Λ is a parameter that determines the fraction of random responses in the Fourier domain. (**e**) Input and (**f**) reconstructed spectra with varying Λ. The relative errors (ε) and coefficients of determination (r^2) are also labeled. Calculated (**g**) ε and (**h**) r^2 with varying Λ. Normalized deviations [Δ**S**/max(**S**)] with (**i**) $\log_{10}(\Lambda) = -0.9$ and (**j**) $\log_{10}(\Lambda) = -2.15$.

$$\mathbf{S} = \sum_{i=-m}^{m} c_i D(x - ih), \quad \text{(S26)}$$

$$D(x) = \frac{\sin\left[(2m+1)\pi x\right]}{(2m+1)\sin(\pi x)}, \quad \text{(S27)}$$

$$m = \lfloor 1/\Lambda \rfloor, \quad \text{(S28)}$$

where x is a N_{2f}-point sequence ranging from 0 to 1, c_i is the i-th element in a computer generated random sequence, and Λ is a parameter that tunes the randomness of **S**. Given a relatively large Λ, the function output resembles a 1D random walk, as shown in Figure S16a. The reduction of Λ will increase the proportion of random responses in the DCT domain (see Figure S16d), making the generated spectrum more chaotic (see Figure S16c). By using Fourier-Wiener series, we can reveal the evolution of reconstruction accuracy when the input spectrum transitions from a smooth function to a chaotic function. Figure S16e shows the testing spectra with the parameter ranging from $\log_{10}(\Lambda) = -0.9$ to -2.15. The corresponding reconstruction results are listed in Figure S16f. Figures S16g and S16h respectively show the relative errors (ε) and coefficients of determination (r^2) as functions of Λ. In Figures S16i and S16j, we also derive the normalized deviations [i.e., $\Delta\mathbf{S}/\max(\mathbf{S})$] with $\log_{10}(\Lambda) = -0.9$ and -2.15. The calculated ε and r^2 curves are still quite flat even with a high degree of randomness in the spectrum. At $\log_{10}(\Lambda) = -2.15$, the input spectrum becomes chaotic, but a high reconstruction accuracy of $\varepsilon < 0.1$ and $r^2 > 0.85$ can still be achieved. The slight degradation in accuracy may results from the rivalry between Tikhonov and TV regularizations during the automatic CV procedure, as a chaotic spectrum is neither continuous nor discrete, and the regularization penalty cannot fully compensate for the noise perturbation. Form these results, it is demonstrated the regularized iterative optimization has the potential to cover a wide range of spectral features and serve as a generic reconstruction method.

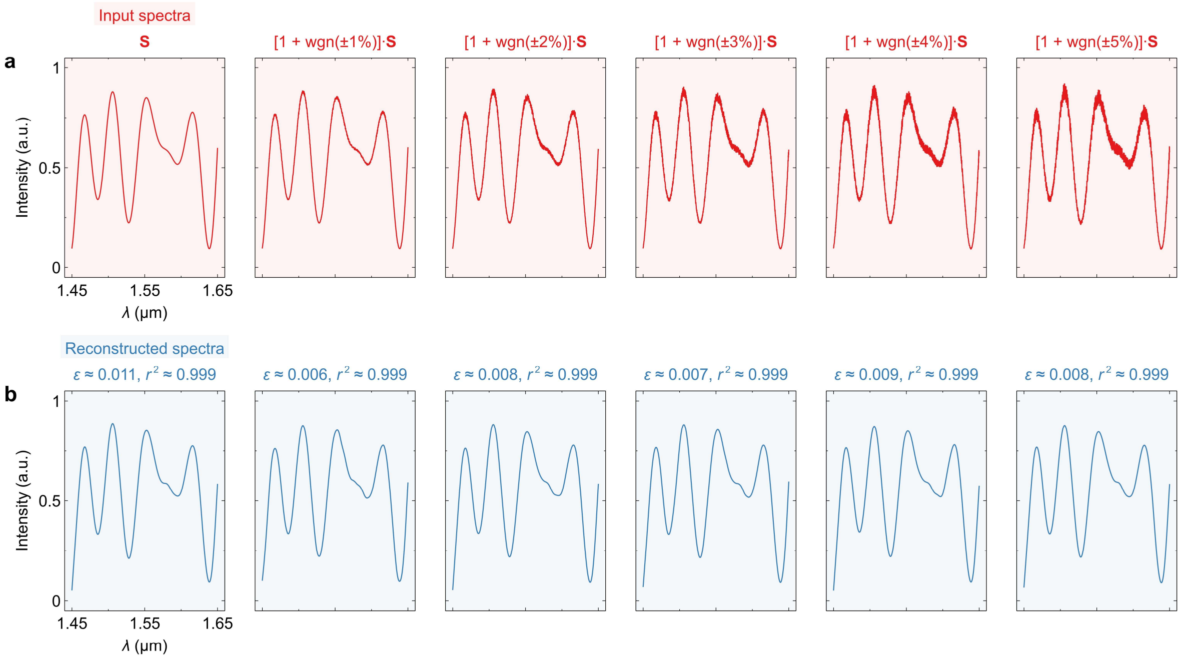

Figure S17 Reconstruction under input instability. (**a**) Testing input spectra with intentionally introduced instability. Here, wgn(·) denotes white Gaussian noise. (**b**) Reconstructed spectra under varying levels of instability. The relative errors (ε) and coefficients of determination (r^2) are also labeled.

Due to platform vibration and fiber jittering, the input spectrum can be instable during measurement. In Figure S17, we give an example of spectrum reconstruction with varying levels of intentionally introduced instability. Here, a white Gaussian noise [i.e., wgn(·)] is employed to emulate the undulation in the spectrum, as shown in Figure S17a. The reconstruction results are shown in Figure S17b. It can be found that the noises are filtered out and all rebuilt spectra are "smoothened". This effect can be explained as follows. According

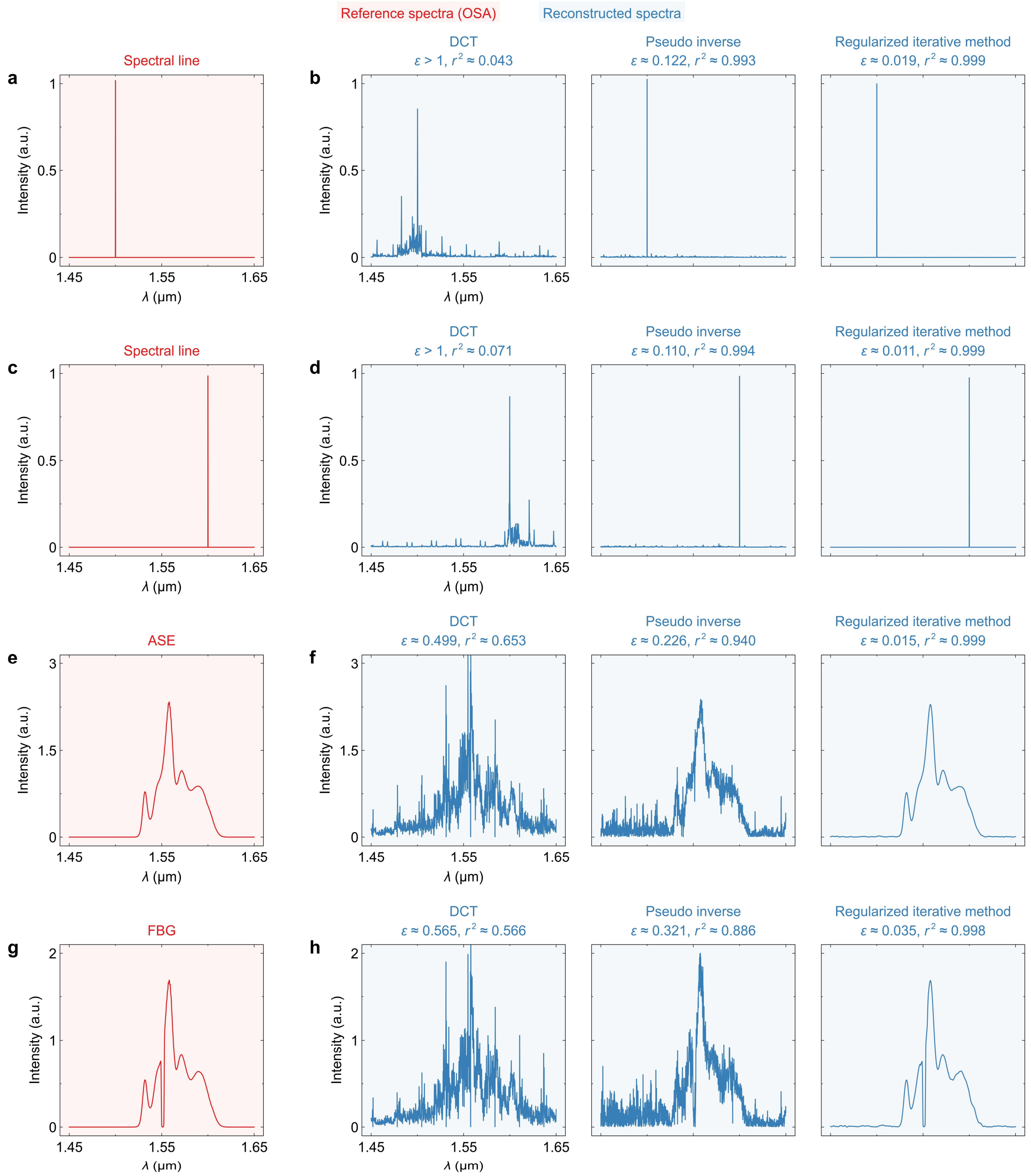

Figure S18 Comparison of reconstruction with different methods. Reference and reconstruction results of (**a**-**d**) a single spectral line, (**e**, **f**) amplified spontaneous emission (ASE), and (**g**, **h**) the response of a fiber Bragg grating (FBG). The 1st, 2nd, and 3rd columns of the right panel show the reconstruction results based on discrete cosine transform (DCT), pseudo inverse, and the regularized iterative method, respectively. The relative errors (ε) and coefficients of determination (r^2) are also labeled.

to the Picard plot shown in the 4th column of Figure S14, the perturbation component in **S** will not result in the divergence of the solution. By using CV, the input spectrum will be automatically recognized as a smooth one. The Tikhonov-regularization term (i.e., Ω_1, see Equation S21) will thus cancel out the non-smooth part. This effect holds valid as long as the instability of a spectrum does not overwhelm its key features; otherwise, the undulation will also appear in the reconstructed spectrum, as discussed in Figure S16. Our measurement setup (e.g., positioning stage and fiber holder) is mechanically stable. All testing spectra were produced with fiber-connected commercial optical sources and filters. The input instability was well controlled and limited to a relatively low level during the experiment, while the remnant perturbation can be numerically resolved.

In Figure S18, we compare the experimental reconstruction results based on three methods discussed above: DCT, pseudo inverse, and regularized iterative optimization. The testing spectra include spectral lines, amplified spontaneous emission, and the response of a fiber Bragg grating, as an example. The DCT results have a high noise floor and a great amount of false peaks. By using pseudo inverse, a single spectral line can be retrieved with a peak signal-to-noise ratio of PSNR ≈ 15 dB. However, it is still impossible to reconstruct a smooth or step spectrum. Our proposed method, in contrast, supports small errors and low noises for all different types of spectra. Table S2 compares different reconstruction methods in integrated FTSs.

Table S2. Comparison of reconstruction methods in integrated Fourier-transform spectrometry.

Design	Method	Formula	Iterative	Noise sensitivity	Solvable spectral feature			
					Smooth	Sparse	Step	Hybrid
SHS[S30]	DCT	$\mathbf{S} = \mathrm{DCT}(\mathbf{O})$	N [(a)]	High	Y	Y	Y	Y
SHS[S32]	Pseudo inverse	$\mathbf{S} = \mathbf{A}^{-1}\mathbf{O}$	N	High	Y	Y	Y	Y
SHS[S34]	Reg. (LASSO)	$\mathbf{S} = \mathrm{argmin}(\zeta\|\|\mathbf{S}\|\|_1)$	Y [(b)]	Low	Y	Y	N	N
dFTS[S43]	Reg. (elastic-net)	$\mathbf{S} = \mathrm{argmin}(\zeta_1^2\|\|\mathbf{S}\|\|_2^2 + \zeta_2\|\|\mathbf{S}\|\|_1)$	Y	Low	Y	Y	N	N
**This work**	**Reg. (Tikhonov/TV)**	$\mathbf{S} = \mathrm{argmin}(\|\|\tilde{\mathbf{A}}_{\mathrm{FFT}}\mathbf{S} - \tilde{\mathbf{O}}\|\|_2^2 + \zeta_1^2 \|\|\mathbf{D}_2\mathbf{S}_1\|\|_2^2 + \zeta_2\|\|\mathbf{D}_1\mathbf{S}_2\|\|_1)$	Y	Low	**Y**	**Y**	**Y**	**Y**

DCT, discrete cosine transform.

LASSO, least absolute shrinkage and selection operator.

TV, total variation.

Reg., regularization iterative method.

[(a)]No.

[(b)]Yes.

Note 9. Extended experimental data

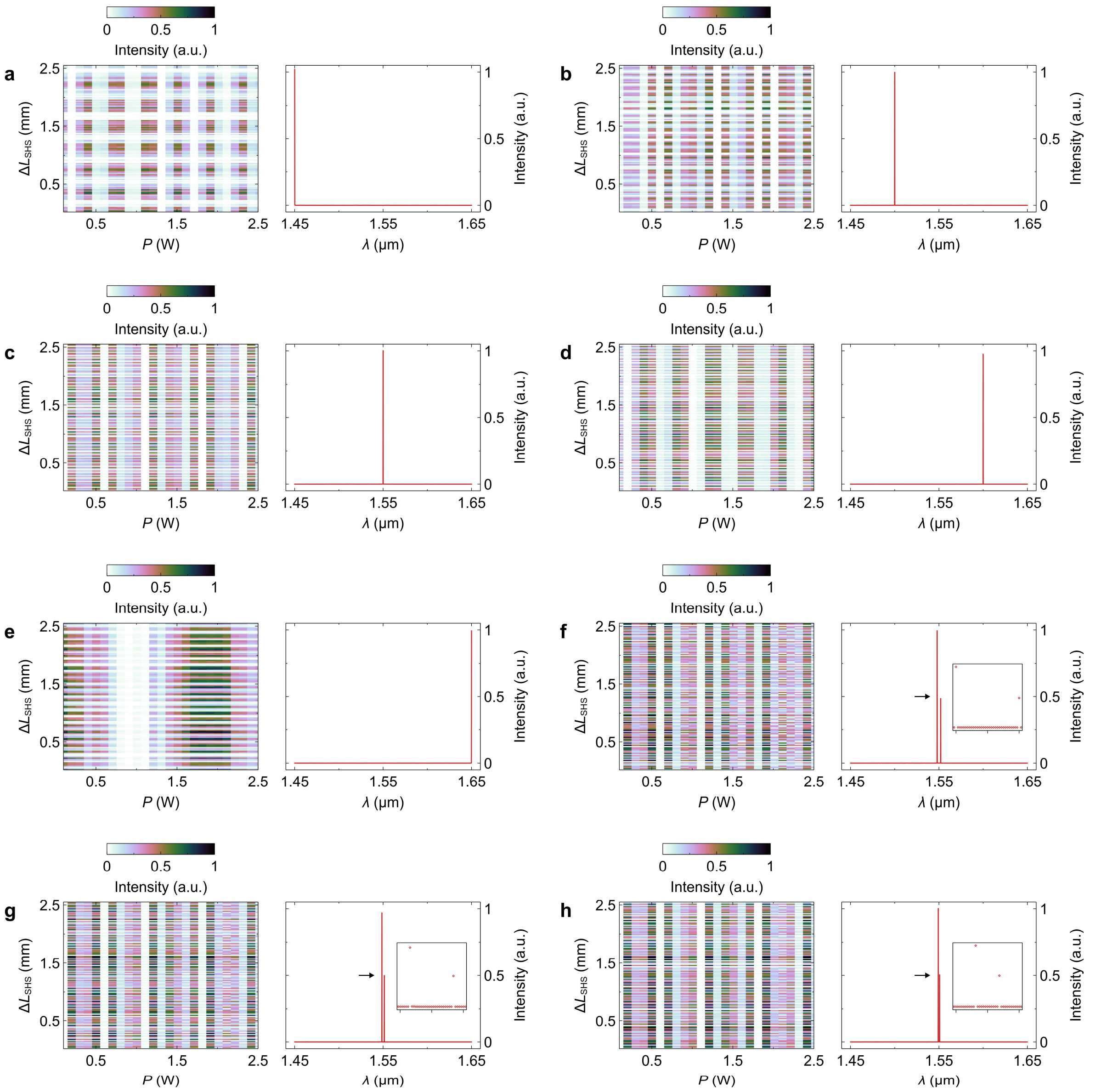

Figure S19 Extended experimental data (part I). Measurement and reconstruction results of (**a-e**) a single spectral line and (**f-h**) dual spectral lines. Left panels: measured interferograms. Right panels: reconstructed spectra. Insets: enlarged views of spectra around the wavelengths indicated by the arrows. The two-dimensional interferograms and reconstructed spectra shown in Figures S19a-S19h correspond to the results shown in Figures 5a-5b.

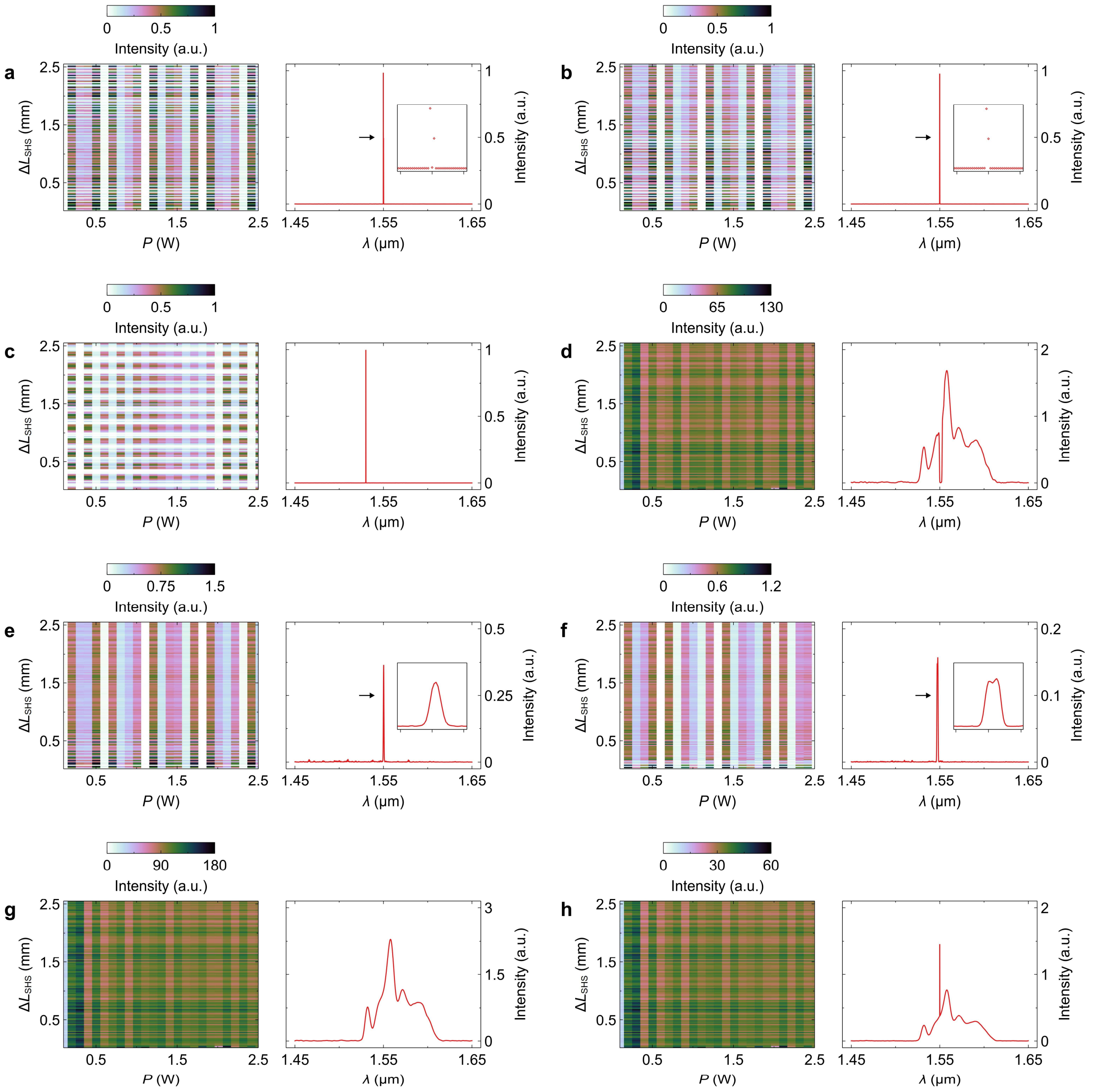

Figure S20 Extended experimental data (part II). Measurement and reconstruction results of (**a**-**c**) dual lines, (**d**) the response of a fiber Bragg grating (FBG), (**e**-**f**) the responses of arrayed waveguide gratings (AWG), (**g**) amplified spontaneous emission (ASE), and (**h**) ASE with a spectral line. Left panels: measured interferograms. Right panels: reconstructed spectra. Insets: enlarged views of spectra around the wavelengths indicated by the arrows. The two-dimensional interferograms and reconstructed spectra correspond to the results shown in Figures 5b-5h.

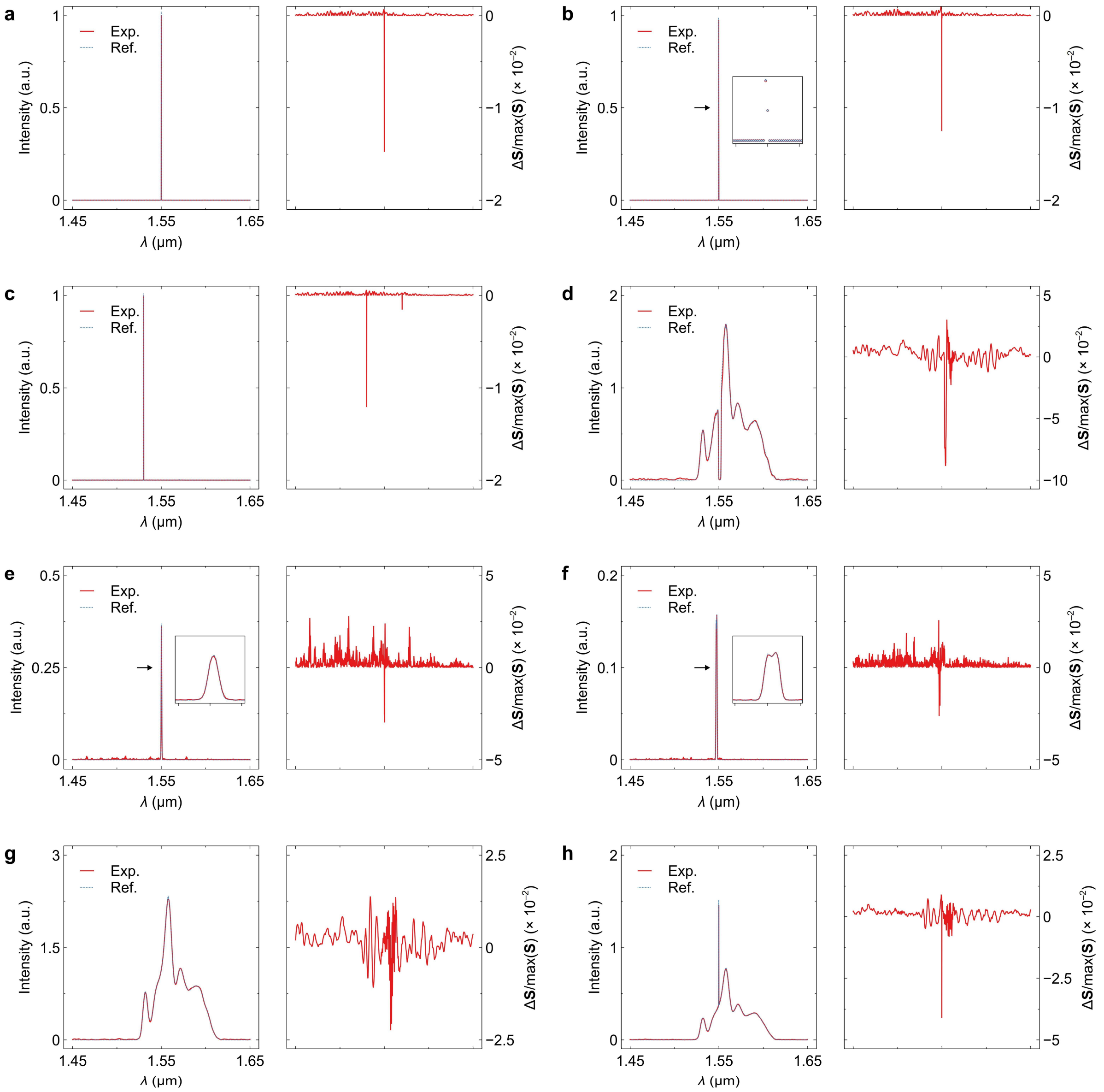

Figure S21 Analysis of reconstruction results. Reconstruction of (**a**) a single spectral line, (**b**) dual spectral lines, (**c**) high-contrast spectral lines, (**d**) the response of a FBG, (**e**) the response of a single channel of an AWG, (**f**) the response of dual channels of an AWG, (**g**) the response of ASE, and (**h**) an ASE superimposed with a single spectral line. In each figure, the left panel shows the experimental reconstruction result and reference spectrum from a commercial OSA, while the right panel shows normalized deviations [$\Delta\mathbf{S}/\max(\mathbf{S})$]. Here, $\Delta\mathbf{S}$ denotes the difference between the rebuilt and reference spectra, and $\max(\mathbf{S})$ denotes the maximum element in the reference spectrum. The insets show the enlarged views of spectra around the wavelength ranges indicated by the arrows. For the reconstruction of a sparse spectrum, errors typically appear at the peak locations, which can be explained as follows. The initial guess of an unknown spectrum is an all-zero sequence. In a sparse spectrum, most elements are close to zero, except for the peaks. Consequently, most near-zero elements will reach its optimum after a few iterations, but will continue to be updated, resulting in a noise-like background. In the meantime, the solving of peak values requires more iterations. The cumulative errors from other elements will thus affect the accuracy of peak reconstruction. The reconstruction of an ideally sparse or smooth spectrum typically has a high accuracy of $\Delta\mathbf{S}/\max(\mathbf{S}) < 2\%$. However, the background noise will increase when reconstructing the sharp but spreading response of an AWG (see Figures S19e and S19f). Albeit being derivable, the AWG response has a fast-changing derivative around the resonant wavelength and a majority of near-zero elements. During cross validation, the ℓ_1-norm term will therefore compete with the ℓ_2-norm term and result in a small but non-

zero ζ_2, which imposes a higher noise floor to the retrieved spectrum and slightly reduces the peak signal-to-noise ratio to PSNR ≈ 20 dB. Relatively larger errors [$\Delta\mathbf{S}/\max(\mathbf{S}) > 5\%$] can also be found in the reconstruction of the hybrid spectra shown in Figures S19d and S19h, which is also caused by the imperfect selection of two hyperparameters. Nevertheless, for all tested spectra with various features, small relative errors of $\varepsilon < 0.1$ and high coefficients of determination of $r^2 > 0.99$ can be attained. A higher accuracy can be obtained by transforming the spectrum with a basis (e.g., DCT[S19] and Lorentzian decomposition[S28]) that aligns better with pre-conditioned features. FBG, fiber Bragg grating. AWG, arrayed waveguide grating. ASE, amplified spontaneous emission.

Note 10. Discussion of scheme scalability

In this research, the concept and implementation of two-dimensional Fourier-transform spectrometry (2D-FTS) are discussed. In comparison to conventional 1D-FTS, the major advantage is that a fine resolution and a broad bandwidth can be simultaneously achieved. As a proof of concept, a 2D-FTS is realized by combining a tunable FTS (tFTS) and a spatial heterodyne spectrometer (SHS). Based on the computational method, we demonstrate a fine spectral resolution of $\delta\lambda_{2f}$ = 125 pm beyond the Rayleigh criterion throughout a 200-nm bandwidth. The demonstrated capacity is as large as N_{2f} > 1601, a new record among all reported FTSs. By comparison, a stand-alone tFTS requires a heating power of P_{max} > 100 W to accomplish a comparable level of resolution. Also, when using a stand-alone SHS to attain the same bandwidth, it is necessary to integrate > 2000 Mach-Zehnder interferometers on a single die, which is challenging at the current state of art. However, such a scheme has two main drawbacks. First, the tFTS still requires a relatively high heating power of P_{max} ≈ 2.4 W to decorrelate all free spectral ranges (FSR) of the SHS. Second, although the Fellgett's advantage is maintained in this scheme, the Jacquinot's advantage is somehow diminished since the launched etendue is separated into 128 ports in the SHS. Scaling to a finer resolution and a larger capacity may therefore result in a degradation in power efficiency and signal detectivity.

Notably, the proposed 2D-FTS is a flexible scheme that is not limited to the combination of a tFTS and a SHS but can be realized with any form of FTS designs. Actually, the power and etendue problems are readily rectifiable. To validate the scheme scalability, in this section, we will provide two additional design examples: a modified 2D-FTS and a high-performance 3D-FTS. For the modified 2D-FTS, we will prove the viability of attaining the same resolution and bandwidth with a single output port and a drastically reduced drive power (< 120 mW). For the 3D-FTS, the 2D-FTS concept will be extended to its three-dimensional form with a finer resolution (i.e., $\delta\lambda_{2f}$ < 31.25 pm) and a larger capacity (i.e., N_{2f} > 6401). These FTS designs are based on the elements and experimental results readily demonstrated in this work. The feasibility of higher-dimensional FTS will also be discussed.

A. Modified two-dimensional Fourier-transform spectrometer

Figure S22a shows the layout of the modified 2D-FTS. The device consists of a tFTS and a digital FTS (dFTS). The tFTS is formed by a balanced Michelson interferometer (MI) rather than a Mach-Zehnder interferometer (MZI), as shown in Figure S22b. The use of an MI doubles the optical path length (OPL) change from $\Delta n_g L_{tFTS}$ to $2\Delta n_g L_{tFTS}$ since its interference arm is reflective. Here, the arm length is set as L_{tFTS} = 1.5 cm, the same as

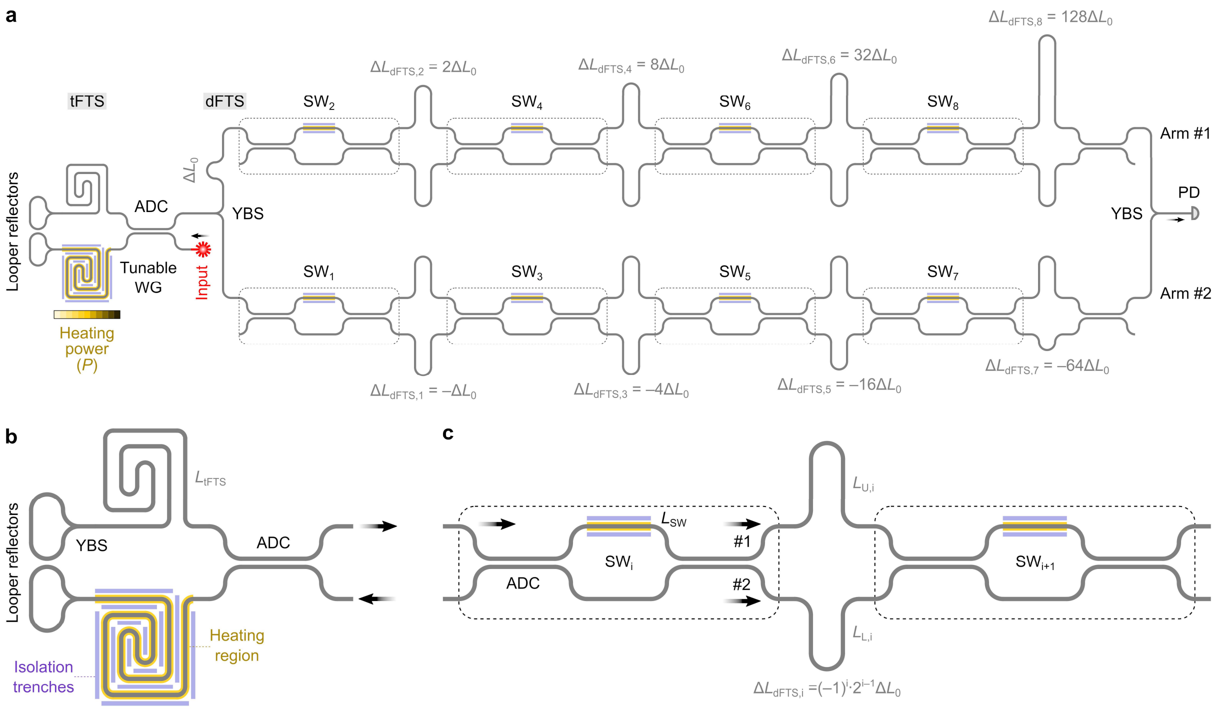

Figure S22 Modified two-dimensional Fourier-transform spectrometer (2D-FTS). Schematic layout of the (**a**) modified 2D-FTS. The enlarged views of the (**b**) tunable FTS (tFTS) and (**c**) a repeating unit in the digital FTS (dFTS). In the modified scheme, the spatial heterodyne spectrometer (SHS) is replaced by a dFTS, in order to ensure single-port detection and reduce the footprint. Furthermore, a Michelson interferometer (MI) instead of a Mach-Zehnder interferometer (MZI) is employed in the tFTS, so that the effective optical path length (OPL) can be doubled. Some isolation trenches are etched alongside the heating region of the spiral waveguides to improve the thermo-optical tuning efficiency. ADC, adiabatic directional coupler. YBS, PD, photodetector. Y-branch splitter. WG, waveguide. SW, switch.

in the original design. The tuning efficiency is further enhanced by etching isolation trenches alongside the tunable waveguide. In the spiral, thermal isolation is inserted between adjacent straight sections (see Figure S23b), whereas the short bends are not isolated, in order to release structural stress. At each arm, the output ports of a Y-branch splitter (YBS) are connected to each other to serve as a looper reflector. Two interference arms are directed to an adiabatic directional coupler (ADC). One port of the ADC is used as input, while the other one is routed to the dFTS. The dFTS is composed of two YBSs and two interference arms (denoted as #1 and #2). Each arm is formed by several repeating units with switchable effective OPLs, as shown in Figure S22c. Each unit has two MZI switches and a pair of asymmetric delay lines between them. The MZI switch is based on two broadband ADCs and two balanced arms. In the arm #1, the upper delay lines are longer than the lower delay lines (i.e., $L_{\mathrm{U,i}} > L_{\mathrm{L,i}}$, where i is an even number), whereas in the arm #2, the upper delay lines are shorter than the lower delay lines (i.e., $L_{\mathrm{U,i}} < L_{\mathrm{L,i}}$, where i is an odd number). The lower delay lines in the arm #1 and the upper delay lines in the arm #2 have a constant length (denoted as L_{ref}), as a reference:

$$L_{\mathrm{L,i}} = L_{\mathrm{ref}}, \quad \mathrm{mod}(i,2)=0, \tag{S29}$$

$$L_{\mathrm{U,i}} = L_{\mathrm{ref}}, \quad \mathrm{mod}(i,2)=1. \tag{S30}$$

For the delay lines cascaded after the *i*-th switch (denoted as SW_i), the length asymmetry (denoted as $\Delta L_{\mathrm{dFTS,i}}$) is:

$$\Delta L_{\mathrm{dFTS,i}} = L_{\mathrm{L,i}} - L_{\mathrm{U,i}} = (-1)^i \cdot 2^{i-1} \Delta L_0, \tag{S31}$$

where ΔL_0 denotes the base length. The number of usable states (denoted as N_{dFTS}) is thus tied to the number of switches (denoted as N_{SW}):

$$N_{\mathrm{dFTS}} = 2^{N_{\mathrm{SW}}}. \tag{S32}$$

We impose additional asymmetry of ΔL_0 to arm #1, so the variation range of overall arm-length differences (denoted as ΔL_{dFTS}) becomes:

$$\Delta L_{\mathrm{dFTS}} = \Delta L_{\mathrm{dFTS,min}} \sim \Delta L_{\mathrm{dFTS,max}} = \Delta L_0 \sim 2^{N_{\mathrm{SW}}} \Delta L_0, \tag{S33}$$

where $\Delta L_{\mathrm{dFTS,min}}$ and $\Delta L_{\mathrm{dFTS,max}}$ respectively denote the minimum and maximum differences. The π phase shift induced by the switch process can be compensated for with an additional tuning section (not displayed in the figure).

In such a device, light is modulated by the tFTS and dFTS in succession. The interferogram is thus a 2D pattern with variations of heating power in the tFTS and switch steps in the dFTS. The 2D-FTS response can be depicted as a 3D cube formed by a series of 2D fringes at varying wavelengths. In principle, this scheme is equivalent to the tFTS/SHS scheme proposed in the main manuscript, but it is implemented in a different manner. The modified scheme offers several advantages:

1. Due to the use of the MI and isolation trenches, the power consumption can be reduced to < 120 mW.
2. The dFTS has the capability to support a great number of switch states with a single output port, thereby resolving the etendue issue. The increase in N_{dFTS} also contributes to the expansion of FSR of the dFTS and the reduction of the heating power required in the tFTS.
3. Due to the folding of light paths in the tFTS and dFTS, the device footprint can be minimized.

Next, we will give the optimization flow of this design. From above analysis, the resolutions of the tFTS and dFTS can be expressed as:

$$\delta\lambda_{\mathrm{tFTS}} = \frac{\lambda^2}{2\Delta n_g L_{\mathrm{tFTS}}}, \tag{S34}$$

$$\delta\lambda_{\mathrm{dFTS}} = \frac{\lambda^2}{n_{\mathrm{g}}\Delta L_{\mathrm{dFTS,max}}}, \tag{S35}$$

where λ denotes the wavelength, $\delta\lambda_{tFTS}$ denotes the tFTS resolution, $\delta\lambda_{dFTS}$ denotes the dFTS resolution, n_g denotes the group index, and Δn_g denotes the change in n_g. The factor "1/2" in Equation S34 results from the light-path folding in the MI. The corresponding FSRs can be written as:

$$\mathrm{FSR}_{\mathrm{tFTS}} = \frac{N_{\mathrm{tFTS}}\delta\lambda_{\mathrm{tFTS}}}{2} = \frac{N_{\mathrm{tFTS}}\lambda^2}{4\Delta n_{\mathrm{g}} L_{\mathrm{tFTS}}}, \tag{S36}$$

$$\mathrm{FSR}_{\mathrm{dFTS}} = \frac{N_{\mathrm{dFTS}}\delta\lambda_{\mathrm{dFTS}}}{2} = \frac{N_{\mathrm{dFTS}}\lambda^2}{2 n_{\mathrm{g}} \Delta L_{\mathrm{dFTS,max}}}, \tag{S37}$$

where N_{tFTS} denotes the number of power sweep steps. From Equations S34-S37, we derive the resolution, bandwidth, and critical condition for the modified 2D-FTS:

$$\delta\lambda_{\mathrm{f}} = \delta\lambda_{\mathrm{dFTS}} = \frac{\lambda^2}{n_{\mathrm{g}}\Delta L_{\mathrm{dFTS,max}}}, \tag{S38}$$

$$\mathrm{BW} = \mathrm{FSR}_{\mathrm{tFTS}} = \frac{N_{\mathrm{tFTS}}\lambda^2}{4\Delta n_{\mathrm{g}} L_{\mathrm{tFTS}}}, \tag{S39}$$

$$\delta\lambda_{\mathrm{tFTS}} = \frac{\lambda^2}{\Delta n_{\mathrm{g}} L_{\mathrm{tFTS}}} < \mathrm{FSR}_{\mathrm{dFTS}} = \frac{N_{\mathrm{dFTS}}\lambda^2}{2 n_{\mathrm{g}}\Delta L_{\mathrm{dFTS,max}}}, \tag{S40}$$

where $\delta\lambda_f$ denotes the resolution at the Rayleigh criterion, and BW denotes the operation bandwidth. The target resolution is $\delta\lambda_f$ = 250 pm, which can be enhanced to $\delta\lambda_{2f} = \delta\lambda_f/2$ =125 pm using the computational method. According to Equation S38 and the results shown in Note 6, the maximum arm-length difference is chosen as $\Delta L_{dFTS,max}$ = 2.55 mm. Each interference arm in the dFTS contains four stages, yielding the number of switch states of $N_{dFTS} = 2^8 = 256$. Thus, in the design, the unknown parameters are the maximum heating power (P_{max}) required to meet Equation S40 and the number of power sweep steps required to achieve the target bandwidth (i.e., BW = 200 nm, see Equation S39).

Two major modifications are made to the tunable waveguide to improve its tunability: first, the width of the heater is reduced from W_{ht} = 7 μm to 2.5 μm; and second, the heating region is isolated by the trenches in the SiO_2 cladding and the partial undercut in the Si substrate. Figures S23a and S23b show the calculated temperature distributions with the electric power of P = 100 mW applied to the heater. It can be found that the energy is condensed with the downsize and isolation of the heating region. The isolation width is set as W_{iso} = 3 μm to ensure an easy fabrication. The undercut technology has been demonstrated in Ref. S48. In

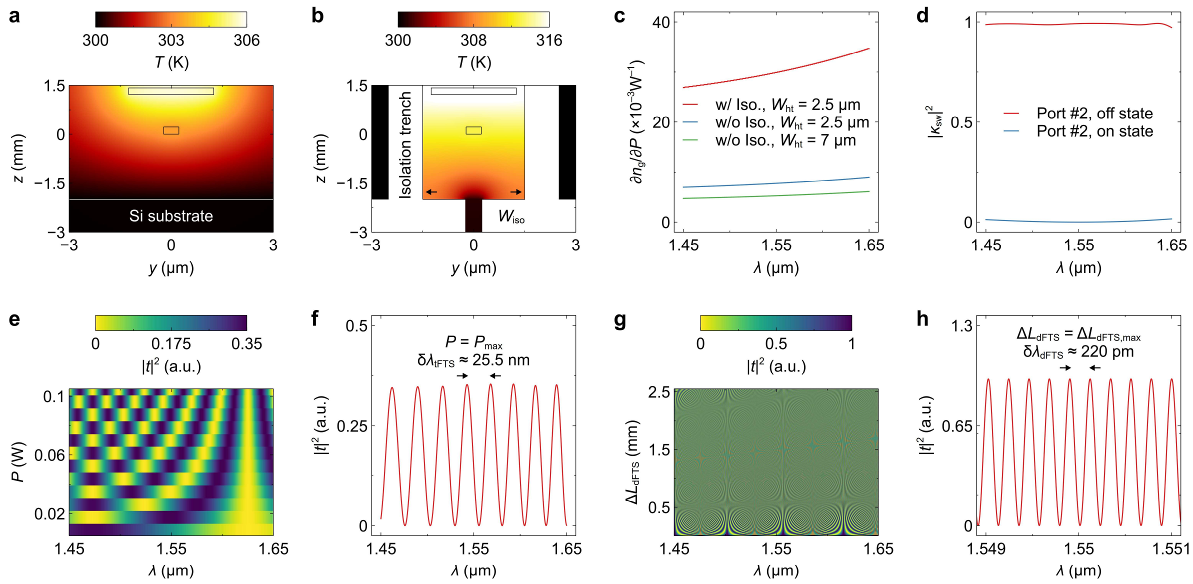

Figure S23 Design of key components. Calculated temperature (T) distributions (**a**) with and (**b**) without isolation trenches. Here, the heating power is set as P = 100 mW. (**c**) Calculated tuning efficiencies ($\partial n_g/\partial P$) at varying wavelengths (λ). Here, the $\partial n_g/\partial P$ curves are compared with different heater widths (W_{ht}) and isolation conditions. (**d**) Calculated coupling ratios ($|\kappa_{SW}|^2$) of the switch at on and off states. (**e**) Calculated transmittance ($|t|^2$) matrix of the tunable Fourier-transform spectrometer (tFTS) with varying P. (**f**) Calculated $|t|^2$ dispersions of the tFTS at $P = P_{max}$. The tFTS resolution is $\delta\lambda_{tFTS}$ = 25.5 nm. (**g**) Calculated $|t|^2$ matrix of the digital Fourier-transform spectrometer (dFTS) with varying arm-length differences (ΔL_{dFTS}). (**h**) Calculated $|t|^2$ dispersions of the dFTS at $\Delta L_{dFTS} = \Delta L_{dFTS,max}$. The dFTS resolution is $\delta\lambda_{tFTS}$ = 220 pm.

Figure S23c, we show the calculated tuning-efficiency ($\partial n_g/\partial P$) dispersions with different W_{ht} and isolation conditions. At the central wavelength, the tuning efficiency is improved from $\partial n_g/\partial P \approx 5.25\times10^{-3}$ W^{-1} to 29.9 $\times10^{-3}$ W^{-1}. The tunability is improved by around one order of magnitude, as a conjunct result of high $\partial n_g/\partial P$ and light-path folding. From Equations S39 and S40, the critical heating power and sweep steps are derived as P_{max} = 105 mW and N_{tFTS} = 16, respectively. As discussed in Figures S8 and S9, the number of sweep steps can be reduced to < 2·BW/$\delta\lambda_{tFTS}$, due to the peculiar folding property of a 2D Fourier map. Here, the optimal sweep steps are N_{tFTS} = 12. The same waveguide structure is used in the MZI switch. The length of the heating section is set as L_{SW} = 200 μm. The switch power is derived as $P_{SW} \approx$ 2 mW. The aggregate power consumption of the modified 2D-FTS is $P_{max} + N_{SW}\cdot P_{SW} \approx$ 120 mW. Figure S23d shows the calculated coupling ratios ($|\kappa_{SW}|^2$) of the switch at on and off states. High extinction ratios are attained over the wavelength range from λ = 1.45 μm to 1.65 μm. Thus, all essential parameters have been determined. In Figures S23e-S23h, we calculate the transmission responses of the tFTS and dFTS. At $\lambda \approx$ 1.55 μm, spectral resolutions are calculated to be $\delta\lambda_{tFTS}$ ≈ 25.5 nm and $\delta\lambda_{dFTS}$ ≈ 220 nm. The obtained resolutions are slightly finer than the target values, as a longer $\Delta L_{dFTS,max}$ and a higher P_{max} are used to counterbalance the resolution dispersion (see Figure S5).

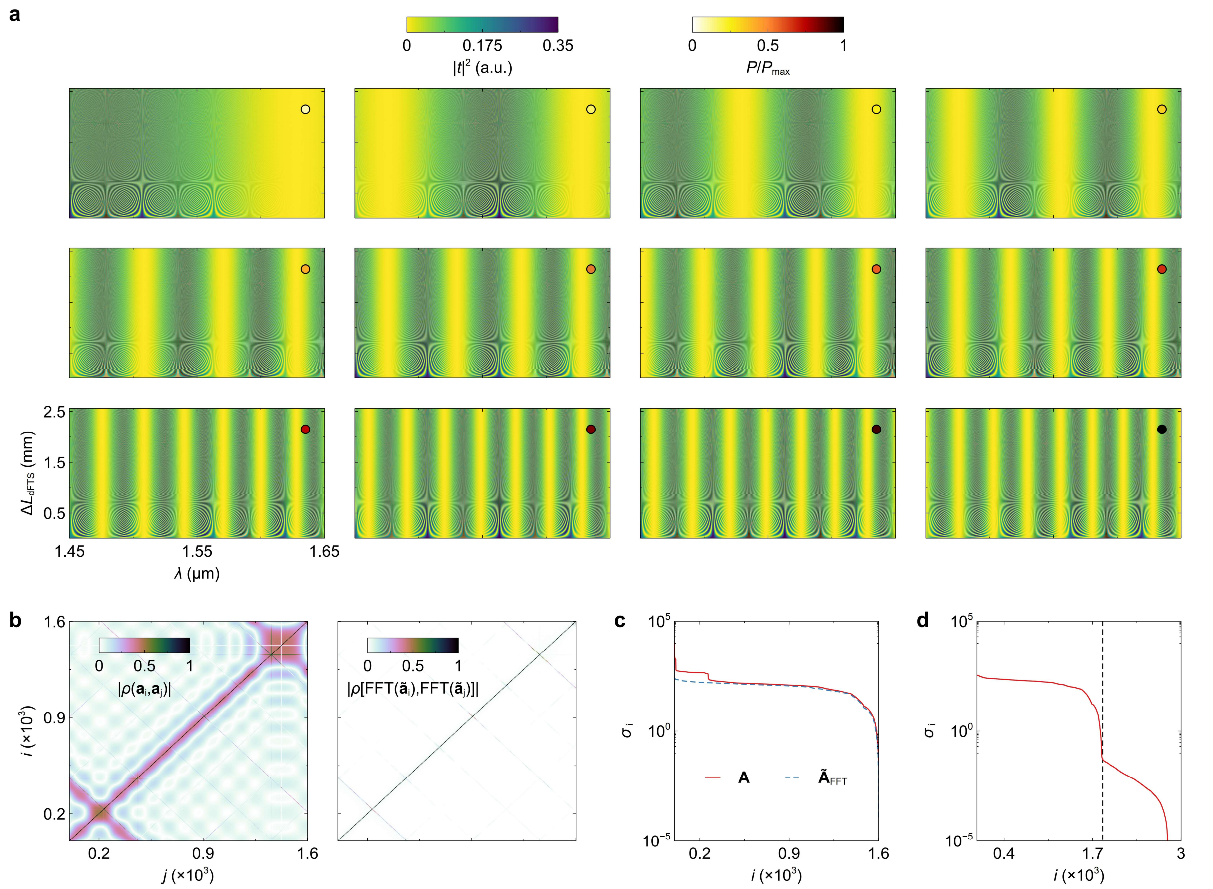

Figure S24 Analysis of the spectrometer. (**a**) Calculated transmittance ($|t|^2$) cube. The cube is sliced into the matrices with varying heating power (P), represented by the colors of dots in the upper right corner. Each matrix contains transmittances with varying arm-length differences (ΔL_{dFTS}) and wavelengths (λ). (**b**) Correlation matrices derived from the cube. On the left panel, the correlation [$\rho(\cdot, \cdot)$] is performed between the fringes ($\mathbf{a}_i$) at different channels. The right panel shows the correlation of the fast Fourier transform (FFT) of fringes ($\tilde{\mathbf{a}}_i$) with zero-frequency components removed. (**c**) Calculated singular values (σ_i). Here, we compare the σ_i curves for the full cube (**A**) and the cube with the zero-frequency components removed ($\tilde{\mathbf{A}}_{\mathrm{FFT}}$). (**d**) Calculated σ_i curve derived from an oversampled cube. The dashed line represents the location of the kink.

The transmittance cube (**A**) of the 2D-FTS is shown in Figure S24a. The left panel of Figure S24b shows the correlation matrix [i.e., $\rho(\cdot, \cdot)$] of **A**. The non-diagonal elements with relatively high correlations originate from the projection effect, as discussed in Figures S7-S9. These high-correlation shades can be inhibited by omitting the zero-frequency components (see the right panel of Figure S24b). In Figure S24c, we show the singular values (σ_i) of the cubes before and after the component removal. The removal operation eliminates the kink and results in a smooth and flat σ_i curve. When the cube is oversampled into > 3000 channels, its σ_i curve levels off at i > 1800. These results demonstrate that the 2D-FTS supports a capacity of N_{2f} > 1601 and that all channels are highly decorrelated. Given the bandwidth of BW = 200 nm, the attainable resolution is thus derived as $\delta\lambda_{2f}$ = 125 pm. We provide some numerical examples to verify the reconstruction capability, as shown in Figure S25. The environmental perturbations are emulated using the method discussed in Note

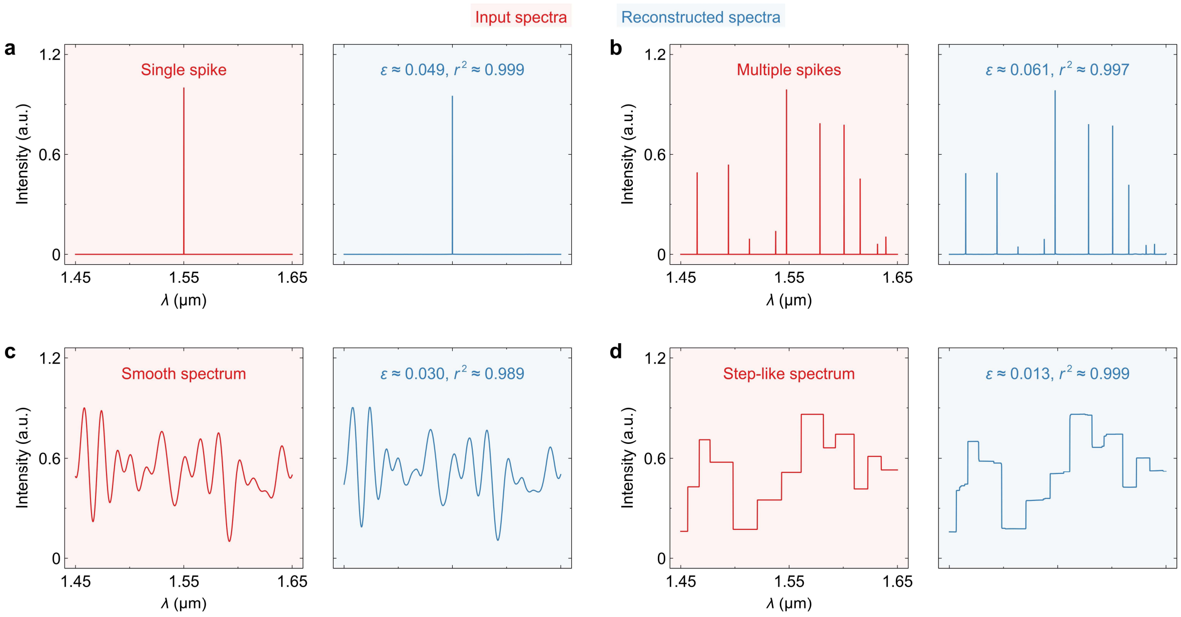

Figure S25 Spectrum reconstruction. Reconstruction of (**a**) a single spike, (**b**) multiple spikes, (**c**) a smooth spectrum, and (**d**) a step-like spectrum. The input and reconstructed spectra are shaded in red and blue, respectively. The relative errors (ε) and coefficients of determination (r^2) are also labeled.

7. Testing spectra with various features are retrieved with high accuracy ($\varepsilon < 0.1$, $r^2 > 0.99$).

B. Three-dimensional Fourier-transform spectrometer

Based on the modified 2D-FTS scheme, scaling to a finer resolution requires a larger arm-length difference in the dFTS, which in turn increases the switch steps or heating power to offset the decrease in FSR_{dFTS}. It is possible to introduce more switchable units in the delay lines to expand FSR_{dFTS}; however, this will lead to a longer acquisition period and a complex switch topology. Here, the concept of 2D-FTS is extended to three dimensions to enhance performance. Figure S26a shows the schematic layout of the 3D-FTS. The device is a three-stage structure that consists of a tFTS, a dFTS, and a SHS. The core idea is to use the dFTS to bridge the resolution-FSR gap between the tFTS and SHS. An ultra-fine resolution can be easily attained by choosing a longer arm-length difference (ΔL_{SHS}) in the SHS. To ensure an acceptable etendue level, we choose to use a small number of output ports (N_{SHS}), resulting in a narrow free spectral range (FSR_{SHS}). On the other hand, given a low heating power, the tFTS supports a broad bandwidth but a rather coarse resolution ($\delta\lambda_{tFTS}$) that cannot cover FSR_{SHS}. The dFTS has a modest resolution ($\delta\lambda_{dFTS}$) and free spectral range (FSR_{dFTS}), serving as an interface between the fine-resolution, narrow-FSR SHS and the coarse-resolution, broad-FSR tFTS. Thus, all channels can be decorrelated as long as the following condition can be satisfied:

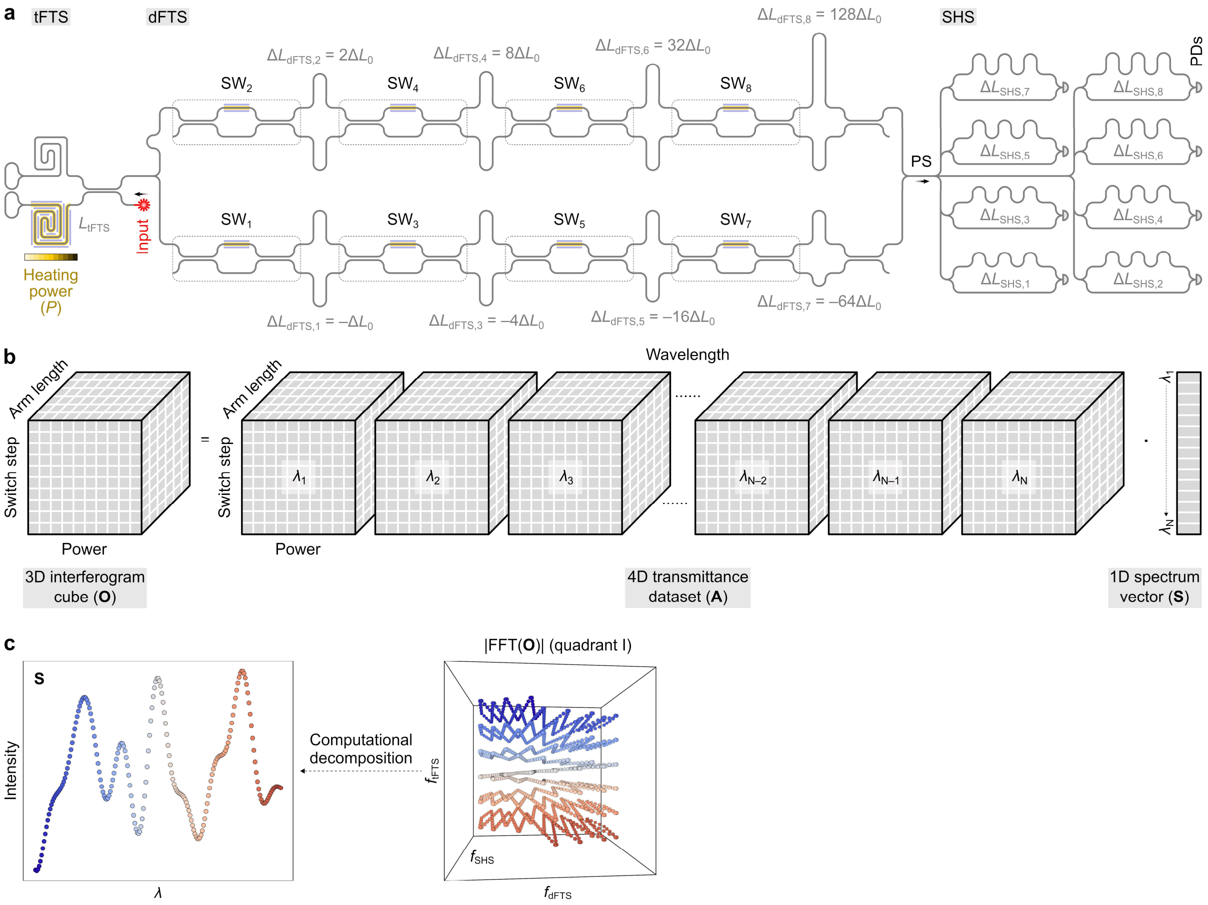

Figure S26 Three-dimensional Fourier-transform spectrometer (3D-FTS). (**a**) Schematic layout of the 3D-FTS. The device consists of a tunable FTS (tFTS), a digital FTS (dFTS), and a spatial heterodyne spectrometer (SHS). The SHS has a large arm-length difference ($\Delta L_{SHS,max}$) but a small number of output ports, thereby supporting a ultra-fine resolution but a narrow free spectral range (FSR). The tFTS, on the other hand, has the ability to attain an ultra-broad bandwidth, but its resolution is constrained by the heating power (P). The fine-resolution SHS and broadband tFTS are bridged by a dFTS with a modest resolution and FSR. (**b**) Light transmission in the 3D-FTS. The transmission responses of the 3D-FTS can be depicted by a 4D dataset (denoted as **A**), wherein each frame is a 3D fringe with variations of heating power in the tFTS, switch steps in the dFTS, and arm lengths in the SHS. Given a 1D spectrum vector (denoted as **S**), the output interferogram (denoted as **O**) is a 3D cube as a combination of fringes. (**c**) Reconstruction principle. The 3D fast Fourier transform (FFT) of a fringe is a spot at a distinctive location. Therefore, in the Fourier domain, an interferogram is mapped to a cluster of spots scattered in three dimensions (i.e., f_{tFTS}, f_{dFTS}, and f_{SHS}). When the wavelength is tuned over the whole bandwidth, a spot oscillates rapidly between f_{SHS} = 0 and 1/2, meander slowly between f_{dFTS} = 0 and 1/2, and shifts from f_{SHS} = 1/2 to 0. The shift direction depends on the phase at the first sampling step. From the serpentine trajectory, the spectrum can be recovered via computational decomposition. SW, switch. PS, power splitter. PD, photodetector.

$$\delta\lambda_{tFTS} < FSR_{dFTS}, \tag{S41}$$

$$\delta\lambda_{dFTS} < FSR_{SHS}. \tag{S42}$$

The resolution and bandwidth of the 3D-FTS are respectively determined by the resolution of the SHS ($\delta\lambda_{tFTS}$) and the free spectral range of the tFTS (FSR_{tFTS}):

$$\delta\lambda_f = \delta\lambda_{SHS}, \tag{S43}$$

$$\mathrm{BW} = \mathrm{FSR}_{\mathrm{tFTS}}. \tag{S44}$$

The derivations of $\delta\lambda_{\mathrm{SHS}}$ and $\mathrm{FSR}_{\mathrm{tFTS}}$ can be found in Section A and Note 6. The 3D-FTS provides the following advantages:

1. A finer resolution ($\delta\lambda_{2f}$ < 31.25 pm) can be achieved across a 200-nm bandwidth.
2. The cost of performance enhancement is moderate. The use of the SHS only yields few additional ports and a slight reduction in output etendue, which is deemed acceptable in most applications.
3. The spectrometer is built with the components (i.e., tFTS, dFTS, and SHS) that are well-established and verified.

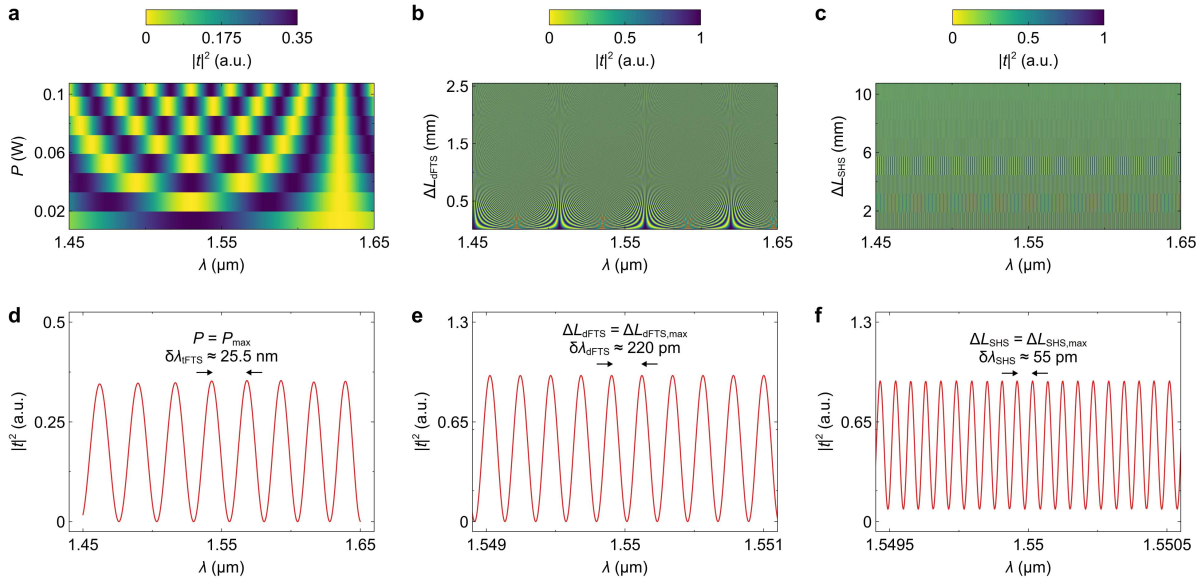

Figure S27 Transmission responses of spectrometers. Calculated transmittance ($|t|^2$) matrices of the (**a**) tunable Fourier-transform spectrometer (tFTS), (**b**) digital Fourier-transform spectrometer (dFTS), and (**c**) spatial heterodyne spectrometer (SHS). Calculated $|t|^2$ dispersions of the (**d**) tFTS, (**e**) dFTS, and (**f**) SHS with maximum asymmetries. The spectral resolutions are also labeled.

Here, the designs of the tFTS and SHS are identical to those in previous discussions (see Section A). The SHS is based on the structure shown in Figure 1. The maximum arm-length difference of the SHS is chosen as $\Delta L_{\mathrm{SHS,max}}$ = 10.2 mm to attain a resolution of $\delta\lambda_{2f}$ = 31.25 pm across the entire bandwidth. The number of MZIs is then set as N_{SHS} = 8 to satisfy Equation S42 and ensure a relatively high etendue. Moreover, the power sweep steps of the tFTS are reduced to N_{tFTS} = 8. The feasibility of N_{tFTS} reduction will be discussed later. In Figures S27a-S27c, we respectively show the calculated transmittance matrices of the tFTS, dFTS, and SHS. The 4D transmittance dataset of the 3D-FTS can be obtained by reorganizing these matrices. Figures S27d-S27f show the calculated transmittance dispersions of the spectrometers with maximum asymmetries. We then derive the correlation matrices from the flattened 4D dataset of the 3D-FTS, as shown in the left panels

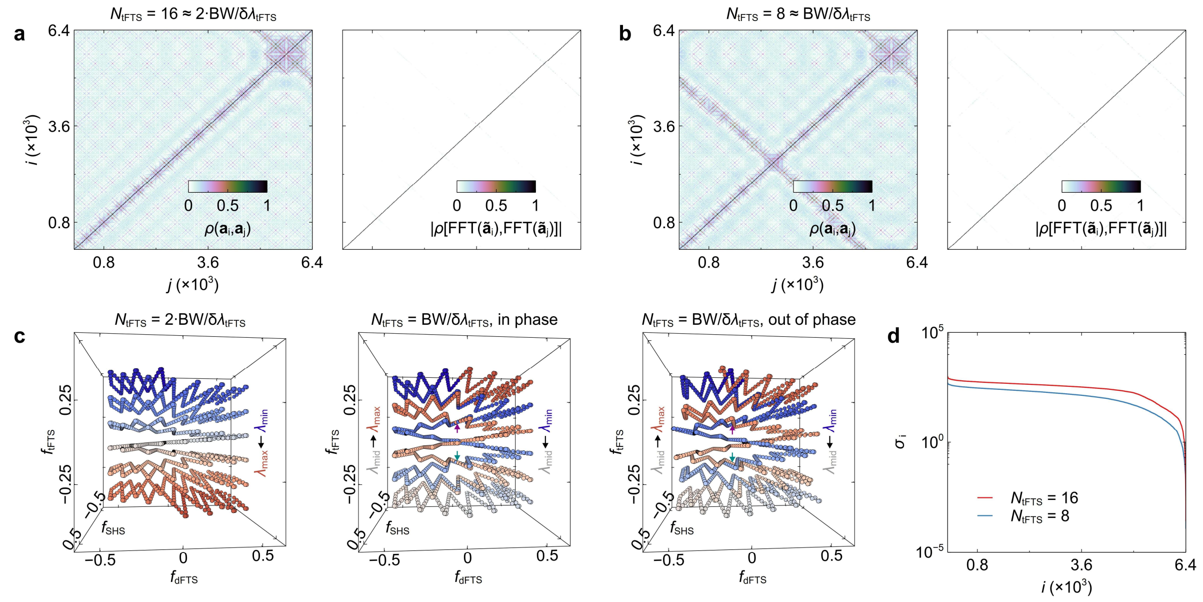

Figure S28 Analysis of the spectrometer. Correlation matrices derived from the dataset with power sweep steps of (**a**) N_{tFTS} = 16 ≈ 2·BW/δλ_{tFTS} and (**b**) 8 ≈ BW/δλ_{tFTS}. On the left panel, the correlation [ρ(·, ·)] is performed between the fringes ($\mathbf{a}_i$) at varying wavelengths. The right panel shows the correlation of the fast Fourier transform (FFT) of fringes ($\tilde{\mathbf{a}}_i$) with zero-frequency components omitted. (**c**) Illustrations of Fourier maps with N_{tFTS} = 2·BW/δλ_{tFTS} and BW/δλ_{tFTS}. (**d**) Calculated singular values (σ_i) with N_{tFTS} = 16 and 8.

of Figures S28a and S28b. Here, we compare the correlation matrices with N_{tFTS} = 16 ≈ 2·BW/δλ_{tFTS} and 8 ≈ BW/δλ_{tFTS}. Some non-diagonal element has relatively high correlations, mainly due to the projection effect, as discussed Figures S7-S9. Similar to 2D-FTS, these "shades" can be sufficiently depressed after the removal of zero-frequency components, as shown in the right panels of Figures S28a and S28b. Notably, compared to 2D-FTS (see Figure S24), the 3D-FTS exhibits a significantly lower level of residual correlations even with a reduced number of sweep steps (i.e., N_{tFTS} = 8). According to the analysis in Note 6, the component removal cannot fully address the correlation issue at the crossover locations in a folded 2D Fourier map, which does not hold valid in the 3D case. Figure S28c shows the 3D Fourier maps with different N_{tFTS}. When the sweep steps are set as N_{tFTS} = 2·BW/δλ_{tFTS}, all mapping spots are completely unfolded at distinctive locations. With the sweep steps reduced to half (i.e, N_{tFTS} = BW/δλ_{tFTS}), the Fourier map is folded once along f_{tFTS}, leading to few crossovers (< N_{tFTS}/2) between forward and backward trajectories (see the arrows in the middle panel). Such crossovers only occur when the initial phase of the tFTS is an integer quotient of 2π. Due to waveguide dispersions, however, the tFTS is naturally out of phase at the first sampling, and all crossovers can be thus circumvented (see the arrows in the right panel). Thereby, the 3D-FTS has the potential to deploy a greater number of channels in a 3D Fourier space while preventing inter-channel correlations. Figure S28d shows the calculated singular values (σ_i) with N_{tFTS} = 16 and 8. The decay rates of two curves are virtually identical.

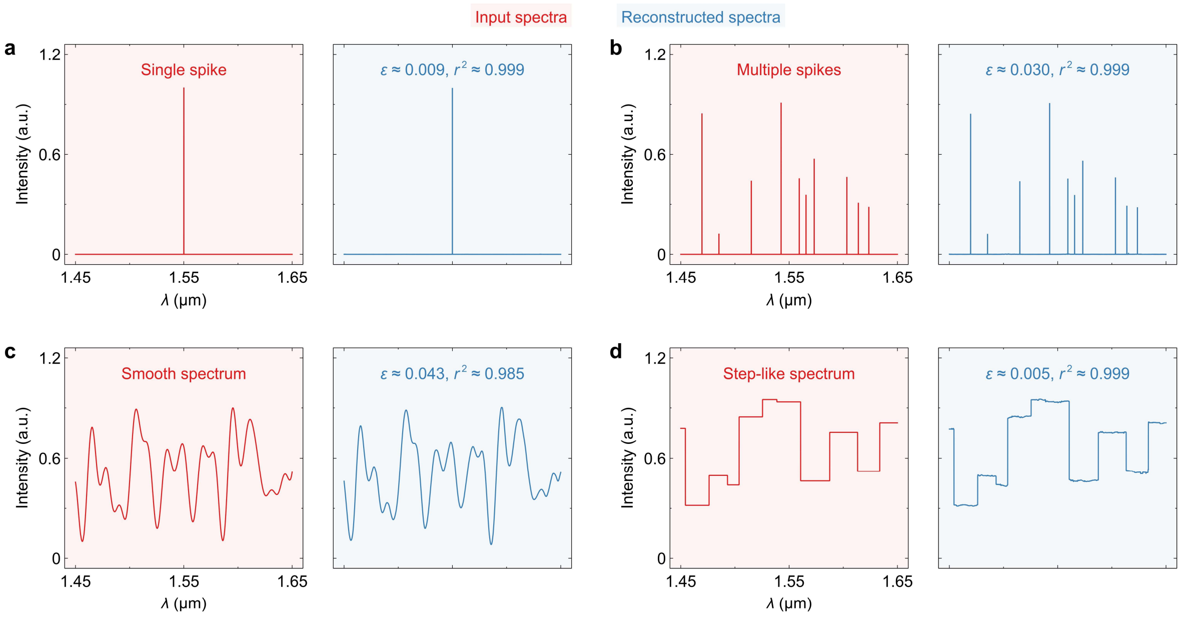

Figure S29 Spectrum reconstruction. Reconstruction of (**a**) a single spike, (**b**) multiple spikes, (**c**) a smooth spectrum, and (**d**) a step-like spectrum. The input and reconstructed spectra are shaded in red and blue, respectively. The relative errors (ε) and coefficients of determination (r^2) are also labeled.

There are no discernible kinks from i = 1 to 6401, demonstrating the channel capacity of N_{2f} = 6401 and the corresponding resolution of $\delta\lambda_{2f}$ = 31.5 pm over BW = 200 nm. We also give some numerical reconstruction examples, as shown in Figure S29. More details of the reconstruction method can be found in Note 8. Small errors are achieved for various testing spectra.

C. Higher-dimensional Fourier-transform spectrometer

In above sections, we have discussed a modified 2D-FTS with improved power efficiency and a 3D-FTS with an ultra-fine spectral resolution. In this section, we will further extend these concepts and provide a generic design framework for higher-dimensional Fourier-transform spectrometry (HD-FTS). Figure S30 shows the conceptual illustration of a N-dimensional FTS. The structure is composed of N-stages of FTSs cascaded in

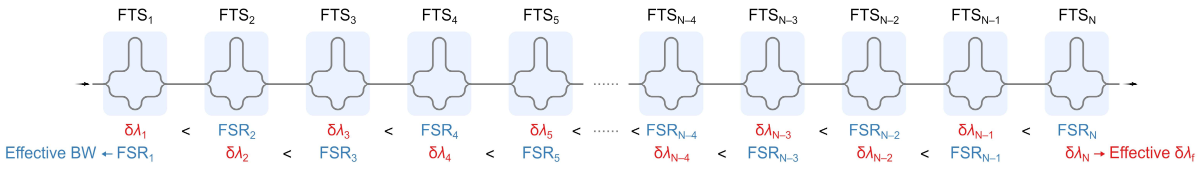

Figure S30 Higher-dimensional Fourier-transform spectrometer (HD-FTS). Conceptual illustration of a HD-FTS. A N-dimensional FTS can be realized by cascading N stages of FTSs, as long as the resolution of FTS_i is finer than the free spectral range of FTS_{i+1}, i.e., $\delta\lambda_i < FSR_{i+1}$. Here, FTS_i denotes the i-th spectrometer unit. The effective resolution is determined by the resolution of the last unit, i.e., $\delta\lambda_f = \delta\lambda_N$, while the effective bandwidth is determined by the free spectral range of the first unit, i.e., BW = FSR_1. The interferogram, which is a N-dimensional dataset, can be obtained by sweeping all units in a nested loop.

succession. Light is launched at the first unit (i.e., FTS_1), and the signal is captured at the last unit (i.e., FTS_N). By tuning the sinusoidal responses of all FTS units in a nested loop, a *N*-dimensional interferogram can be generated. All channels can be decorrelated when the resolution of FTS_i is finer than the free spectral range of FTS_{i+1}:

$$\delta\lambda_i < FSR_{i+1}. \tag{S45}$$

The effective resolution thus becomes the resolution of the FTS_N, i.e., $\delta\lambda_f = \delta\lambda_N$, while the effective bandwidth becomes the free spectral range of the FTS_1, i.e., $BW = FSR_1$. Based on this scheme, each wavelength channel will be mapped to a spot in a *N*-dimensional Fourier space, and any arbitrary spectrum can be reconstructed using the method proposed in Note 8.

This design strategy has the following advantages:

1. All conventional FTSs suffer from an inherent trade-off between resolutions and bandwidths due to the difficulty in achieving substantial phase change in a nanophotonic circuit. Most prior studies focus on increasing the phase variation range of delay lines, e.g., Refs. S41 and S43, but have limited scalability. The resolution-bandwidth limit is circumvented with the method proposed in this work. Utilizing HD-FTS prevents the needs for an FTS with both a fine resolution and a broad bandwidth. Instead, it only requires a fine-resolution, narrow-FSR FTS at the input end and a coarse-resolution, broad-FSR FTS at the output end, while using FTSs with modest resolutions and FSRs to bridge the two ends, making it easier to scale to higher performance.
2. For the first time, we reveal the connection between cascaded FTSs and high-dimensional Fourier transform. Unlike the vernier scheme with cascaded narrow-linewidth filters[S11], the HD-FTS does not require stringent wavelength alignment between FTS units. As long as Equation S45 is fulfilled, all wavelength channels can be decorrelated and allocated to distinct locations in a *N*-dimensional Fourier space.
3. The HD-FTS is a flexible scheme that can be utilized to boost the performance of any types of FTSs. For instance, by employing a highly asymmetric, single-port FTS at the output end, it is possible to preserve both Fellgett's and Jacquinot's advantages, while achieving an ultra-fine resolution.

Table S3 summarizes the performance of 2D- and 3D-FTSs discussed in this work.

Table S3. Comparison of proposed higher-dimensional Fourier-transform spectrometers.

Design	Component	P_a [W]	N_{port}	N_{2f}	BW [nm]	$\delta\lambda_{2f}$ [pm]
2D-FTS (Exp.)	tFTS, SHS	2.4	128	1601	200	125
2D-FTS (Sim.)	tFTS, dFTS	0.12	1	6401	200	125
3D-FTS (Sim.)	tFTS, dFTS, SHS	0.12	8	6401	200	31.25

Exp., experimental result.

Sim., simulation result.

FTS, Fourier-transform spectrometer.

SHS, spatial heterodyne spectrometer.

tFTS, tunable FTS.

dFTS, digital FTS.

P_a, aggregate power consumption.

N_{port}, number of output physical ports.

N_{2f}, number of wavelength channels.

BW, working bandwidth.

$\delta\lambda_{2f}$, spectral resolution.

Note 11. Comparison of spectrometry schemes

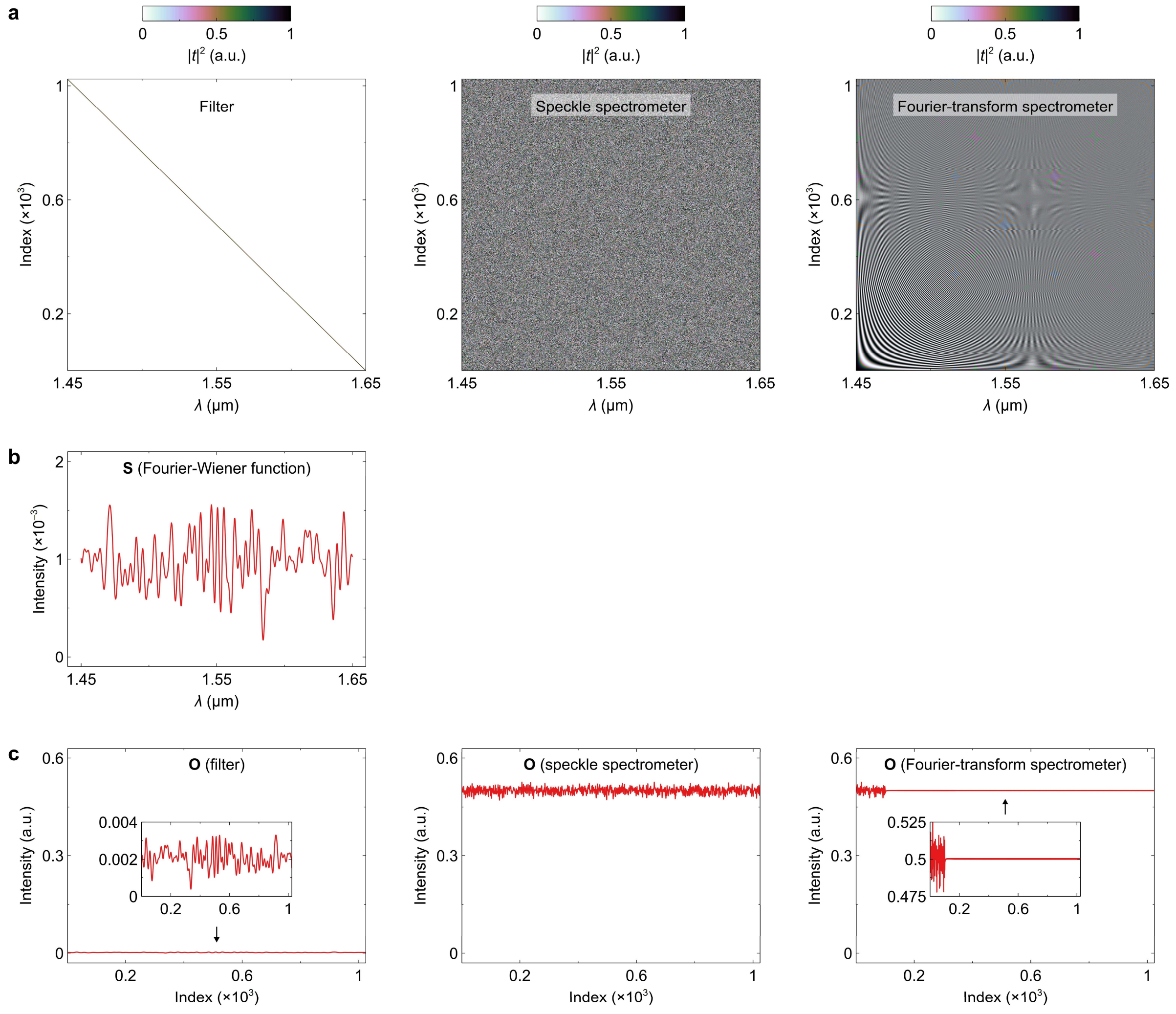

Figure S31 Comparison of spectrometry schemes. (**a**) Calculated transmittance matrices of a filter, a speckle spectrometer, and a Fourier-transform spectrometer. (**b**) Testing random spectrum (denoted as **S**) generated with the Fourier-Wiener function. (**c**) Output interferograms (denoted as **O**). The insets show the enlarged views of **O**.

As discussed in Note 1, there are three mainstream approaches in integrated spectrometry, i.e., the narrow-linewidth filter, speckle spectrometer, and Fourier-transform spectrometer (FTS). The demonstrated 2D-FTS has a record large channel capacity (i.e., $N_{2f} > 1601$) among all reported FTSs. A fine resolution of $\delta\lambda_{2f} <$ 125 pm and a broad bandwidth of BW = 200 nm are experimentally accomplished. Comparable resolutions and bandwidths can also be attained using other schemes, e.g., filters and speckle spectrometers (see Table S1). Nevertheless, it is imperative to consider the signal-to-noise ratio (SNR) when making a comprehensive evaluation of resolutions and bandwidths. Among all reported schemes, FTSs have the potential to achieve the highest SNR at the same number of resolvable channels due to the Fellgett's and Jacquinot's advantages. Here, we give a numerical example to show the difference in spectrometry mechanisms. Figure S31a shows

the transmittance matrices of a filter, a speckle spectrometer, and an FTS. Here, a quasi-diagonal matrix, a chaotic matrix, and a matrix formed by sinusoidal vectors are used to emulate spectrometer responses. All these matrices are sized 1024×1024, as an example. In Figure S31b, a random testing spectrum (denoted as **S**) is generated with Fourier-Wiener series (see Equations S26-S28 for explanations). Here, **S** is normalized to its integral energy. The output signals (denoted as **O**) are shown in Figure S31c. It can be found that the signal intensities are much lower in a filter since it only selects a single channel at each sampling, resulting in a worse SNR. To counteract the degradation in received etendue, it usually requires a longer integral time and extra signal amplification. The speckle spectrometer and FTS capture all channels at each sampling; as a result, the average of **O** is half of the launched amount, thereby ensuring a high signal detectivity. Given a continuous spectrum, the speckle spectrometer has chaotic output over all sampling steps. For the FTS, the effective information of a continuous spectrum can be collected with a small portion of sampling steps (see the inset), while the output of other samplings levels off to ≈ 0.5. This phenomenon can be attributed to the scarcity of high-frequency components in most continuous spectra. For an FTS, the temperature sensitivity is stronger with a larger arm asymmetry; according to the result shown in Figure S31c, however, the phase shift at a high-frequency sampling only causes minor deviations in **O**, which mitigates the impact of thermo-optical noises. Therefore, the FTS offers greater potential to support a higher SNR, especially for continuous spectra.

Table S4. Comparison of signal-to-noise ratios.

Design	N_{port}	N_{ch}	BW [nm]	$\Delta\lambda_{res}$ [pm]	PSNR [dB]	Noise floor [dB]
Filter[S11]	10	1941	10	5	NM [(a)]	≈ −10 [(b)]
SS[S26]	4	> 3800	115	30	27.5 [(c)]	> −20 [(d)]
tFTS & MRR[S48]	1	> 190	90	470	≈ 10 [(e)]	≈ −10 [(f)]
**2D-FTS (this work)**	**128**	**1601**	**200**	**125**	**> 25 [(g)]**	**−35 ~ −40 [(h)]**

SS, speckle spectrometer

tFTS, tunable Fourier-transform spectrometer.

2D-FTS, two-dimensional Fourier-transform spectrometer.

MRR, micro-ring resonator.

N_{port}, number of output physical ports.

N_{ch}, number of wavelength channels.

BW, working bandwidth.

$\delta\lambda_{res}$, spectral resolution.

PSNR, peak signal-to-noise ratio.

[(a)]NM, not mentioned.

[(b)]From Figure 6 in Ref. S11.

[(c)]From Figure 5c in Ref. S26.

[(d)]From Figure 4e in Ref. S26.

[(e)]From Figure 5b in Ref. S48.

[(f)]From Figure 5b in Ref. S48.

[(g)]From Figures 5c and 5d in the main manuscript.

[(h)]From Figure 5c in the main manuscript.

A comparison in SNRs is provided to support this viewpoint (see Table S4). Here, we mainly compare some typical designs with comparable capacities, as the number of solvable wavelength channels will affect reconstruction accuracy. We present two sets of performance indicators: the peak SNR (i.e., PSNR), which depicts the maximum contrast between two reconstructed peaks, and the noise floor, which is the maximum of background false signals. In Table S4, the PSNRs and noise floors are either specified in the research or estimated from testing spectra (see the footnote). In Ref. S11, a spectrometer is realized with a high-Q microring resonator (MRR). The false peaks reach ≈ –10 dB in the reconstruction of the response of a fiber Bragg grating. Similarly, when the FTS is combined with a MRR[S48], the achievable PSNR and noise floor degrade to ≈ 10 dB and ≈ –10 dB, respectively, since the use of a narrow-linewidth filter will negate both Fellgett's and Jacquinot's advantages. Hence, it is evident that the filter-based scheme has an inherent limit in attaining a high SNR at a fine resolution. The speckle spectrometer in Ref. S26 demonstrates a PSNR of ≈ 27.5 dB and a noise floor of ≈ –20 dB. By comparison, our proposed 2D-FTS has a comparable PSNR (≈ 25 dB) and a much lower noise floor (≈ –35 ~ –40 dB). The device demonstrated in this work has 128 physical ports, and the received etendue is enhanced by around one order of magnitude compared to a 1601-channel filter. By using the single-port scheme discussed in Note 10, Section A, it is possible to improve the SNR even further while maintaining a fine resolution and a broad bandwidth.

Supplementary references

S1. Cheben P, *et al.* A high-resolution silicon-on-insulator arrayed waveguide grating microspectrometer with sub-micrometer aperture waveguides. *Opt Express* **15**, 2299-2306 (2007).

S2. Xiao M, Mingyu L, Jian-Jun H. CMOS-compatible integrated spectrometer based on Echelle diffraction grating and MSM photodetector array. *IEEE Photon J* **5**, 6600807-6600807 (2013).

S3. Calafiore G, *et al.* Holographic planar lightwave circuit for on-chip spectroscopy. *Light Sci Appl* **3**, e203-e203 (2014).

S4. Xia Z, *et al.* High resolution on-chip spectroscopy based on miniaturized microdonut resonators. *Opt Express* **19**, 12356-12364 (2011).

S5. Cheng Z, *et al.* Generalized modular spectrometers combining a compact nanobeam microcavity and computational reconstruction. *ACS Photon* **9**, 74-81 (2021).

S6. Zhang J, Cheng Z, Dong J, Zhang X. Cascaded nanobeam spectrometer with high resolution and scalability. *Optica* **9**, 517-521 (2022).

S7. Sun C, *et al.* Broadband and high-resolution integrated spectrometer based on a tunable FSR-free optical filter array. *ACS Photon* **9**, 2973-2980 (2022).

S8. Zheng S, *et al.* A single-chip integrated spectrometer via tunable microring resonator array. *IEEE Photon J* **11**, 1-9 (2019).

S9. Zhang Z, Wang Y, Tsang HK. Tandem configuration of microrings and arrayed waveguide gratings for a high-resolution and broadband stationary optical spectrometer at 860 nm. *ACS Photon* **8**, 1251-1257 (2021).

S10. Zhang Z, *et al.* Integrated scanning spectrometer with a tunable micro-ring resonator and an arrayed waveguide grating. *Photon Res* **10**, A74 (2022).

S11. Zhang L, Zhang M, Chen T, Liu D, Hong S, Dai D. Ultrahigh-resolution on-chip spectrometer with silicon photonic resonators. *Opt Electron Adv* **5**, 210100-210100 (2020).

S12. Xu HN, Qin Y, Hu GL, Tsang HK. Integrated single-resonator spectrometer beyond the free-spectral-range limit. *ACS Photon* **10**, 654–666 (2023).

S13. Xu H, Qin Y, Hu G, Tsang HK. Breaking the resolution-bandwidth limit of chip-scale spectrometry by harnessing a dispersion-engineered photonic molecule. *Light Sci Appl* **12**, 64 (2023).

S14. Sun CL, *et al.* Scalable on-chip microdisk resonator spectrometer. *Laser Photon Rev* **17**, 2200792 (2023).

S15. Redding B, Liew SF, Sarma R, Cao H. Compact spectrometer based on a disordered photonic chip. *Nat Photon* **7**, 746-751 (2013).

S16. Hartmann W*, et al.* Waveguide-integrated broadband spectrometer based on tailored disorder. *Adv Opt Mater* **8**, 1901602 (2020).

S17. Hadibrata W, Noh H, Wei H, Krishnaswamy S, Aydin K. Compact, high-resolution inverse-designed on-chip spectrometer based on tailored disorder modes. *Laser Photon Rev* **15**, 2000556 (2021).

S18. Li A, Fainman Y. On-chip spectrometers using stratified waveguide filters. *Nat Commun* **12**, 2704 (2021).

S19. Redding B, Fatt Liew S, Bromberg Y, Sarma R, Cao H. Evanescently coupled multimode spiral spectrometer. *Optica* **3**, 956-962 (2016).

S20. Piels M, Zibar D. Compact silicon multimode waveguide spectrometer with enhanced bandwidth. *Sci Rep* **7**, 43454 (2017).

S21. Yi D, Zhang Y, Wu X, Tsang HK. Integrated multimode waveguide with photonic lantern for speckle spectroscopy. *IEEE J Quant Electron* **57**, 1-8 (2021).

S22. Yi D, Tsang HK. High-resolution and broadband two-stage speckle spectrometer. *J Lightwave Technol* **40**, 7969-7976 (2022).

S23. Zhang Z, Li Y, Wang Y, Yu Z, Sun X, Tsang HK. Compact high resolution speckle spectrometer by using linear coherent integrated network on silicon nitride platform at 776 nm. *Laser Photon Rev* **15**, 2100039 (2021).

S24. Li Y*, et al.* Inverse-designed linear coherent photonic networks for high-resolution spectral reconstruction. *ACS Photon* **10**, 1012-1018 (2023).

S25. Lin Z*, et al.* High-performance, intelligent, on-chip speckle spectrometer using 2D silicon photonic disordered microring lattice. *Optica* **10**, 497 (2023).

S26. Yao C, Chen M, Yan T, Ming L, Cheng Q, Penty R. Broadband picometer-scale resolution on-chip spectrometer with reconfigurable photonics. *Light Sci Appl* **12**, 156 (2023).

S27. Xu H, Qin Y, Hu G, Tsang HK. Cavity-enhanced scalable integrated temporal random-speckle spectrometry. *Optica* **10**, 1177-1188 (2023).

S28. Sun C*, et al.* Integrated microring spectrometer with in-hardware compressed sensing to break the resolution-bandwidth limit for general continuous spectrum analysis. *Laser Photon Rev*, 2300291 (2023).

S29. Okamoto K, Aoyagi H, Takada K. Fabrication of Fourier-transform, integrated-optic spatial heterodyne

spectrometer on silica-based planar waveguide. *Opt Lett* **35**, 2103-2105 (2010).

S30. Fontaine NK, Okamoto K, Su T, Yoo SJ. Fourier-transform, integrated-optic spatial heterodyne spectrometer on a silica-based planar waveguide with 1 GHz resolution. *Opt Lett* **36**, 3124-3126 (2011).

S31. Bock PJ*, et al.* Subwavelength grating Fourier-transform interferometer array in silicon-on-insulator. *Laser Photon Rev* **7**, L67-L70 (2013).

S32. Velasco AV*, et al.* High-resolution Fourier-transform spectrometer chip with microphotonic silicon spiral waveguides. *Opt Lett* **38**, 706-708 (2013).

S33. Herrero-Bermello A*, et al.* On-chip Fourier-transform spectrometers and machine learning: a new route to smart photonic sensors. *Opt Lett* **44**, 5840-5843 (2019).

S34. Podmore H*, et al.* Demonstration of a compressive-sensing Fourier-transform on-chip spectrometer. *Opt Lett* **42**, 1440-1443 (2017).

S35. Yang M, Li M, He JJ. Static FT imaging spectrometer based on a modified waveguide MZI array. *Opt Lett* **42**, 2675-2678 (2017).

S36. Gonzalez-Andrade D*, et al.* Broadband Fourier-transform silicon nitride spectrometer with wide-area multiaperture input. *Opt Lett* **46**, 4021-4024 (2021).

S37. Duong Dinh TT*, et al.* Mid-infrared Fourier-transform spectrometer based on metamaterial lateral cladding suspended silicon waveguides. *Opt Lett* **47**, 810-813 (2022).

S38. Yoo KM, Chen RT. Dual-polarization bandwidth-bridged bandpass sampling Fourier transform spectrometer from visible to near-infrared on a silicon nitride platform. *ACS Photon* **9**, 2691-2701 (2022).

S39. Lu L, Zhang H, Li X, Chen J, Zhou L. Low temperature sensitivity on-chip Fourier-transform spectrometer based on dual-layer Si3N4 spiral waveguides. *Photon Res* **11**, 591-599 (2023).

S40. Li J, Lu DF, Qi ZM. Miniature Fourier transform spectrometer based on wavelength dependence of half-wave voltage of a LiNbO(3) waveguide interferometer. *Opt Lett* **39**, 3923-3926 (2014).

S41. Souza M, Grieco A, Frateschi NC, Fainman Y. Fourier transform spectrometer on silicon with thermo-optic non-linearity and dispersion correction. *Nat Commun* **9**, 665 (2018).

S42. Li A, Fainman Y. Integrated slicon Fourier transform spectrometer with broad bandwidth and ultra-high resolution. *Laser Photon Rev* **15**, 2000358 (2021).

S43. Kita DM*, et al.* High-performance and scalable on-chip digital Fourier transform spectroscopy. *Nat Commun* **9**, 4405 (2018).

S44. Du J*, et al.* High-resolution on-chip Fourier transform spectrometer based on cascaded optical

switches. *Opt Lett* **47**, 218-221 (2022).

S45. le Coarer E*, et al.* Wavelength-scale stationary-wave integrated Fourier-transform spectrometry. *Nat Photon* **1**, 473-478 (2007).

S46. Nie X, Ryckeboer E, Roelkens G, Baets R. CMOS-compatible broadband co-propagative stationary Fourier transform spectrometer integrated on a silicon nitride photonics platform. *Opt Express* **25**, A409-A418 (2017).

S47. Pohl D*, et al.* An integrated broadband spectrometer on thin-film lithium niobate. *Nat Photon* **14**, 24-29 (2019).

S48. Zheng SN*, et al.* Microring resonator-assisted Fourier transform spectrometer with enhanced resolution and large bandwidth in single chip solution. *Nat Commun* **10**, 2349 (2019).

S49. Xu H, Dai D, Shi Y. Fabrication-friendly on-chip silicon polarizer based on polarization-selective corner mirrors. *IEEE Photon Technol Lett* **33**, 652-655 (2021).

S50. Florjanczyk M, Cheben P, Janz S, Scott A, Solheim B, Xu DX. Multiaperture planar waveguide spectrometer formed by arrayed Mach-Zehnder interferometers. *Opt Express* **15**, 18176-18189 (2007).

S51. Roberts RA, Mullis CT. *Digital signal processing*. Addison-Wesley Longman Publishing Co., Inc. (1987).

S52. FerrePikal ES*, et al.* IEEE standard definitions of physical quantities for fundamental frequency and time metrology---random instabilities. *Proceedings of the 1997 Ieee International Frequency Control Symposium*, 338-357 (1997).

S53. IEEE standard specification format guide and test procedure for single-axis interferometric fiber optic gyros. In: *IEEE Std 952-1997* (1998).

S54. Welch P. The use of fast Fourier transform for the estimation of power spectra: A method based on time averaging over short, modified periodograms. *IEEE Transactions on Audio and Electroacoustics* **15**, 70-73 (1967).

S55. Hamming RW. *Digital filters*. Courier Corporation (1998).

S56. Grotevent MJ*, et al.* Integrated photodetectors for compact Fourier-transform waveguide spectrometers. *Nat Photon* **17**, 59-64 (2023).

S57. Hansen PC. *Rank-deficient and discrete ill-posed problems: numerical aspects of linear inversion*. SIAM (1998).

S58. Hansen PC, Nagy JG, O'leary DP. *Deblurring images: matrices, spectra, and filtering*. SIAM (2006).

S59. Jensen TL, Jørgensen JH, Hansen PC, Jensen SH. Implementation of an optimal first-order method for

strongly convex total variation regularization. *BIT Numerical Mathematics* **52**, 329-356 (2011).

S60. Becker S, Bobin J, Candès EJ. NESTA: a fast and accurate first-order method for sparse recovery. *SIAM Journal on Imaging Sciences* **4**, 1-39 (2011).

S61. Buccini A, Donatelli M, Reichel L. Iterated Tikhonov regularization with a general penalty term. *Numerical Linear Algebra with Applications* **24**, e2089 (2017).

S62. Golub GH, Heath M, Wahba G. Generalized cross-validation as a method for choosing a good ridge parameter. *Technometrics* **21**, 215-223 (1979).

S63. Gazzola S, Hansen PC, Nagy JG. IR Tools: a MATLAB package of iterative regularization methods and large-scale test problems. *arXiv preprint arXiv:171205602* (2017).

S64. Ravasi M, Vasconcelos I. PyLops—A linear-operator Python library for scalable algebra and optimization. *SoftwareX* **11**, 100361 (2020).

S65. Hansen PC. The discrete Picard condition for discrete ill-posed problems. *BIT Numerical Mathematics* **30**, 658-672 (1990).

S66. Filip S, Javeed A, Trefethen LN. Smooth random functions, random ODEs, and Gaussian processes. *SIAM Review* **61**, 185-205 (2019).
